# Palladium-Catalyzed Heteroannulation of Bdan-Capped Alkynes: Rapid Access to Complex Indole Scaffolds

**DOI:** 10.1021/acs.joc.5c01781

**Published:** 2025-09-15

**Authors:** Dean D. Roberts, John M. Halford-McGuff, Marek Varga, Aidan P. McKay, Allan J. B. Watson

**Affiliations:** EaStCHEM, School of Chemistry, 150654 University of St Andrews, North Haugh, St Andrews, Fife KY16 9ST, United Kingdom

## Abstract

We report the synthesis of 2-indolylboramides via a palladium (Pd)-catalyzed heteroannulation of 2-iodoaniline derivatives and Bdan-capped alkynes (Bdan, 1,8-diaminonaphthalene boronamide). The process is highly regioselective, affording a diverse range of C2-borylated indoles. The synthetic utility of the process is demonstrated through the concise synthesis of the *Tetradium* alkaloid skeleton.

## Graphical abstract

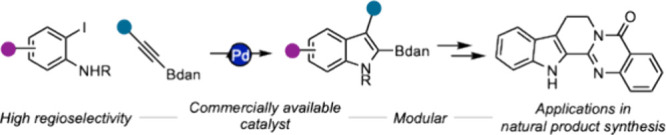

The indole skeleton represents a key structural motif in organic chemistry due to its prevalence in both natural products and biologically active compounds (Figure 1a).^1^ As a result, the synthesis and functionalization of these scaffolds has received extensive attention from the synthetic chemistry community.^2^ Despite this focus, methods that allow for the synthesis of borylated indoles have received surprisingly low attention. Classical approaches to the synthesis of such compounds have largely relied on the functionalization of preassembled indole scaffolds via borylation strategies such as stoichiometric metalation/borylation,^3^ electrophilic borylation,^4^ and metal-mediated C–H borylation.^5^

**Figure 1:**
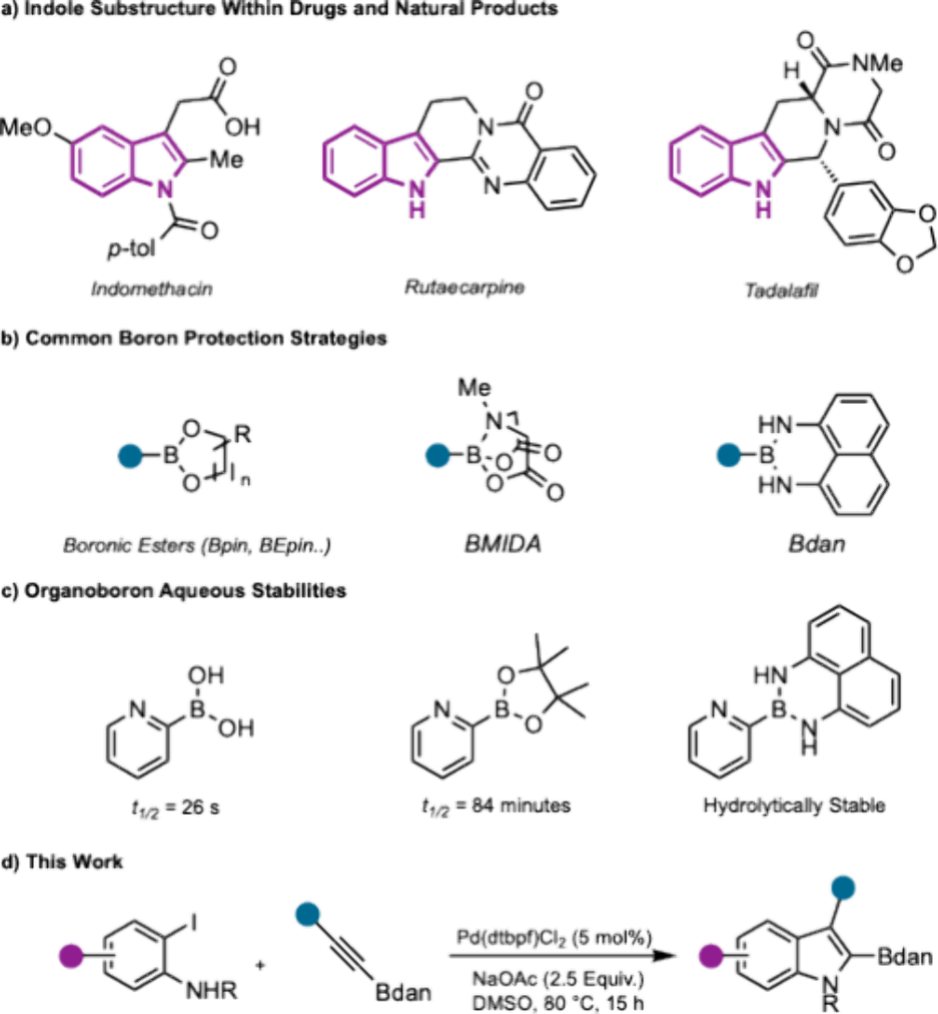
(a) Indoles in natural products and pharmaceuticals. (b) Protected boronic acid derivatives. (c) Organoboron hydrolytic stability. (d) This work: synthesis of C2-Bdan indoles.

The synthesis of borylated indoles is of synthetic interest due to the variety of potential downstream applications. In particular, organoborons are well-established as the nucleophilic component of the Suzuki-Miyaura cross-coupling, one of the principal methods employed to form carbon–carbon bonds in modern synthetic chemistry.^6^ While the Lewis acidic nature of the boron center facilitates mild activation to afford the corresponding boronate, it also renders many boronic acids and esters prone to hydrolytic protodeborylation.^7^ To circumvent this instability, a range of protecting group strategies have been developed. These typically rely on attenuating Lewis acidity at boron either by a change in hybridization,^8^ (BMIDA, MIDA = *N*-methyliminodiacetoxy), or by increased electron donation into the vacant p-orbital at boron by adjacent nitrogen atoms (Bdan, dan = 1,8-diaminonaphthalene) (Figure 1b).^9^ The increase in hydrolytic stability afforded by Bdan derivatives is exemplified by comparison between solution state lifetimes of 2- pyridinylboronic acid and the analogous 2-pyridinyl-Bdan. While the unprotected boronic acid undergoes rapid hydrolysis, the boramide shows comparatively negligible levels of protodeborylation (Figure 1c).^7,10^

The diminished electrophilicity of aryl Bdans has implications in cross-coupling reactions. For example, the relative stability of the Bdan group permits chemoselective cross-coupling of aryl boronic acids in the presence of aryl Bdans, which can subsequently be ‘unmasked’ and coupled, enabling iterative Suzuki-Miyaura approaches to oligoarenes, as reported by Suginome and co-workers.^9^

As concurrently reported by Tsuchimoto and Saito, formation and subsequent direct cross-coupling of Bdan-ate complexes requires, in most cases, alkoxide bases to be used as opposed to the carbonate bases more typically employed in Suzuki-Miyaura cross-couplings.^11^ Recently, Yoshida disclosed the synthesis of C2-Bdan indoles via a Sonogashira/Cacchi cascade.^12^ Based on our previous work,^13^ we reasoned that a Larock heteroannulation between Bdan-capped alkynes and 2-iodoanilines would also provide straightforward access to C2-Bdan indoles, while additionally permitting substitution at the C3 site (Figure 1d). Herein, we report the development of this process and demonstrate its applicability in the total synthesis of complex alkaloid products.

From the outset, we were conscious of the differing reactivities of anilines and anilides in Larock heteroannulations^13^ and as such chose to examine the reaction of both **1a** and **2a** with **3a** through the course of our optimization studies. Following optimization, reaction conditions that enabled the synthesis of **4a** from **1a** in high yield and achieving complete regioselectivity were identified (**Entry 1**), with selected optimization data shown in Table 1 (for full details, see the Supporting Information). Di-*tert*-butylphosphinoferrocene (DTBPF) was found to be optimal, with the related diphenylphosphinoferrocene (DPPF) complex significantly less effective (**Entry 2**).

**Table 1:**
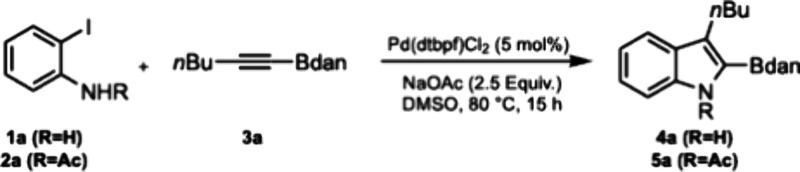
Reaction Development

entry	*R*	deviation from ‘standard conditions’	yield (%)^a^
1	H	none	99
2	H	Pd(dppf)Cl_2_ (5 mol %)	64
3	H	Pd(OAc)_2_ (10 mol %) + dppf (10 mol %)	18
4	H	room temperature	<5
5	Ac	none	13%
6	Ac	Pd(OAc)_2_ (5 mol %)	10%
7	Ac	Pd(OAc)_2_ (5 mol %), LiCl (2.0 equiv), DMF, 65 °C	97%

a Yields calculated by ^1^H NMR analysis of the crude reaction mixture using trichloroethylene as an internal standard.

The necessity for chloride sources within Larock type annulations is well-established,^14^ with (super)­stoichiometric quantities of exogenous chloride often required to achieve good levels of conversion.^15^ In the case of **1a**, the need for (super)­stoichiometric amounts of chloride salts was mitigated by the catalytic quantities of chloride ions provided by the Pd precatalyst. Examining chloride-free Pd sources resulted in a considerable decrease in conversion (**Entry 3**). Lower reaction temperatures were not tolerated (**Entry 4**). It should be noted that attempting the heteroannulation with 2-bromoaniline resulted in a significant decrease in conversion, affording **4a** in only 19% yield. This is consistent with Reisman’s proposal that heteroannulation of such substrates is most efficiently catalyzed by Pd(0)-monophosphine complexes,^16^ with significantly more forcing conditions typically employed for Pd­(II) catalysts bearing bidentate phosphine ligands.^17^

When **2a** was subjected to the same conditions, the efficiency of the reaction was significantly diminished (**Entry 5**), aligning with our previous observations on related systems.^13^ Screening alternative Pd sources did not present a solution to this limitation, with chloride-free Pd sources affording even lower amounts of product (**Entry 6**). This issue could be overcome by using more typically employed Larock conditions^18^ with superstoichiometric quantities of exogenous chloride, furnishing **5a** in near quantitative yield (**Entry 7**).

The generality of both methods was assessed, beginning with aniline derivatives (Scheme 1a). A range of 4-substituted 2-iodoanilines were employed, affording the corresponding borylated indoles (**4a–4f**) in moderate to excellent yields. Substitution at the 3-position of the aniline had a detrimental effect on yield, with **4h** isolated in a modest 34% yield. Conversely, more remote substitution was well tolerated, with 5-chloro-2-iodoaniline undergoing facile heteroannulation to afford **4i** in 69% yield. Varying the nature of the alkyne substituent provided access to C3-alkyl indoles **4j** and **4k**. It should be noted that while the presence of free heteroatoms resulted in some diminished reactivity, (for full details, see the Supporting Information) protected derivatives were readily tolerated, affording benzyl ether **4l** and silyl ethers **4m** and **4n**. Borylated fused heterocyclic systems could be accessed using this methodology, with 7-azaindole **4o** isolated in 38% yield and regioselectivity unambiguously confirmed by SCXRD. Scaffolds of this type find frequent application as hinge binders as well as highly potent kinase inhibitors, further demonstrating the potential applications of this methodology.^19^ It is notable that in all cases, despite the general instability of the Bdan group toward acidic conditions, the products could be isolated using conventional silica gel flash chromatography, without requiring Florisil or pretreated silica gel.

**Scheme 1:**
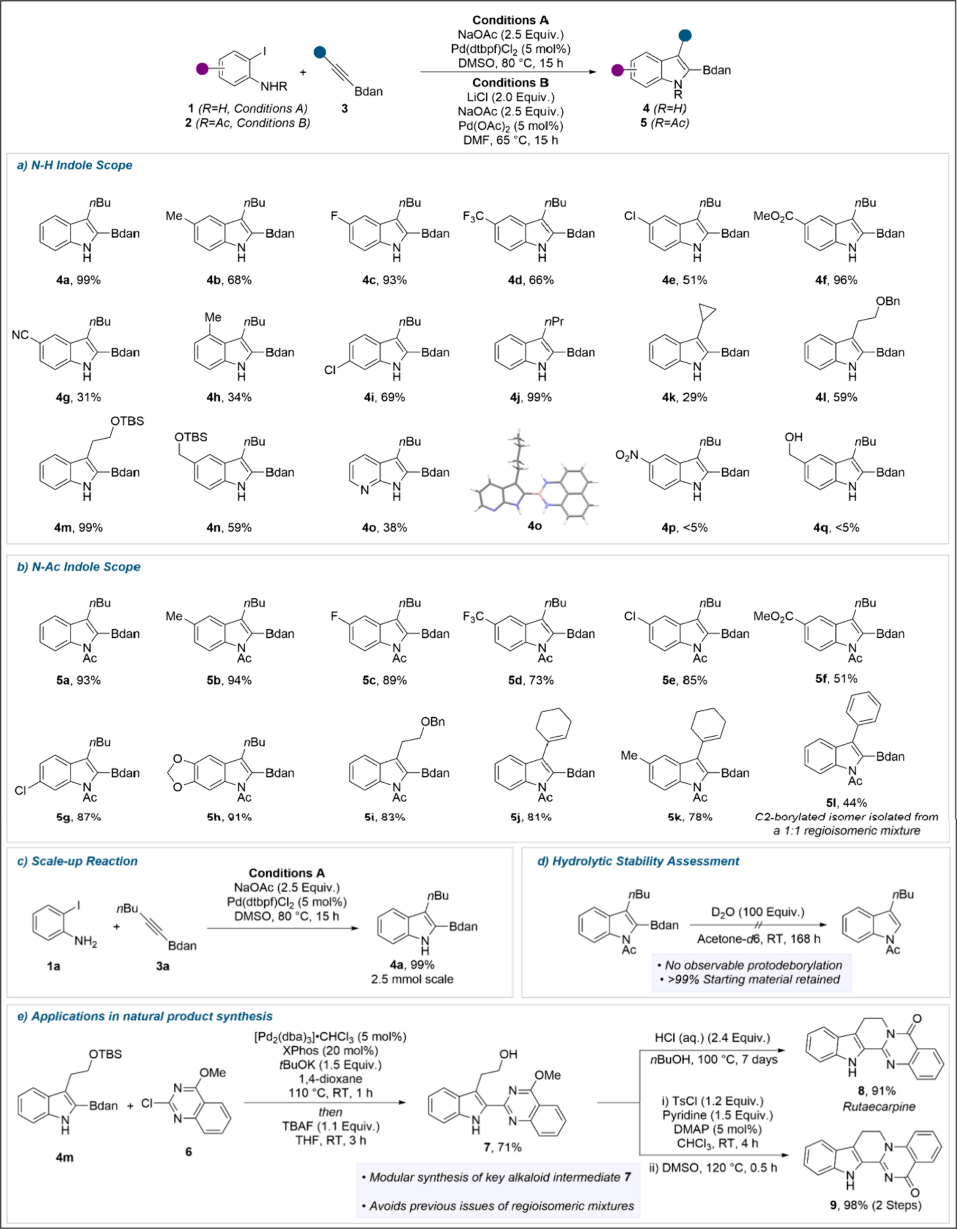
^a^

a Reactions were conducted as a single run. (a) Conducted under Conditions A. Reactions performed on a 0.2 mmol scale. Isolated yields. Products were isolated as a single regioisomer. (b) Conducted under Conditions B. Reactions performed on a 0.2 mmol scale. Isolated yields. Products were isolated as a single regioisomer. (c) Isolated yield. (d) Degradation monitored and quantified by ^1^H NMR analysis using trichloroethylene as an internal standard. (e) Isolated yields.

The scope of 2-iodoacetanilides amenable to heteroannulation was next evaluated under the modified conditions detailed in Table 1. As with the aniline scope, a range of 4-substituted 2-iodoanilides were converted to the corresponding indoles **5a–5f** in largely excellent yields and with complete regioselectivity (Scheme 1b). The nature of the alkyne could again be varied to incorporate protected heteroatoms, affording benzyl ether **5i** in 83% yield. Bdan-capped enynes also proved excellent substrates, providing access to indolyl dienes **5j** and **5k**. The application of aryl alkynes in this reaction resulted in a diminished yield, with 5l isolated in 44% yield. This was attributed to a significant decrease in regioselectivity for these substrates, with the C2 and C3 borylated indoles formed in an approximately 1:1 ratio. Given that that electronic effects in Larock heteroannulations are rarely sufficient to account for such variance in regioselectivity,^20^ the origin of this decrease is attributed to the increased steric profile of the aryl substituent. Attempted annulation between the same alkyne and **1a** was similarly unsuccessful, with deborylative-coupling products instead isolated. It should be noted that the methodology was incompatible with sulfonamide or carbamate protected 2-iodoaniline derivatives, in agreement with our previous observations.^13^

The reaction proved to be readily scalable, with the heteroannulation of **1a** and **3a** providing indole **4a** on multimmol scale (Scheme 1c) with no decrease in yield and regioselectivity relative to the 0.2 mmol scale reaction shown in Scheme 1a. As a key metric of boronamide synthetic utility, we assessed the hydrolytic stability of the indole products generated. As previously reported with alkyl Bdan derivatives,^21^ **5a** demonstrated excellent hydrolytic stability, with negligible degradation over a prolonged period (Scheme 1d).

Finally, in order to demonstrate the broader synthetic utility of the process, we sought to use the generated indolyl boramides as intermediates in the synthesis of complex indole-containing products. We selected rutaecarpine **8**, a complex alkaloid with an array of protective cardiovascular properties,^22^ and isomeric derivative **9** as targets, with **7** identified as a key intermediate based on earlier work by Pan and Bannister.^23^ Previously, **7** had been accessed by sequential Sonogashira and Larock reactions, although the poor regioselectivity of the heteroannulation in this case had necessitated chromatographic separation of the C2 and C3 arylated indoles. Given the complete regioselectivity of the heteroannulation developed herein an operationally simpler synthetic sequence was developed (Scheme 1e). Cross-coupling partner, chloroquinazoline **6**, was readily prepared via S_N_Ar between 2,4-dichloroquinazoline and NaOMe. Using the conditions previously identified by Saito and co-workers,^11a^ indole **4m** underwent facile cross-coupling to afford the corresponding biaryl, which was subsequently treated with TBAF to afford alcohol **7** in 71% yield over the two steps. From **7**, access to **8** and **9** could be achieved using previously reported cyclization procedures.^23^

In summary, a highly regioselective procedure for the synthesis of C2-Bdan indoles has been developed via heteroannulation of 2-iodoaniline derivatives and Bdan-capped alkynes under palladium catalysis. The reaction is tolerant of a range of functionalities, affording a diverse array of substituted indoles. The products display high hydrolytic stability consistent with the boronamide class. The utility of the procedure was demonstrated through the concise synthesis of complex alkaloid products.

## Supplementary Material



## Data Availability

The data underlying this study are available in the published article and its online Supporting Information.

## Notes

Experimental procedures, procedures, crystal structure data and NMR spectra (PDF)
The research data supporting this publication can be accessed at https://doi.org/10.17630/361d686b-28d3-421e-8886-6571e53924d7.

## Notes

Dean D. Roberts - EaStCHEM, School of Chemistry, University of St Andrews, North Haugh, St Andrews, Fife KY16 9ST, United Kingdom

## Notes

**John M. Halford-McGuff-** *EaStCHEM, School of Chemistry, University of St Andrews, North Haugh, St Andrews, Fife KY16 9ST, United Kingdom.*

## Notes

**Aidan P. McKay-** *EaStCHEM, School of Chemistry, University of St Andrews, North Haugh, St Andrews, Fife KY16 9ST, United Kingdom.*
