# Supplementary material for: Palladium-Catalyzed Heteroannulation of Bdan-Capped Alkynes: Rapid Access to Complex Indole Scaffolds
Source: J Org Chem. 2025 Sep 15;90(38):13779–83. doi: 10.1021/acs.joc.5c01781 (PMC12481573; doi:10.1021/acs.joc.5c01781)

Supporting Information

Palladium-Catalyzed Heteroannulation of Bdan-Capped Alkynes: Rapid Access to Complex Indole Scaffolds

Dean D. Roberts, John M. Halford-McGuff, Marek Varga, Aidan P. McKay, and Allan J. B. Watson*
EaStCHEM, School of Chemistry, University of St Andrews, North Haugh, St Andrews, Fife, KY16 9ST, UK
*Email: aw260@st-andrews.ac.uk

Contents:

1.	General Experimental Details	S2
2.	General Procedures	S3
3.	Reaction Optimization	S5
4.	Starting Material Synthesis	S9
5.	Characterization Data	S18
6.	Scale-up Reaction	S45
7.	Alkaloid Synthesis	S45
8.	X-ray Crystallography	S48
9.	Bibliography	S49

1. General Experimental Details

1.1 Purification of Solvents and Reagents

Reagents were obtained from commercial suppliers and were not purified further unless specified. Anhydrous THF was provided by a PureSolv SPS-400-5 solvent purification system. Anhydrous DMSO was obtained commercially (Thermo Scientific). All other anhydrous solvents were obtained by storing the requisite solvent over activated 4 Å molecular sieves, under N_2, for 24 hours.

1.2 Experimental Details

Reactions were carried out using conventional glassware or in capped 10 mL microwave vials. Glassware was either flame-dried under vacuum or allowed to dry in a 180 °C oven for 24 hours before use and then sparged with N_2. Reactions carried out at elevated temperatures were performed using a sand bath (sand temperature) or oil bath (oil temperature) atop a temperature-regulated hotplate/stirrer. Room temperature was approximately 20 °C. Cooling to 0 °C was achieved using an ice/water bath. Cooling to −84 °C was achieved using a liquid N_2/EtOAc bath. Water or oxygen-sensitive reactions were carried out in flame- or oven-dried glassware under inert atmosphere (N_2) using standard vacuum lines techniques.

1.3 Purification of Products

Thin layer chromatography was performed using Merck silica plates coated with fluorescent indicator UV254. These were analysed under 254 nm UV light and/or developed using aqueous potassium permanganate or ethanolic vanillin solution. Flash column chromatography was performed using silica gel (40–62 µm, Fluorochem).
Note: Purification of Bdan compounds on silica gel was not found to result in protodeborylation.

1.4 Analysis of Products

^{1}H, ^{13}C, ^{19}F NMR spectra were recorded by either a Bruker AVII 400 (BBFO probe), Bruker AV 400 (BBFO probe), Bruker AVIII 500 (Prodigy BBFO probe), Bruker AVIII-HD 500 (BBFO+ probe) or Bruker AVIII-HD 700 (Prodigy TCI probe) at 400-101-376 MHz, 500-126-377 MHZ, or 700-176 MHz, respectively. ^{11}B NMR spectra were recorded on a Bruker AV 400 spectrometer at 128 MHz. All spectra were recorded at room temperature with the deuterated solvents used as a lock for spectra and internal reference (*d*-chloroform: ^{1}H, 7.26 ppm; ^{13}C, 77.16 ppm), (*d*-DMSO, ^{1}H 2.50 ppm; ^{13}C, 39.52, central peak), (*d*-acetonitrile: ^{1}H 1.94 ppm, ^{13}C 1.30 ppm). Unless otherwise stated, spectra were proton-decoupled. For ^{11}B NMR, samples were run using a standard borosilicate tube and the spectra baselines corrected during processing. All ^{11}B NMR spectra were externally referenced to $F_3B{\bullet}OEt_2$ in $CDCl_3$. All chemical shifts (δ) are reported in parts per million (ppm). All coupling constants, *J*, are quoted in Hz. NMR spectra are reported as follows: chemical shift/ppm (multiplicity, coupling constant(s), number of nuclei). Multiplicity given as br (broad), s (singlet), d (doublet), t (triplet), q (quartet), quint (quintet), h (hextet), m (multiplet), and combinations thereof. Signals which overlap with one another are described as multiplets. Fourier transformed infra-red (FTIR) spectra were obtained using a Shimadzu IRAffinity-1 Fourier transform IR spectrophotometer with a Specac Aquest ATR (diamond puck). Spectra were recorded as specified within the procedure as solids, oils, or thin films (CH_2Cl_2 or acetone). Transmittance is recorded as maximal absorption in wavenumbers (cm^{-1}). High resolution mass spectrometry (HRMS) was recorded on a Bruker micrOTOF benchtop ESI with either positive or negative electrospray ionisation or EI using a Thermo Mat 900XP, Double Focussing Hi-resolution mass spectrometer at the University of Edinburgh mass spectrometry facility (SIRCAMS). The number of decimal places is determined by the accuracy of the machine.

2. General Procedures

General Procedure A: Synthesis of *N*-(2-iodophenyl)acetamides

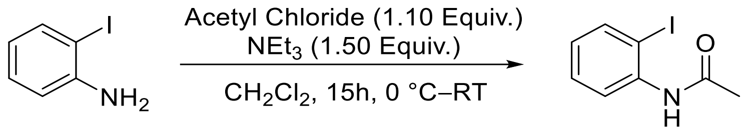

An oven-dried flask was charged with the requisite 2-iodoaniline (1.00 Equiv.), sealed and purged with N_2. Anhydrous CH_2Cl_2 (0.5 M) was then added followed by NEt_3 (1.50 Equiv.) and the mixture was cooled to 0 ˚C. Acetyl chloride (1.10 Equiv.) was then added dropwise, before the mixture was allowed to warm to room temperature and stirred at the same temperature for 15 hours. The reaction was quenched *via* addition of sat. aq. NH_4Cl (5.0 mL/mmol aniline) and the organic phase collected. The aqueous phase was then extracted twice with CH_2Cl_2 (5.00 mL/mmol aniline.) The combined organic extracts were then washed with brine (5.0 mL/mmol aniline) and dried over Na_2SO_4 before being filtered and concentrated under reduced pressure. The crude product was then purified *via* flash column chromatography (silica gel, *See below for individual solvent systems*).

General Procedure B: Sonogashira Coupling

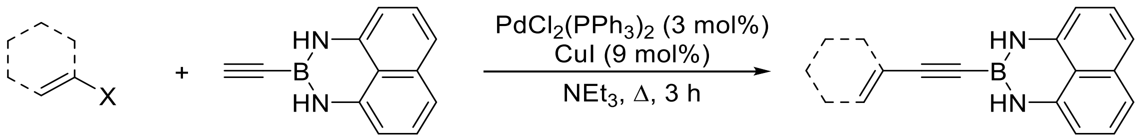

A flame-dried Schlenk flask was charged with CuI (9 mol%), $PdCl_2(PPh_3)_2$ (3 mol%) and the requisite halide or triflate (1.50 Equiv.). The flask was sealed, evacuated, and backfilled with N_2 three times before NEt_3 (0.5 M, purged with N_2 for 10 minutes prior to use) and alkyne (1.00 Equiv.) were sequentially added under a positive flow of N_2. The reaction was heated to the specified temperature (*see below for details*) in an oil bath and stirred for 3 hours at the same temperature. The reaction mixture was then diluted with EtOAc (10.0 mL), filtered through celite, and concentrated under reduced pressure. The crude product was then purified *via* flash column chromatography (silica gel, *see below for individual solvent systems*).

General Procedure C: Reaction Optimisation

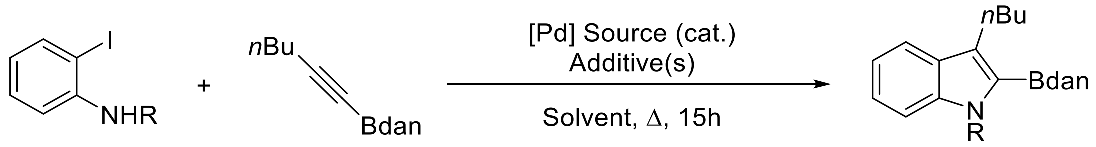

(R=H, Ac) ***(R=H, Ac)***

An oven-dried microwave vial was charged with the specified palladium source, 2-(hex-1-yn-1-yl)-2,3-dihydro-1H-naphtho[1,8-de][1,3,2]diazaborinine (24.8 mg, 0.050 mmol) and the specified amount of either 2-iodoaniline or *N*-2-(iodophenyl)acetamide alongside any additives employed in individual experiments. The vial was sealed, evacuated and backfilled with N_2 three times. Anhydrous solvent (0.1 M) was added, and the mixture was heated to the specified temperature in a sand bath and stirred for 15 hours at the same temperature. After this time, the reaction mixture was allowed to cool to room temperature before 10% aq. LiCl (5.0 mL) and

EtOAc (5.0 mL) were added and the organic phase extracted. The aqueous phase was then extracted twice with EtOAc (5.0 mL). The combined organic extracts were washed with 10% aq. LiCl (10.0 mL) and dried over Na_2SO_4 before being filtered and concentrated under reduced pressure. The resultant residue was then dissolved in $CDCl_3$ and an equimolar amount of trichloroethylene relative to the Bdan-capped alkyne was added before ^{1}H NMR analysis.

General Procedure D: Synthesis of 2-Bdan indoles (Anilines)

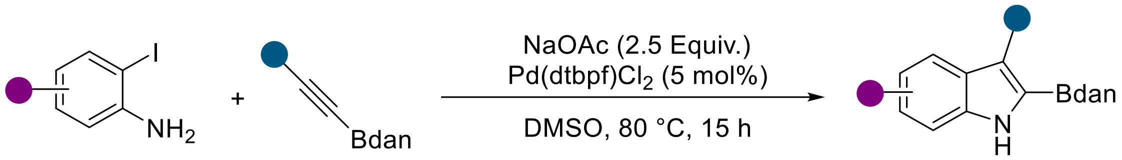

An oven-dried microwave vial was charged with the requisite alkyne (0.40 mmol, 2.00 Equiv.), Pd(dtbpf)Cl_2 (6.5 mg, 10 µmol, 5.0 mol%), 2-iodoaniline derivative (0.20 mmol, 1.00 Equiv., *added at this stage if solid*) and NaOAc (41 mg, 0.5 mmol, 2.50 Equiv.). The vial was sealed and evacuated and backfilled with N_2 three times. Anhydrous DMSO (2.00 mL, 0.10 M) was added (*note − if the aniline derivative is a liquid, it was added at this stage as a solution in DMSO*) and the mixture was heated to 80 ºC in a sand bath and stirred for 15 hours at the same temperature. After this time, the reaction mixture was allowed to cool to room temperature before 10% aq. LiCl (10.00 mL) and EtOAc (10.0 mL) were added and the organic phase extracted. The aqueous phase was then extracted twice with EtOAc (10.00 mL). The combined organic extracts were washed with 10% aq. LiCl (20.0 mL) and dried over Na_2SO_4 before being filtered and concentrated under reduced pressure. The crude product was then purified *via* flash column chromatography (silica gel, *see below for individual solvent systems*).

General Procedure E: Synthesis of 2-Bdan indoles (Anilides)

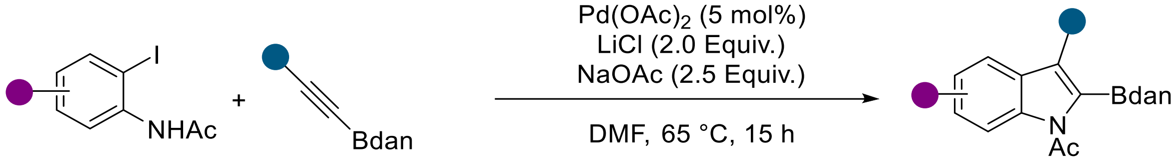

An oven-dried microwave vial was charged with the requisite alkyne (0.20 mmol, 1.00 Equiv.), Pd(OAc)$_2$ (2.2 mg, 10 µmol, 5.0 mol%), *N*-(2-iodophenyl)-anilide derivative (0.24 mmol, 1.20 Equiv., *added at this stage if solid*), LiCl (16.8 mg, 0.400 mmol, 2.00 Equiv.) and NaOAc (41 mg, 0.50 mmol, 2.5 Equiv.). The vial was sealed and evacuated and backfilled with N_2 three times. Anhydrous DMF (2.00 mL, 0.10 M) was added (*note − if N-(2-iodophenyl)-anilide derivative is a liquid, it was added at this stage as a solution in DMF*) and the mixture was heated to 65 ºC in a sand bath and stirred for 15 hours at the same temperature. After this time, the reaction mixture was allowed to cool to room temperature before 10% aq. LiCl (10.0 mL) and EtOAc (10.0 mL) were added and the organic phase extracted. The aqueous phase was then extracted twice with EtOAc (10.0 mL). The combined organic extracts were washed with 10% aq. LiCl (20.0 mL) and dried over Na_2SO_4 before being filtered and concentrated under reduced pressure. The crude product was then purified *via* flash column chromatography (silica gel, *see below for individual solvent systems*).

3. Reaction Optimization

3.1 Aniline Optimization Data

Catalyst Screen

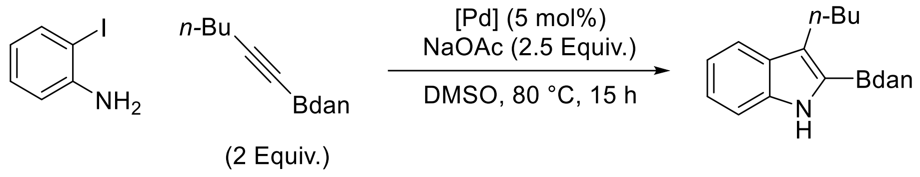

Entry	[Pd]	Yield (*rr*)[a]
1	Pd(dppf)Cl_2	64% (>20:1)
2	$Pd(OAc)_2$ + dppf (10 mol%)	18% (>20:1)
3	$PdCl_2$ + dppf (10 mol%)	23% (>20:1)
4	[Pd(allyl)XPhos]Cl	49% (>20:1)
5	XPhosPdG2	45% (>20:1)
6	SPhosPdG2	55% (>20:1)
7	[Pd(allyl)SIPr	24% (>20:1)
8	XantPhosPdG4	15% (>20:1)
**9**	**Pd(dtbpf)Cl_2**	**86% (>20:1)**
10	$Pd(PPh_3)_2Cl_2$	41% (>20:1)

[a]NMR yield and regioisomeric ratio (*rr)* determined through ^{1}H NMR analysis of the crude reaction mixture using trichloroethylene as an internal standard.

Solvent Screen

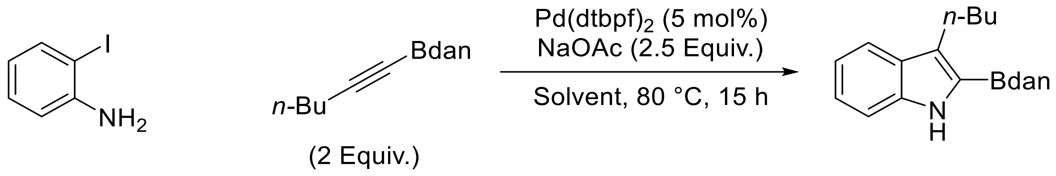

Entry	Solvent	Yield (*rr*)[a]
**1**	**DMSO**	**86% (>20:1)**
2	*t*-BuOH	N.R
3	MeCN	39% (17:22)
4	DCE	19% (9:11)
5	THF	N.R
6	PhMe	N.R

[a]NMR yield and regioisomeric ratio (*rr)* determined through ^{1}H NMR analysis of the crude reaction mixture using trichloroethylene as an internal standard.

Temperature Screen

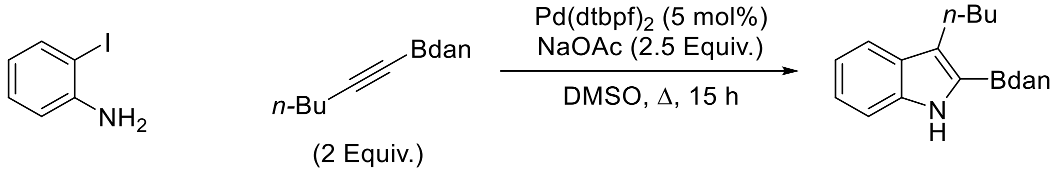

Entry	Temperature (°C)	Yield (*rr*)[a]
1	20	N.R.
2	60	37% (>20:1)
**3**	**80**	**86% (>20:1)**
4	100	42% (>20:1)

[a]NMR yield and regioisomeric ratio (*rr)* determined through ^{1}H NMR analysis of the crude reaction mixture using trichloroethylene as an internal standard.

Stoichiometry Screen

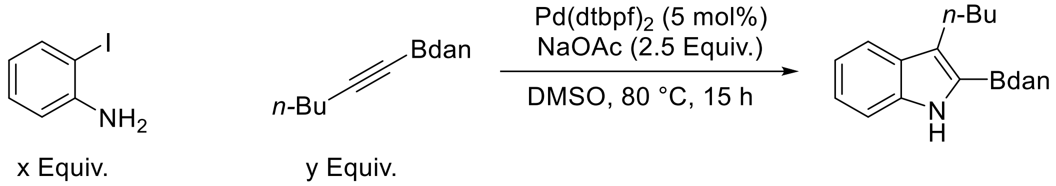

Entry	x	y	Yield (*rr*)[a]
1	1.2	1	86% (>20:1)
2	1.5	1	84% (>20:1)
3	2.0	1	65% (>20:1)
4	1	1.5	85% (>20:1)
**5**	**1**	**2.0**	**>99% (>20:1)**

[a]NMR yield and regioisomeric ratio (*rr)* determined through ^{1}H NMR analysis of the crude reaction mixture using trichloroethylene as an internal standard.

3.2 Anilide Optimization Data

Catalyst Screen

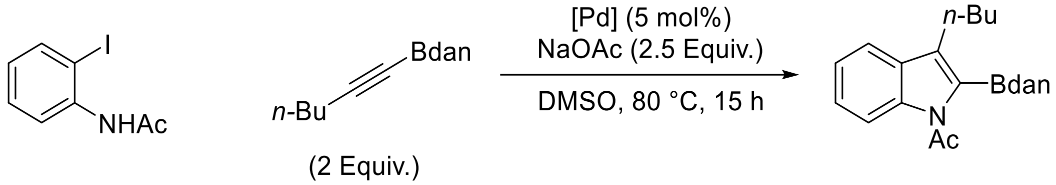

Entry	[Pd]	Yield (*rr*)[a]
1	Pd(dppf)Cl_2	13% (>20:1)
2	$Pd(OAc)_2$ + dppf (10 mol%)	12% (>20:1)
**3**	**$Pd(OAc)_2$**	**10% (>20:1)**
4	$PdCl_2$	10% (>20:1)

[a]NMR yield and regioisomeric ratio (*rr)* determined through ^{1}H NMR analysis of the crude reaction mixture using trichloroethylene as an internal standard.

Note: While Entry 3 gave marginally lower conversion than the reactions containing phosphine ligands, subsequent additive experiments showed the presence of phosphine ligands was not required for high selectivity and conversion.

Additive Screen

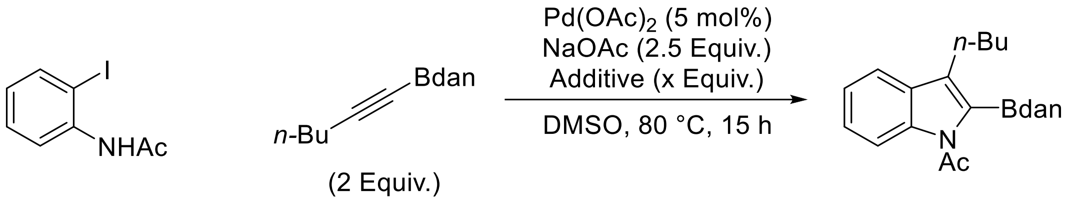

Entry	Additive (Equiv.)	Yield (*rr*)[a]
1	NaCl (1.00)	39% (>20:1)
2	LiCl (1.00)	54% (>20:1)
**3**	**LiCl (2.00)**	**89% (>20:1)**
4	TBAC (1.00)	25% (>20:1)
5	TBAC·H_2O (1.00)	19% (>20:1)
6	LiOAc·$2H_2O$ (1.00)	20 % (>20:1)

[a]NMR yield and regioisomeric ratio (*rr)* determined through ^{1}H NMR analysis of the crude reaction mixture using trichloroethylene as an internal standard.

Solvent Screen

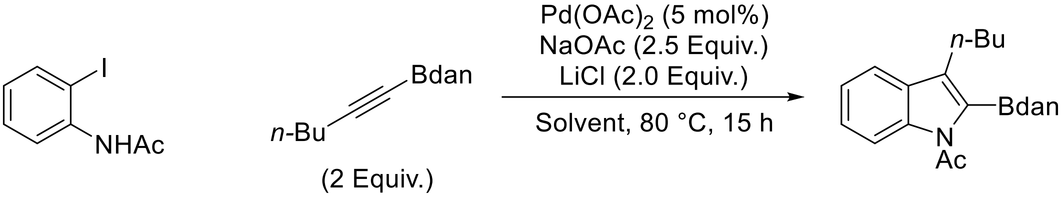

Entry	Solvent	Yield (*rr*)[a]
**1**	**DMF**	**97% (>20:1)**
2	DMSO	89% (>20:1)
3	MeCN	31% (15:1)
4	THF	N.R
5	PhMe	N.R

[a]NMR yield and regioisomeric ratio (*rr)* determined through ^{1}H NMR analysis of the crude reaction mixture using trichloroethylene as an internal standard.

Stoichiometry Screen

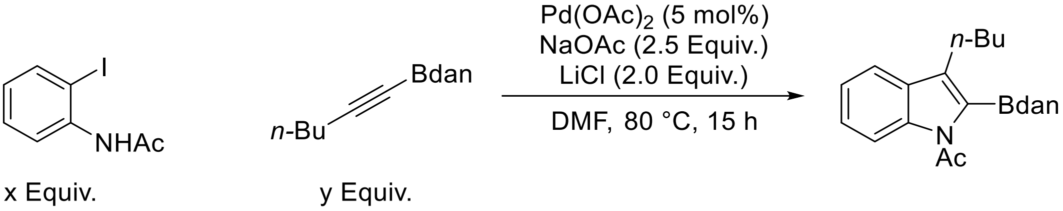

Entry	x	y	Yield (*rr*)[a]
1	1.0	2.0	97% (>20:1)
2	1.0	1.5	96% (>20:1)
3	1.0	1.0	91% (>20:1)
**4**	**1.2**	**1.0**	**96% (>20:1)**

[a]NMR yield and regioisomeric ratio (*rr)* determined through ^{1}H NMR analysis of the crude reaction mixture using trichloroethylene as an internal standard.

Temperature Screen

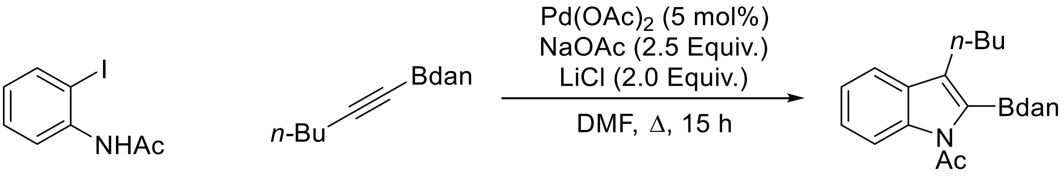

Entry	Temperature (°C)	Yield (*rr*)[a]
1	RT	<5% (n.d.)
2	40	41% (>20:1)
**3**	**65**	**97% (>20:1)**

[a]NMR yield and regioisomeric ratio (*rr)* determined through ^{1}H NMR analysis of the crude reaction mixture using trichloroethylene as an internal standard.

4. Starting Material Synthesis

4.1 *N*-(2-Iodophenyl)Acetamide Synthesis

***N*-(2-iodophenyl)acetamide (2a)**

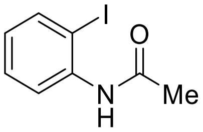

Prepared according to General procedure A using 2-iodoaniline (440 mg, 2.00 mmol, 1.00 Equiv.), acetyl chloride (155 µL, 2.20 mmol, 1.10 Equiv.) and triethylamine (420 µL, 3.00 mmol, 1.50 Equiv.). Following workup, the product was purified by flash column chromatography (silica gel, 0 to 5% EtOAc in hexane) to afford the title compound as a white solid (494 mg, 94%). Spectral data is in agreement with literature reports.[1]

^{1}H NMR (400 MHz, $CDCl_3$) δ 8.20 (d, *J* = 8.7 Hz, 1H), 7.78 (dd, *J* = 7.9, 1.4 Hz, 1H), 7.41 (br s, 1H), 7.34 (t, *J* = 8.2 Hz, 1H), 6.84 (t, *J* = 8.0 Hz, 1H), 2.24 (s, 3H).

***N*-(2-iodo-4-methylphenyl)acetamide (2b)**

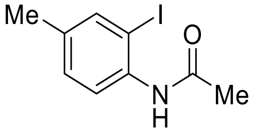

Prepared according to General procedure A using 4-methyl-2-iodoaniline (466 mg, 2.00 mmol, 1.00 Equiv.), acetyl chloride (155 µL, 2.20 mmol, 1.10 Equiv.) and triethylamine (420 µL, 3.00 mmol, 1.50 Equiv.). Following workup, the product was purified by flash column chromatography (silica gel, 0 to 5% EtOAc in hexane) to afford the title compound as a white solid (529 mg, 96%). Spectral data is in agreement with literature reports.[1]

^{1}H NMR (400 MHz, $CDCl_3$) δ 8.03 (d, *J* = 8.3 Hz, 1H), 7.61 (s, 1H), 7.31 (br s, 1H), 7.15 (d, *J* = 7.9 Hz, 1H), 2.28 (s, 3H), 2.22 (s, 3H).

***N*-(2-iodo-4-fluorophenyl)acetamide (2c)**

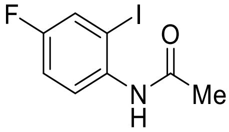

Prepared according to General procedure A using 4-fluoro-2-iodoaniline (474 mg, 2.00 mmol, 1.00 Equiv.), acetyl chloride (155 µL, 2.20 mmol, 1.10 Equiv.) and triethylamine (420 µL, 3.00 mmol, 1.50 Equiv.). Following workup, the product was purified by flash column chromatography (silica gel, 0 to 5% EtOAc in hexane) to afford the title compound as a white solid (498 mg, 89%). Spectral data is in agreement with literature reports.[1]

^{1}H NMR (400 MHz, $CDCl_3$) δ 8.11 (dd, *J* = 9.1, 5.5 Hz, 1H), 7.50 (dd, *J* = 7.7, 2.9 Hz, 1H), 7.29 (br s, 1H), 7.09 (ddd, *J* = 9.1, 7.8, 2.9 Hz, 1H), 2.23 (s, 3H).

***N*-(2-iodo-4-trifluoromethylphenyl)acetamide (2d)**

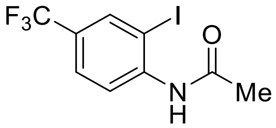

Prepared according to General procedure A using 4-trifluoromethyl-2-iodoaniline (573 mg, 2.00 mmol, 1.00 Equiv.), acetyl chloride (155 µL, 2.20 mmol, 1.10 Equiv.) and triethylamine (420 µL, 3.00 mmol, 1.50 Equiv.). Following workup, the product was isolated purified by flash column chromatography (silica gel, 0 to 5% EtOAc in hexane) to afford the title compound as an off-white solid (554 mg, 84%). Spectral data is in agreement with literature reports.[2]

^{1}H NMR (400 MHz, $CDCl_3$) δ 8.43 (d, J = 8.6 Hz, 1H), 8.07 – 7.97 (s, 1H), 7.71 – 7.55 (m, 2H), 2.30 (s, 3H).

***N*-(2-iodo-4-chlorophenyl)acetamide (2e)**

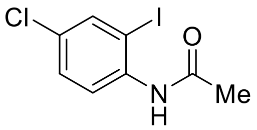

Prepared according to General procedure A using 4-chloro-2-iodoaniline (506 mg, 2.00 mmol, 1.00 Equiv.), acetyl chloride (155 µL, 2.20 mmol, 1.10 Equiv.) and triethylamine (420 µL, 3.00 mmol, 1.50 Equiv.). Following workup, the product was purified by flash column chromatography (silica gel, 0 to 5% EtOAc in hexane) to afford the title compound as a white solid (549 mg, 93%). Spectral data is in agreement with literature reports.[3]

^{1}H NMR (400 MHz, $CDCl_3$) δ 8.15 (d, J = 8.8 Hz, 1H), 7.75 (s, 1H), 7.39 (br s, 1H), 7.32 (d, J = 8.7 Hz, 1H), 2.24 (s, 3H).

Methyl 4-acetamido-3-iodo-benzoate (2f)

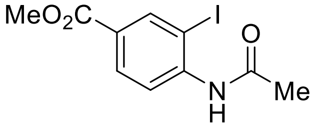

Prepared according to General procedure A using methyl-4-amino-3-iodobenzoate (555 mg, 2.00 mmol, 1.00 Equiv.), acetyl chloride (155 µL, 2.20 mmol, 1.00 Equiv.) and triethylamine (420 µL, 3.00 mmol, 1.50 Equiv.). Following workup, the product was purified by flash column chromatography (silica gel, 0 to 10% EtOAc in hexane) to afford the title compound as an off-white solid (524 mg, 82%). Spectral data is in agreement with literature reports.[3]

^{1}H NMR (400 MHz, $CDCl_3$) δ 8.45 (d, J = 1.9 Hz, 1H), 8.38 (d, J = 8.7 Hz, 1H), 8.00 (dd, J = 8.7, 2.0 Hz, 1H), 7.62 (br s, 1H), 3.90 (s, 3H), 2.27 (s, 3H).

***N*-(2-iodo-5-chlorophenyl)acetamide (2g)**

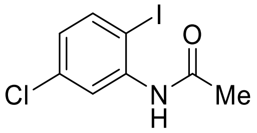

Prepared according to General procedure A using 5-chloro-2-iodoaniline (506 mg, 2.00 mmol, 1.00 Equiv.), acetyl chloride (155 µL, 2.20 mmol, 1.10 Equiv.) and triethylamine (420 µL, 3.00

mmol, 1.50 Equiv.). Following workup, the product was isolated purified by flash column chromatography (silica gel, 0 to 10% EtOAc in hexane) to afford the title compound as a white solid (526 mg, 89%). Spectral data is in agreement with literature reports.[3]

^{1}H NMR (400 MHz, $CDCl_3$) δ 8.34 (s, 1H), 7.69 (d, J = 8.5 Hz, 1H), 7.44 (br s, 1H), 6.87 (dd, J = 8.4, 2.5 Hz, 1H), 2.27 (s, 3H).

N-(6-iodo-1,3-benzodioxol-5-yl)acetamide (2h)

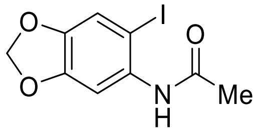

Prepared according to previously reported procedures.[1] To a mixture of 3,4-(methylenedioxy)acetanilide (1.12 g, 6.23 mmol, 1.10 Equiv.) and AcOH (971 µL, 17.0 mmol, 3.00 Equiv.) in CH_2Cl_2 (13.5 mL, 0.42 M) at room temperature was added a solution of ICl in CH_2Cl_2 (919 mg, 0.57 M, 5.66 mmol, 1.00 Equiv.) *via* syringe. After 24 hours, sat. aq. $Na_2S_2O_5$ was added, and the organic phase collected. The aqueous phase was further extracted with CH_2Cl_2 (10.0 mL). The combined organic extracts were dried over Na_2SO_4, filtered, and concentrated under reduced pressure to afford the crude product which was purified by flash column chromatography (silica gel, 20 to 30% EtOAc in petroleum ether) to afford the title compound as a white solid (500 mg, 29%). Spectral data is in agreement with literature reports.[1]

^{1}H NMR (400 MHz, $CDCl_3$) δ 7.71 (s, 1H), 7.19 (br s, 1H), 7.17 (s, 1H), 5.98 (s, 2H), 2.21 (s, 3H).

4.2 Bdan-Capped Alkyne Synthesis

2-(Hex-1-yn-1-yl)-2,3-dihydro-1*H*-naphtho[1,8-*de*][1,3,2]diazaborinine (3a)

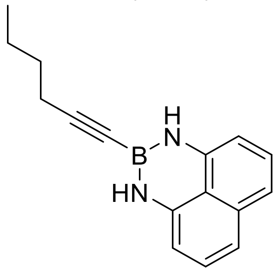

To a flame-dried Schlenk flask under an N_2 atmosphere was added 1-hexyne (750 µL, 6.60 mmol, 1.10 Equiv.) and anhydrous THF (20.0 mL). The mixture was cooled to −84 ˚C and *n*-butyllithium (2.5 M in hexane, 2.40 mL, 6.00 mmol, 1.00 Equiv.) was added dropwise. The mixture was stirred at the same temperature for 1 hour before B(O*i*Pr)$_3$ (1.37 mL, 6.00 mmol, 1.00 Equiv.) was added dropwise resulting in the rapid formation of a white precipitate. The mixture was stirred for a further 2 hours at −84 ˚C before HCl (2.0 M in Et_2O, 3.15 mL, 6.3 mmol, 1.05 Equiv.) was added dropwise and the mixture was allowed to warm to room temperature. The mixture was stirred for a further 30 minutes over which time a pale-yellow homogenous solution had formed. The mixture was then concentrated under reduced pressure before the residue was suspended in MTBE (10.0 mL) and filtered through a plug of celite. The mixture was again concentrated under reduced pressure before toluene (15.0 mL) and 1,8-diaminonaphthalene (996 mg, 6.3 mmol, 1.05 Equiv.) were sequentially added. The mixture was then heated at 100 ˚C in a sand bath for 1 hour before being concentrated under reduced pressure to afford a purple residue. The crude product was then purified by flash column chromatography (silica gel, 0 to 5% EtOAc in hexane.) to afford the title compound as a white solid (1.15 g, 77%). Spectral data is in agreement with literature reports.[4]

^{1}H NMR (400 MHz, $CDCl_3$) δ 7.10 (dd, *J* = 8.3, 7.3 Hz, 2H), 7.02 (dd, *J* = 8.4, 1.0 Hz, 2H), 6.30 (dd, *J* = 7.3, 1.1 Hz, 2H), 5.79 (s, 2H), 2.32 (t, *J* = 7.0 Hz, 2H), 1.60 – 1.52 (m, 2H), 1.52 – 1.41 (m, 2H), 0.97 (t, *J* = 7.3 Hz, 3H).

2-(Pent-1-yn-1-yl)-2,3-dihydro-1*H*-naphtho[1,8-*de*][1,3,2]diazaborinine (3b)

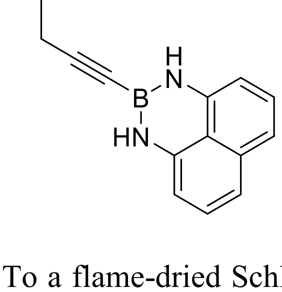

To a flame-dried Schlenk flask under an N_2 atmosphere was added 1-pentyne (300 µL, 3.04 mmol, 1.00 Equiv.) and anhydrous THF (10.0 mL.) The mixture was cooled to −84 ˚C and *n*-butyllithium (2.3 M in hexane, 1.78 mL, 4.08 mmol, 1.34 Equiv.) was added dropwise. The mixture was stirred at the same temperature for 1 hour before B(O*i*Pr)$_3$ (700 µL, 3.04 mmol, 1.00 Equiv.) was added dropwise resulting in the rapid formation of a white precipitate. The mixture was stirred for a further 2 hours at −84 ˚C before HCl (2.0 M in Et_2O, 1.60 mL, 3.20 mmol, 1.05 Equiv.) was added dropwise and the mixture was allowed to warm to room temperature. The mixture was stirred for a further 30 minutes over which time a pale-yellow homogenous solution had formed. The mixture was concentrated under reduced pressure

before the residue was suspended in MTBE (10.0 mL) and filtered through a plug of celite. The mixture was again concentrated under reduced pressure before toluene (15.0 mL) and 1,8-diaminonaphthalene (481 mg, 3.04 mmol, 1.00 Equiv.) were sequentially added. The mixture was then heated at 100 ˚C in a sand bath for 1 hour before being concentrated under reduced pressure to afford a purple residue. The crude product was then purified by flash column chromatography (silica gel, 0 to 5% EtOAc in hexane) to afford the title compound as a white solid (320 mg, 45%). Spectral data is in agreement with literature reports.[5]

^{1}H NMR (500 MHz, $CDCl_3$) δH 7.10 (dd, *J* = 8.3, 7.3 Hz, 2H), 7.03 (dd, *J* = 8.3, 1.02 Hz, 2H), 6.30 (dd, *J* = 7.3, 1.0 Hz, 2H), 5.88 – 5.73 (m, 2H), 2.30 (t, *J* = 7.1 Hz, 2H), 1.62 (h, *J* = 7.3 Hz, 2H), 1.06 (t, *J* = 7.4 Hz, 3H).

2-(Cyclopropylethynyl)-2,3-dihydro-1*H*-naphtho[1,8-*de*][1,3,2]diazaborinine (3c)

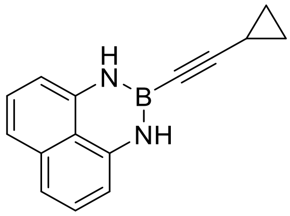

To a flame-dried Schlenk flask under an N_2 atmosphere was added ethynylcyclopropane (339 µL, 4.00 mmol, 1.00 Equiv.) and anhydrous THF (13.0 mL). The mixture was cooled to −5 °C, and ethylmagnesium bromide (1.45 mL, 2.9 M in Et_2O, 4.20 mmol, 1.05 Equiv.) was added dropwise. The mixture was subsequently stirred for 1 hour at the same temperature. To a separate flame-dried Schlenk flask under an N_2 atmosphere was added anhydrous THF (4.0 mL) and B(O*i*Pr)$_3$ (1.02 mL, 4.20 mmol, 1.05 Equiv.), and the mixture was cooled −84 °C. The Grignard solution was added to the borate solution dropwise *via* syringe over 15 minutes, and the mixture was stirred at −84 °C for 1 hour, before allowing to warm up to room temperature and stirring for further 40 minutes. The mixture was stirred for a further 1 hour at −84 ˚C before being allowed to warm to room temperature. HCl (2.0 M in Et_2O, 2.10 mL, 4.20 mmol, 1.05 Equiv.) was then added dropwise to the flask before the mixture was stirred for 5 minutes. The mixture was concentrated under reduced pressure and the residue suspended in MTBE (40.0 mL) and filtered through a plug of celite. The mixture was again concentrated under reduced pressure before toluene (20.0 mL) and 1,8-diaminonaphthalene (664 mg, 4.20 mmol, 1.05 Equiv.) were sequentially added. The mixture was then heated at 120 ˚C in a sand bath for 2 hours before being concentrated under reduced pressure to afford a purple residue. The crude product was then purified by flash column chromatography (silica, 0 to 5% Et_2O in hexane) to afford the title compound as a grey solid (282 mg, 30%). Spectral data is in agreement with literature reports.[6]

^{1}H NMR (500 MHz, $CDCl_3$) δ 7.08 (t, *J* = 7.8 Hz, 2H), 7.01 (d, *J* = 8.2 Hz, 2H), 6.27 (d, *J* = 7.3 Hz, 2H), 5.76 (s, 2H), 1.35 (tt, *J* = 8.3, 5.1 Hz, 1H), 0.89 – 0.83 (m, 2H), 0.82 – 0.77 (m, 2H).

2-Ethynyl-2,3-dihydro-1*H*-naphtho[1,8-*de*][1,3,2]diazaborinine (3e)

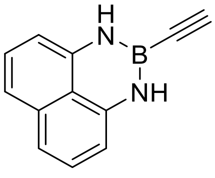

To a flame-dried Schlenk flask under an N_2 atmosphere, anhydrous THF (20.0 mL) and ethynylmagnesium bromide (0.36 M in THF, 27.8 mL, 10.0 mmol, 1.00 Equiv.) were sequentially added at –84 °C. The mixture was stirred for 15 minutes at –84 °C before $B(OMe)_3$ (1.34 mL, 12.0 mmol, 1.20 Equiv.) was added and the reaction stirred for 2 hours whilst allowing to warm to room temperature. To a separate flame-dried flask under an N_2 atmosphere was added 1,8-diaminonaphthalene (1.90 g, 12.0 mmol, 1.20 Equiv.) and anhydrous THF (5.00 mL). The diaminonaphthalene solution was then added to the ethynyl borate solution dropwise *via* syringe followed by dropwise addition of acetic acid (743 µL, 13.0 mmol, 1.30 Equiv.) before the mixture was stirred at room temperature for 2 hours. The reaction was quenched *via* addition of sat. aq. $NaHCO_3$ (20.0 mL) and extracted with EtOAc (2 × 50.0 mL). The combined organic extracts were then washed with brine and dried over Na_2SO_4 before being filtered and concentrated under reduced pressure. The crude product was then purified by flash column chromatography (silica gel, 0 to 5% Et_2O in hexane) to afford the title compound as a white solid (1.86 g, 97%). Spectral data is in agreement with literature reports.[7]

^{1}H NMR (400 MHz, $CDCl_3$) δ 7.15 – 7.07 (m, 2H), 7.05 (d, *J* = 8.3 Hz, 2H), 6.30 (d, *J* = 7.2, 2H), 5.85 (s, 2H), 2.61 (s, 1H).

((But-3-yn-1-yloxy)methyl)benzene

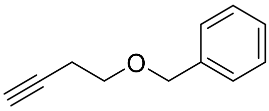

To a flame-dried flask under an N_2 atmosphere was added but-3-yn-1-ol (1.81 mL, 24.0 mmol, 1.20 Equiv.) and anhydrous THF (18.0 mL). The flask was cooled to 0 °C and NaH (960 mg, 60 wt% dispersion in mineral oil, 24.0 mmol, 1.20 Equiv.) was added portionwise followed by tetrabutylammonium iodide (739 mg, 2.00 mmol, 0.10 Equiv.) and benzyl bromide (2.38 mL, 20.0 mmol, 1.00 Equiv.) dropwise. The mixture was then allowed to warm to room temperature and stirred for 19 hours before the reaction was quenched *via* addition of sat. aq. NH_4Cl (15.0 mL) and extracted with Et_2O (3 × 20.0 mL). The combined organic extracts were then washed with brine and dried over Na_2SO_4 before being filtered and concentrated under reduced pressure. The crude product was then purified by flash column chromatography (silica gel, 0 to 5% Et_2O in hexane) to afford the title compound as a colorless liquid (2.87 g, 90%). Spectral data is in agreement with literature reports.

^{1}H NMR (500 MHz, $CDCl_3$) δ 7.43 – 7.38 (m, 4H), 7.37 – 7.32 (m, 1H), 4.61 (s, 2H), 3.65 (t, *J* = 6.9 Hz, 2H), 2.56 (td, *J* = 7.0, 2.7 Hz, 2H), 2.05 (t, *J* = 2.7 Hz, 1H).

2-(4-(Benzyloxy)but-1-yn-1-yl)-2,3-dihydro-1*H*-naphtho[1,8-*de*][1,3,2]diazaborinine (3f)

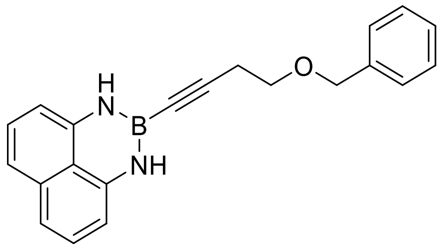

To a flame-dried Schlenk flask under an N_2 atmosphere was added ((but-3-yn-1-yloxy)methyl)benzene (1.64 mL, 10.0 mmol, 1.00 Equiv.) and anhydrous THF (30.0 mL). The mixture was cooled to −5 °C, and ethylmagnesium bromide (2.9 M in Et_2O, 3.68 mL, 10.5 mmol, 1.05 Equiv.) was added dropwise. The mixture was subsequently stirred for 1 hour at the same temperature. To a separate flame-dried Schlenk flask under an N_2 atmosphere was added anhydrous THF (10.0 mL) and B(O*i*Pr)$_3$ (2.55 mL, 10.5 mmol, 1.05 Equiv.), and the mixture was cooled −84 °C. The Grignard solution was added to the borate solution dropwise *via* syringe over 15 minutes, and the mixture was allowed to stir at −84 °C for 1 hour, before allowing to warm up to room temperature and stirring for further 40 minutes. HCl (2.0 M in Et_2O, 5.25 mL, 10.5 mmol, 1.05 Equiv.) was then added dropwise to the flask and the mixture was stirred for 5 minutes. The mixture was concentrated under reduced pressure and the residue suspended in MTBE (40.0 mL) and filtered through a plug of celite. The mixture was again concentrated under reduced pressure before toluene (50.0 mL) and 1,8-diaminonaphthalene (1.66 g, 10.5 mmol, 1.05 Equiv.) were sequentially added. The mixture was then stirred at 100 ˚C for 2 hours before being concentrated under reduced pressure to afford a purple residue. The crude product was then purified by flash column chromatography (silica gel, 0 to 5% Et_2O in hexane) to afford the title compound as a brown oil (1.19 g, 36%).

^{1}H NMR (500 MHz, $CDCl_3$) δ 7.46 – 7.42 (m, 4H), 7.45 – 7.33 (m, 1H), 7.14 (m, 2H), 7.08 (m, 2H), 6.29 (dd, *J* = 7.3, 1.9 Hz, 2H), 5.86 (s, 2H), 4.64 (s, 2H), 3.70 (t, *J* = 6.9 Hz, 2H), 2.69 (t, *J* = 6.9 Hz, 2H).

^{13}C{^{1}H} NMR (126 MHz, $CDCl_3$) δ 140.8, 138.0, 136.3, 128.5, 127.8, 127.6, 119.9, 117.9, 105.8, 101.5, 73.0, 68.0, 21.2.

Note: The carbon bearing boron was not observed due to quadrupolar relaxation.

^{11}B NMR (128 MHz, $CDCl_3$) δ 21.6.

IR (ATR, film): 3404, 2862, 2203, 1597, 1504, 1406, 1373, cm^{-1}.

HRMS (ESI) *m*/*z*: [M + H]+ Calcd for $C_{21}H_{20}B_1N_2O_1$ 327.1663; Found 327.1655

2-(4-(*tert*-Butyldimethylsiloxy)but-1-yn-1-yl)-2,3-dihydro-1*H*-naphtho[1,8-*de*][1,3,2]diazaborinine (3g)

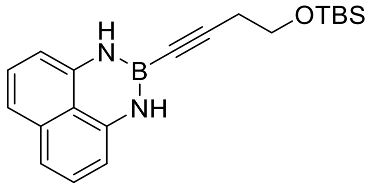

To a flame-dried Schlenk flask under an N_2 atmosphere was added (3-butyn-1-yloxy)(1,1-dimethylethyl)dimethylsilane (809 mg, 4.40 mmol, 1.10 Equiv.) and anhydrous THF (12.0 mL). The mixture was cooled to −84 ˚C and *n*-butyllithium (2.5 M in hexane, 1.60 mL, 4.00 mmol, 1.00 Equiv.) was added dropwise. The mixture was stirred at the same temperature for 1 hour before $B(O\textit{i}Pr)_3$ (910 µL, 4.00 mmol, 1.00 Equiv.) was added dropwise resulting in the rapid formation of a white precipitate. The mixture was stirred for a further 2 hours at −84 ˚C before HCl (2 M in Et_2O, 2.10 mL, 4.20 mmol, 1.05 Equiv.) was added dropwise and the mixture was allowed to warm to room temperature. The mixture was stirred for a further 30 minutes over which time a pale yellow homogenous solution had formed. The mixture was concentrated under reduced pressure and the residue suspended in MTBE (8.00 mL) and filtered through a plug of celite. The mixture was again concentrated under reduced pressure before toluene (10.0 mL) and 1,8-diaminonaphthalene (663 mg, 4.2 mmol, 1.05 Equiv.) were sequentially added. The mixture was then heated at 100 ˚C for 1 hour before being concentrated under reduced pressure to afford a purple residue. The crude product was then purified by flash column chromatography (silica gel, 0 to 5% EtOAc in hexane) to afford the title compound as a purple oil (1.16 g, 83%). Spectral data is in agreement with literature reports.[8]

^{1}H NMR (400 MHz, $CDCl_3$) δ 7.08 (dd, *J* = 8.3, 7.2 Hz, 2H), 7.00 (dd, *J* = 8.4, 1.0 Hz, 2H), 6.27 (dd, *J* = 7.2, 1.1 Hz, 2H), 5.76 (s, 2H), 3.78 (t, *J* = 7.1 Hz, 2H), 2.52 (t, *J* = 7.1 Hz, 2H), 0.92 (s, 9H), 0.10 (s, 6H).

2-(Phenylethynyl)-2,3-dihydro-1*H*-naphtho[1,8-*de*][1,3,2]diazaborinine (3h)

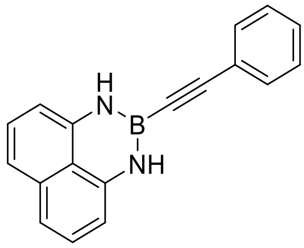

Prepared according to General procedure E from 2-ethynyl-2,3-dihydro-1*H*-naphtho[1,8-de][1,3,2]diazaborinine (192 mg, 1.00 mmol, 1.00 Equiv.), and iodobenzene (134 µL, 1.20 mmol, 1.20 Equiv.) at room temperature for 3 hours. Purified by flash column chromatography (silica gel, 0 to 3% Et_2O in hexane) to afford the title compound as a yellow solid (158 mg, 59%). Spectral data is in agreement with literature reports.[8]

^{1}H NMR (500 MHz, $CDCl_3$) δ 7.60 – 7.54 (m, 2H), 7.44 – 7.35 (m, 3H), 7.14 (dd, *J* = 8.3, 7.2 Hz, 2H), 7.08 (dd, *J* = 8.3, 1.1 Hz, 2H), 6.33 (dd, *J* = 7.3, 1.1 Hz, 2H), 5.92 (s, 2H).

2-(Cyclohex-1-en-1-ylethynyl)-2,3-dihydro-1*H*-naphtho[1,8-*de*][1,3,2]diazaborinine (3i)

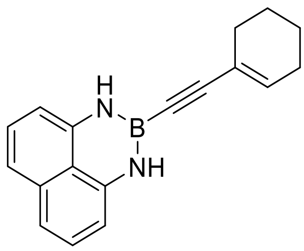

Prepared according to General procedure E from 2-ethynyl-2,3-dihydro-1*H*-naphtho[1,8-de][1,3,2]diazaborinine (192 mg, 1.00 mmol, 1.00 Equiv.), and cyclohex-1-en-1-yl trifluoromethanesulfonate (210 μL, 1.20 mmol, 1.20 Equiv.) at 50 °C for 3 hours. Purified by flash column chromatography (silica gel, 0 to 2% Et_2O in hexane) to afford the title compound as a yellow solid (144 mg, 53%). Spectral data is in agreement with literature reports.[8]

^{1}H NMR (500 MHz, $CDCl_3$) δ 7.09 (dd, *J* = 8.3, 7.3 Hz, 2H), 7.02 (dd, *J* = 8.4, 1.0 Hz, 2H), 6.30 – 6.24 (m, 3H), 5.81 (s, 2H), 2.22 – 2.11 (m, 4H), 1.70 – 1.57 (m, 4H).

5. Characterization Data

2-(3-Butyl-1*H*-indol-2-yl)-2,3-dihydro-1*H*-naphtho[1,8-*de*][1,3,2]diazaborinine (4a)

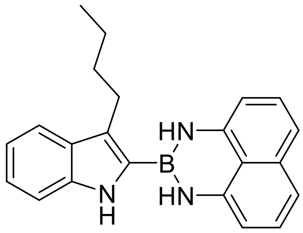

Prepared according to General procedure D from 2-(hex-1-yn-1-yl)-2,3-dihydro-1*H*-naphtho[1,8-*de*][1,3,2]diazaborinine (99.3 mg, 400 µmol, 2.00 Equiv.) and 2-iodoaniline (43.7 mg, 200 µmol, 1.00 Equiv.). Purified by flash column chromatography (silica gel, 0 to 10% EtOAc in hexane) to yield the title compound as a white solid which quickly discolors (67.0 mg, 99%).

^{1}H NMR (400 MHz, $CDCl_3$) δ 7.99 (s, 1H), 7.70 (d, *J* = 7.9 Hz, 1H), 7.41 (d, *J* = 8.1 Hz, 1H), 7.28 (ddd, *J* = 8.1, 6.9, 1.2 Hz, 1H , *overlaps with* $CDCl_3$ *signal*), 7.23 – 7.14 (m, 3H), 7.12 (d, *J* = 8.1 Hz, 2H), 6.43 (d, *J* = 7.0 Hz, 2H), 5.97 (s, 2H), 2.98 (t, *J* = 7.5 Hz, 2H), 1.81 – 1.71 (m, 2H), 1.49 (dq, *J* = 14.5, 7.3 Hz, 2H), 1.01 (t, *J* = 7.3 Hz, 3H).

^{13}C{^{1}H} NMR (101 MHz, $CDCl_3$) δ 140.8, 138.3, 136.5, 128.8, 127.8, 125.8, 123.3, 119.9, 119.7, 119.5, 118.3, 111.3, 106.4, 34.4, 25.7, 23.1, 14.2.

Note: The carbon bearing boron was not observed due to quadrupolar relaxation.

^{11}B NMR (128 MHz, $CDCl_3$) δ 27.4.

IR (ATR, film) ν_{max} 3408, 2926, 1595, 1545, 1499, 1402 cm^{-1}.

HRMS (ESI) *m*/*z*: [M + H]+ Calcd for $C_{22}H_{23}B_1N_3$ 340.1980; Found 340.1983

2-(3-Butyl-5-methyl-1*H*-indol-2-yl)-2,3-dihydro-1*H*-naphtho[1,8-*de*][1,3,2]diazaborinine (4b)

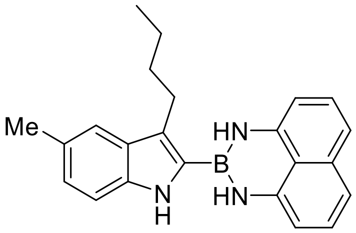

Prepared according to General procedure D from 2-(hex-1-yn-1-yl)-2,3-dihydro-1*H*-naphtho[1,8-*de*][1,3,2]diazaborinine (99.3 mg, 400 µmol, 2.00 Equiv.) and 4-methyl-2-iodoaniline (46.4 mg, 200 µmol, 1.00 Equiv.). Purified by flash column chromatography (silica gel, 0 to 10% EtOAc in hexane) to yield the title compound as a white solid which quickly discolors brown (48.0 mg, 68%).

^{1}H NMR (400 MHz, $CDCl_3$) δ 7.88 (s, 1H), 7.44 (d, *J* = 0.9 Hz, 1H), 7.28 (d, *J* = 8.4 Hz, 1H), 7.17 (dd, *J* = 8.3, 7.2 Hz, 2H), 7.09 (ddd, *J* = 8.3, 3.5, 1.3 Hz, 3H), 6.40 (dd, *J* = 7.2, 1.1 Hz, 2H), 5.94 (s, 2H), 2.93 (t, *J* = 7.7 Hz, 2H), 2.50 (s, 3H), 1.73 (p, *J* = 7.5 Hz, 2H), 1.48 (h, *J* = 7.3 Hz, 2H), 1.00 (t, *J* = 7.3 Hz, 3H).

^{13}C{^{1}H} NMR (101 MHz, $CDCl_3$) δ 140.9, 136.7, 136.5, 129.1, 128.7, 127.8, 125.4, 125.0, 119.9, 119.2, 118.2, 110.9, 106.3, 34.4, 25.7, 23.1, 21.7, 14.2.

Note: The carbon bearing boron was not observed due to quadrupolar relaxation.

^{11}B NMR (128 MHz, $CDCl_3$) δ 28.0

IR (ATR, film) ν_{max} 3406, 3302, 2951, 2918, 2852, 1625 1600, 1552, 1541 cm^{-1}.

HRMS (ESI) *m*/*z*: [M + H]+ Calcd for $C_{23}H_{25}B_1N_3$ 354.2136; Found 354.2146

2-(3-Butyl-5-fluoro-1*H*-indol-2-yl)-2,3-dihydro-1*H*-naphtho[1,8-*de*][1,3,2]diazaborinine (4c)

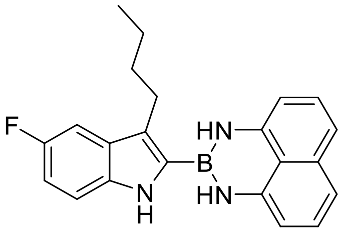

Prepared according to General procedure D from 2-(hex-1-yn-1-yl)-2,3-dihydro-1*H*-naphtho[1,8-*de*][1,3,2]diazaborinine (99.3 mg, 400 µmol, 2.00 Equiv.) and 4-fluoro-2-iodoaniline (47.2 mg, 200 µmol, 1.00 Equiv.). Purified by flash column chromatography (silica gel, 0 to 10% EtOAc in hexane) to yield the title compound as a white solid which quickly discolors. (66.4 mg, 93%).

^{1}H NMR (400 MHz, $CDCl_3$) δ 8.00 (s, 1H), 7.33 – 7.23 (m, 2H, *overlaps with $CDCl_3$ signal*), 7.16 (dd, *J* = 8.3, 7.2 Hz, 2H), 7.09 (d, *J* = 8.3 Hz, 2H), 6.99 (td, *J* = 9.0, 2.5 Hz, 1H), 6.41 (d, *J* = 7.2 Hz, 2H), 5.94 (s, 2H), 2.89 (t, *J* = 7.7 Hz, 2H), 1.70 (tt, *J* = 7.8, 6.4 Hz, 2H), 1.45 (h, *J* = 7.4 Hz, 2H), 0.98 (t, *J* = 7.3 Hz, 3H).

^{13}C{^{1}H} NMR (101 MHz, $CDCl_3$) δ 157.7 (d, J_{CF} = 235.1 Hz), 140.7, 136.5 134.8, 129.2 (d, *J* = 9.0 Hz), 127.8, 125.7 (d, J_{CF} = 4.9 Hz), 120.0, 118.4, 111.8 (d, J_{CF} = 23.3 Hz) 111.8 (d, J_{CF} = 9.4 Hz), 106.44, 104.3 (d, J_{CF} = 23.3 Hz), 34.2, 25.7, 23.0, 14.2.

Note: The carbon bearing boron was not observed due to quadrupolar relaxation.

^{19}F NMR (376 MHz, $CDCl_3$) δ −124.7

^{11}B NMR (128 MHz, $CDCl_3$) δ 27.5

IR (ATR, film) ν_{max} 3435, 3412, 3055, 3028, 2954, 2922, 1629, 1598 cm^{-1}.

HRMS (ESI) *m*/*z*: [M + H]+ Calcd for $C_{22}H_{22}B_1F_1N_3$ 358.1885; Found 358.1875

2-(3-Butyl-5-trifluoromethyl-1*H*-indol-2-yl)-2,3-dihydro-1*H*-naphtho[1,8-*de*][1,3,2]diazaborinine (4d)

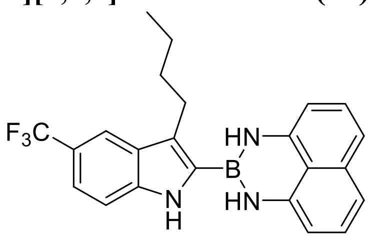

Prepared according to General procedure D from 2-(hex-1-yn-1-yl)-2,3-dihydro-1*H*-naphtho[1,8-*de*][1,3,2]diazaborinine (99.3 mg, 400 µmol, 2.00 Equiv.) and 2-iodo-4-trifluoromethylaniline (57.2 mg, 200 µmol, 1.00 Equiv.). Purified by flash column chromatography (silica gel, 0 to 10% EtOAc in hexane) to yield the title compound as a white solid which quickly discolors green (47.1 mg, 66%).

^{1}H NMR (400 MHz, $CDCl_3$) δ 8.20 (s, 1H), 7.93 (d, J = 0.8 Hz, 1H), 7.46 (dd, J = 8.6, 1.6 Hz, 1H), 7.43 (d, J = 8.6 Hz, 1H), 7.17 (dd, J = 8.3, 7.2 Hz, 2H), 7.10 (d, J = 8.3 Hz, 2H), 6.41 (d, J = 7.2 Hz, 2H), 5.96 (s, 2H), 3.20 (t, J = 7.5 Hz), 1.72 (p, J = 7.5 Hz, 2H), 1.46 (h, J = 7.3 Hz, 2H), 0.99 (t, J = 7.3 Hz, 3H).

^{13}C{^{1}H} NMR (101 MHz, $CDCl_3$) δ 140.4, 139.3, 128.0, 127.7, 126.3, 125.4 (q, J_{CF} = 272 Hz), 122.0, 121.7, 119.9, 119.8 (q, J_{CF} = 3.2 Hz), 118.4, 117.4 (q, J_{CF} = 4.3 Hz), 111.4, 106.4, 34.2, 25.4, 22.9, 14.0.

Note: The carbon bearing boron was not observed due to quadrupolar relaxation.

^{19}F NMR (376 MHz, $CDCl_3$) δ –60.2.

^{11}B NMR (128 MHz, $CDCl_3$) δ 27.4.

IR (ATR, film) ν_{max} 3404, 3335, 2926, 2854, 1627, 1598 cm^{-1}.

HRMS (ESI) *m*/*z*: [M + H]+ Calcd for $C_{23}H_{22}B_1F_3N_3$ 408.1853; Found 408.1837

2-(3-Butyl-5-chloro-1*H*-indol-2-yl)-2,3-dihydro-1*H*-naphtho[1,8-*de*][1,3,2]diazaborinine (4e)

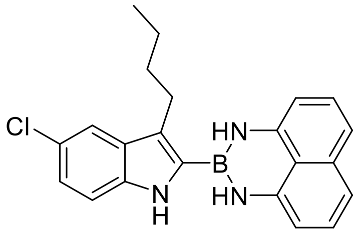

Prepared according to General procedure D from 2-(hex-1-yn-1-yl)-2,3-dihydro-1*H*-naphtho[1,8-*de*][1,3,2]diazaborinine (99.3 mg, 400 µmol, 2.00 Equiv.) and 4-chloro-2-iodoaniline (50.7 mg, 200 µmol, 1.00 Equiv.). Purified by flash column chromatography (silica gel, 0 to 10% EtOAc in hexane) to yield the title compound as a white solid which quickly discolors brown (38.1 mg, 51%).

^{1}H NMR (700 MHz, $CDCl_3$) δ 8.02 (s, 1H), 7.60 (t, *J* = 1.4 Hz, 1H), 7.28 – 7.25 (m, 1H), 7.20 – 7.14 (m, 3H), 7.10 (d, *J* = 8.2 Hz, 2H), 6.39 (d, *J* = 7.2 Hz, 2H), 5.93 (s, 2H), 2.87 (t, *J* = 7.8 Hz, 2H), 1.69 (ddd, *J* = 15.4, 8.4, 6.8 Hz, 2H), 1.45 (h, *J* = 7.4 Hz, 2H), 0.98 (td, *J* = 7.3, 1.2 Hz, 3H).

^{13}C{^{1}H} NMR (176 MHz, $CDCl_3$) δ 140.62, 136.5, 136.4, 129.9, 127.8, 125.2, 123.5, 119.9, 119.0, 118.4, 112.2, 106.4, 34.2, 25.6, 23.0, 14.2.

Note: The carbon bearing boron was not observed due to quadrupolar relaxation.

^{11}B NMR (128 MHz, $CDCl_3$) δ 26.9.

IR (ATR, film) ν_{max} 3414, 2926, 1597, 1543, 1499, 1462, 1402, 1373 cm^{-1}.

HRMS (ESI) *m*/*z*: [M + H]+ Calcd for $C_{22}H_{22}B_1Cl_1N_3$ 374.1590; Found 374.1593

Methyl-3-butyl-2-(1*H*-naphtho[1,8-*de*][1,3,2]diazaborinin-2(3*H*)-yl)-1*H*-indole-5-carboxylate (4f)

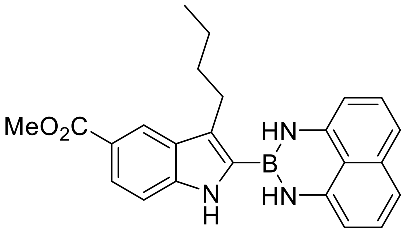

Prepared according to General procedure D from 2-(hex-1-yn-1-yl)-2,3-dihydro-1*H*-naphtho[1,8-*de*][1,3,2]diazaborinine (99.3 mg, 400 µmol, 2.00 Equiv.) and methyl 4-amino-3-iodobenzoate (55.4 mg, 200 µmol, 1.00 Equiv.). Purified by flash column chromatography (silica gel, 0 to 15% EtOAc in hexane) to yield the title compound as a white solid (76.0 mg, 96%).

^{1}H NMR (500 MHz, $CDCl_3$) δ 8.41 (s, 1H), 8.35 (s, 1H), 7.92 (dd, *J* = 8.5, 1.6 Hz, 1H), 7.36 (d, *J* = 8.6 Hz, 1H), 7.15 (t, *J* = 7.8 Hz, 3H), 7.09 (d, *J* = 8.2 Hz, 2H), 6.4 (d, *J* = 7.22 Hz, 2H), 6.02 (s, 2H), 3.96 (s, 3H), 2.96 (t, *J* = 7.8 Hz, 2H), 1.73 (p, *J* = 7.5 Hz, 2H), 1.46 (h, *J* = 7.4 Hz, 2H), 0.98 (t, *J* = 7.4 Hz, 3H).

^{13}C{^{1}H} NMR (126 MHz, $CDCl_3$) δ 168.5, 140.7, 140.6, 136.5, 128.5, 127.8, 127.1, 124.5, 122.8, 121.6, 120.0, 118.4, 110.9, 106.5, 52.1, 34.5, 25.6, 23.0, 14.2.

Note: The carbon bearing boron was not observed due to quadrupolar relaxation.

^{11}B NMR (128 MHz, $CDCl_3$) δ 26.6.

IR (ATR, film) ν_{max} 3321, 1686, 1599, 1547, 1506, 1433 cm^{-1}.

HRMS (ESI) *m*/*z*: [M + H]+ Calcd for $C_{24}H_{25}B_1N_3O_2$ 398.2034; Found 398.2037

3-Butyl-2-(1*H*-naphtho[1,8-*de*][1,3,2]diazaborinin-2(3*H*)-yl)-1*H*-indole-5-carbonitrile (4g)

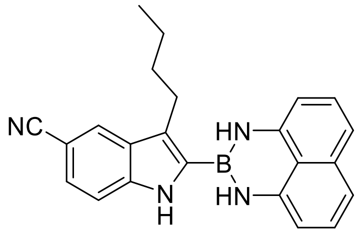

Prepared according to General procedure D from 2-(hex-1-yn-1-yl)-2,3-dihydro-1*H*-naphtho[1,8-*de*][1,3,2]diazaborinine (99.3 mg, 400 µmol, 2.00 Equiv.) and 4-amino-3-iodobenzonitrile (48.8 mg, 200 µmol, 1.00 Equiv.). Purified by flash column chromatography (silica gel, 0 to 15% EtOAc in hexane) to yield the title compound as a white solid which quickly discolors dark green (22.3 mg, 31%).

^{1}H NMR (700 MHz, $CDCl_3$) δ 8.66 (s, 1H), 7.98 (s, 1H), 7.41 (s, 2H), 7.18 – 7.01 (m, 4H), 6.42 (d, *J* = 7.2 Hz, 2H), 6.06 (s, 2H), 2.92 (t, *J* = 7.8 Hz, 2H), 1.69 (p, *J* = 7.6 Hz, 2H), 1.45 (h, *J* = 7.4 Hz, 2H), 0.99 (t, *J* = 7.4 Hz, 3H).

^{13}C{^{1}H} NMR (176 MHz, $CDCl_3$) δ 140.5, 139.7, 136.4, 134.0, 128.6, 127.8, 126.1, 125.7, 125.5, 121.2, 120.0, 118.5, 112.1, 106.5, 102.3, 34.3, 25.5, 23.0, 14.2.

^{11}B NMR (128 MHz, $CDCl_3$) δ 28.4.

IR (ATR, film) ν_{max} 3368, 2924, 2216, 1599, 1549, 1410 cm^{-1}.

HRMS (ESI) *m*/*z*: [M + H]+ Calcd for $C_{23}H_{22}B_1N_4$ 365.1932; Found 365.1920

2-(3-Butyl-4-methyl-1*H*-indol-2-yl)-2,3-dihydro-1*H*-naphtho[1,8-*de*][1,3,2]diazaborinine (4h)

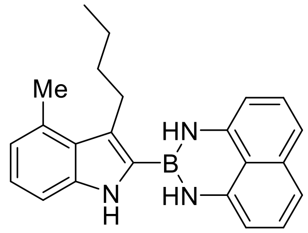

Prepared according to General procedure D from 2-(hex-1-yn-1-yl)-2,3-dihydro-1*H*-naphtho[1,8-*de*][1,3,2]diazaborinine (99.3 mg, 400 µmol, 2.00 Equiv.) and 2-iodo-3-methylaniline (46.4 mg, 200 µmol, 1.00 Equiv.). Purified by flash column chromatography (silica gel, 0 to 10% EtOAc in hexane) to yield the title compound as a white solid which quickly discolors black (24.0 mg, 34%).

^{1}H NMR (400 MHz, $CDCl_3$) δ 8.11 (s, 1H), 7.22 (d, J = 8.2 Hz, 1H), 7.14 (q, J = 7.6 Hz, 2H), 7.11 – 7.04 (m, 3H), 6.85 (d, J = 7.0 Hz, 1H), 6.40 (d, J = 7.2 Hz, 2H), 5.99 (s, 2H), 3.04 (t, J = 7.7 Hz, 2H), 2.74 (s, 3H), 1.70 (p, J = 7.6 Hz, 2H), 1.52 (h, J = 7.4 Hz, 2H), 1.01 (t, J = 7.3 Hz, 3H).

^{13}C{^{1}H} NMR (101 MHz, $CDCl_3$) δ 140.8, 138.8, 136.5, 131.5, 127.8, 127.0, 126.7, 123.3, 121.6, 118.27, 118.1, 109.2, 106.3, 36.9, 27.2, 23.0, 20.4, 14.2.

Note: The carbon bearing boron was not observed due to quadrupolar relaxation.

^{11}B NMR (128 MHz, $CDCl_3$) δ 28.5

IR (ATR, film) ν_{max} 3338, 3059, 2941, 2538, 1622, 1600, 1558 cm^{-1}.

HRMS (ESI) *m*/*z*: [M + H]+ Calcd for $C_{23}H_{25}B_1N_3$ 354.2136; Found 354.2129

2-(3-Butyl-6-chloro-1*H*-indol-2-yl)-2,3-dihydro-1*H*-naphtho[1,8-*de*][1,3,2]diazaborinine (4i)

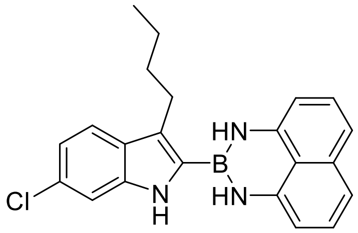

Prepared according to General procedure D from 2-(hex-1-yn-1-yl)-2,3-dihydro-1*H*-naphtho[1,8-*de*][1,3,2]diazaborinine (99.3 mg, 400 μmol, 2.00 Equiv.) and 2-iodo-5-chloroaniline (50.4 mg, 200 μmol, 1.00 Equiv.). Purified by flash column chromatography (silica gel, 0 to 10% EtOAc in hexane) to yield the title compound as a brown solid (52.0 mg, 69%).

^{1}H NMR (400 MHz, $CDCl_3$) δ 8.00 (s, 1H), 7.54 (d, J = 8.4 Hz, 1H), 7.36 (d, J = 1.8 Hz, 1H), 7.16 (dd, J = 8.3, 7.2 Hz, 2H), 7.09 (dd, J = 8.5, 1.8 Hz, 3H), 6.41 (d, J = 7.2 Hz, 2H), 5.94 (s, 2H), 2.99 – 2.87 (m, 2H), 1.70 (p, J = 7.5 Hz, 2H), 1.44 (dq, J = 14.4, 7.3 Hz, 2H), 0.97 (t, J = 7.4 Hz, 3H).

^{13}C{^{1}H} NMR (101 MHz, $CDCl_3$) δ 140.7, 138.6, 129.2, 127.8, 127.5, 125.8, 120.5, 120.4, 120.2, 120.0, 118.4, 111.1, 106.4, 34.3, 25.6, 23.0, 14.2.

Note: The carbon bearing boron was not observed due to quadrupolar relaxation.

^{11}B NMR (128 MHz, $CDCl_3$) δ 28.3

IR (ATR, film) ν_{max} 3423, 3392, 2954, 2922, 2852, 1683, 1595, 1541 cm^{-1}.

HRMS (ESI) *m*/*z*: [M + H]+ Calcd for $C_{22}H_{22}B_1Cl_1N_3$ 374.1590; Found 374.1593.

2-(3-Propyl-1*H*-indol-2-yl)-2,3-dihydro-1*H*-naphtho[1,8-*de*][1,3,2]diazaborinine (4j)

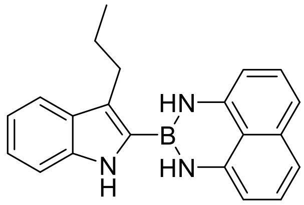

Prepared according to General procedure D from 2-(pent-1-yn-1-yl)-2,3-dihydro-1*H*-naphtho[1,8-*de*][1,3,2]diazaborinine (93.6 mg, 400 µmol, 2.00 Equiv.) and 2-iodoaniline (43.7 mg, 200 µmol, 1.00 Equiv.). Purified by flash column chromatography (silica gel, 0 to 10% EtOAc in cyclohexane) to yield the title compound as a white solid which quickly discolors purple (64.6 mg, 99%).

^{1}H NMR (500 MHz, $CDCl_3$) δ 8.00 (s, 1H), 7.67 (d, *J* = 7.9 Hz, 1H), 7.39 (d, *J* = 8.1 Hz, 1H), 7.30 – 7.22 (m, 3H), 7.15 – 7.07 (m, 2H), 6.41 (d, *J* = 7.4 Hz, 2H), 5.96 (s, 2H), 2.94 (t, *J* = 7.6 Hz, 2H), 1.79 (h, *J* = 7.4 Hz, 2H), 1.04 (t, *J* = 7.3 Hz, 3H).

^{13}C{^{1}H} NMR (126 MHz, $CDCl_3$) δ 140.8, 138.2, 136.5, 128.8, 127.8, 125.5, 123.2, 119.9, 119.7, 119.5, 118.2, 111.3, 106.4, 28.0, 25.2, 14.5.

Note: The carbon bearing boron was not observed due to quadrupolar relaxation.

^{11}B NMR (128 MHz, $CDCl_3$) δ 27.8

IR (ATR, film) ν_{max} 3402, 1597, 1545, 1499, 1404, 1373 cm^{-1}.

HRMS (ESI) *m*/*z*: [M + H]+ Calcd for $C_{21}H_{21}B_1N_3$ 326.1823; Found 326.1821.

2-(3-Cyclopropyl-1*H*-indol-2-yl)-2,3-dihydro-1*H*-naphtho[1,8-*de*][1,3,2]diazaborinine (4k)

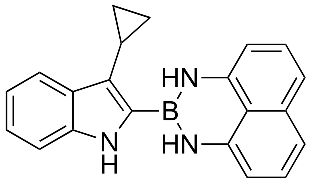

Prepared according to General procedure D from 2-(cyclopropylethynyl)-2,3-dihydro-1*H*-naphtho[1,8-*de*][1,3,2]diazaborinine (92.8 mg, 400 µmol, 2.00 Equiv.) and 2-iodoaniline (43.8 mg, 200 µmol, 1.00 Equiv.). Purified by flash column chromatography (silica gel, 0 to 10% EtOAc in hexane) to afford the title compound as a dark green solid (19.0 mg, 29%).

^{1}H NMR (400 MHz, $CDCl_3$) δ 8.06 (s, 1H), 7.84 (d, J = 8.0 Hz, 1H), 7.38 (d, J = 8.3 Hz, 1H), 7.29 – 7.22 (m, 1H, *Overlaps with CDCl3 signal*), 7.16 (m, 3H), 7.08 (d, J = 7.9 Hz, 2H), 6.43 (d, J = 7.7 Hz, 2H), 6.30 (s, 2H), 2.07 (q, J = 6.3 Hz, 1H), 1.13 – 1.03 (m, 2H), 0.88 – 0.82 (m, 2H).

^{13}C{^{1}H} NMR (126 MHz, $CDCl_3$) δ 141.0, 137.9, 136.5, 130.8, 129.4, 127.8, 126.7, 123.4, 120.3, 119.8, 119.7, 118.1, 111.3, 106.2, 6.8, 6.7.

Note: The carbon bearing boron was not observed due to quadrupolar relaxation.

^{11}B NMR (128 MHz, $CDCl_3$) δ 27.1.

IR (ATR, film) ν_{max} 3431, 3041, 2924, 1595, 1543, 1404 cm^{-1}.

HRMS (ESI) *m/z*: [M + H]+ Calcd for $C_{21}H_{19}B_1N_3$ 324.1666; Found 324.1666.

2-(3-(2-(Benzyloxy)ethyl)-1*H*-indol-2-yl)-2,3-dihydro-1*H*-naphtho[1,8-*de*][1,3,2]diazaborinine (4l)

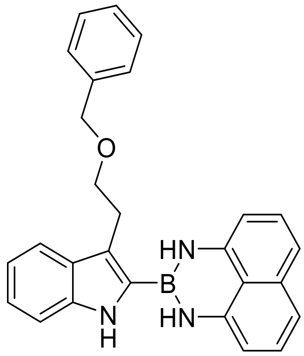

Prepared according to General procedure D from 2-(4-(benzyloxy)but-1-yn-1-yl)-2,3-dihydro-1*H*-naphtho[1,8-*de*][1,3,2]diazaborinine (130 mg, 400 µmol, 2.00 Equiv.), and 2-iodoaniline (43.8 mg, 200 µmol, 1.00 Equiv.). Purified by flash column chromatography (silica gel, 10 to 20% Et_2O in hexane) to afford the title compound as a brown solid (49.0 mg, 59%).

^{1}H NMR (400 MHz, $CDCl_3$) δ 8.12 (s, 1H), 7.58 (d, J = 7.8 Hz, 1H), 7.43 (d, J = 8.1 Hz, 1H), 7.34 – 7.22 (m, 8H, *overlaps with* $CDCl_3$ *signal*), 7.14 (t, J = 7.5 Hz, 1H), 7.06 – 6.95 (m, 4H), 6.83 (s, 2H), 6.01 (dd, J = 6.6, 1.8 Hz, 2H), 3.90 (t, J = 5.2 Hz, 2H), 3.23 (t, J = 5.2 Hz, 2H).

^{13}C{^{1}H} NMR (126 MHz, $CDCl_3$) δ 141.3, 138.2, 137.8, 136.5, 128.7, 128.6, 128.3, 128.0, 127.6, 123.1, 121.9, 120.2, 119.6, 118.9, 117.6, 111.4, 106.1, 73.9, 71.5, 26.5.

Note: The carbon bearing boron was not observed due to quadrupolar relaxation.

^{11}B NMR (128 MHz, $CDCl_3$) δ 27.4.

IR (ATR, film) ν_{max} 3404, 3323, 2924, 2855, 1597, 1551, 1506 cm^{-1}.

HRMS (ESI) *m/z*: [M + H]+ Calcd for $C_{27}H_{25}B_1N_3O_1$ 418.2085; Found 418.2089.

2-(3-[*tert*-Butyldimethylsiloxyethyl]-1*H*-indol-2-yl)-2,3-dihydro-1*H*-naphtho[1,8-*de*][1,3,2]diazaborinine (4m)

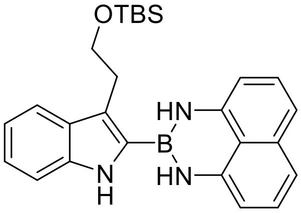

Prepared according to General procedure D from 2-(4-(1,1-dimethylethyl)dimethylsilyl]oxy)but-1-yn-1-yl)-2,3-dihydro-1H-naphtho[1,8-de][1,3,2]diazaborinine (140.3 mg, 400 µmol, 2.00 Equiv.) and 2-iodoaniline (43.7 mg, 200 µmol, 1.00 Equiv.). Purified by flash column chromatography (silica gel, 0 to 10% EtOAc in hexane) to yield the title compound as a white solid which quickly discolors purple (67.1 mg, 99%).

^{1}H NMR (400 MHz, $CDCl_3$) δ 8.11 (s, 1H), 7.59 (d, *J* = 7.8 Hz, 1H), 7.41 (d, *J* = 8.1 Hz, 1H), 7.32 – 7.20 (m, 1H), 7.20 – 7.11 (m, 3H), 7.06 (d, *J* = 8.2 Hz, 2H), 6.79 (s, 2H), 6.41 (d, *J* = 7.2 Hz, 2H), 4.05 (t, *J* = 5.5 Hz, 2H), 3.19 (t, *J* = 5.5 Hz, 2H), 0.75 (s, 9H), -0.16 (s, 6H).

^{13}C{^{1}H} NMR (101 MHz, $CDCl_3$) δ 141.4, 138.3, 136.6, 128.5, 127.8, 123.0, 121.8, 120.3, 119.6, 118.9, 117.8, 111.3, 106.2, 64.2, 28.7, 26.1, 18.8 -5.5.

Note: The carbon bearing boron was not observed due to quadrupolar relaxation.

^{11}B NMR (128 MHz, $CDCl_3$) δ 28.3.

IR (ATR, film) ν_{max} 3408, 3317, 2947, 2926, 2852, 2377, 1597, 1506 cm^{-1}.

HRMS (ESI) *m/z*: [M + H]+ Calcd for $C_{26}H_{33}B_{1}N_{3}O_{1}Si_{1}$ 442.2481; Found 442.2480.

2-(3-Butyl-5-(1,1-Dimethylethyl)dimethylsilyl]oxy]methyl)-1*H*-indol-2-yl)-2,3-dihydro-1*H*-naphtho[1,8-*de*][1,3,2]diazaborinine (4n)

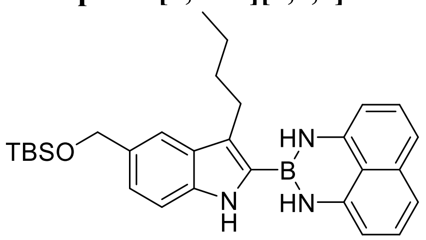

Prepared according to General procedure D from 2-(hex-1-yn-1-yl)-2,3-dihydro-1*H*-naphtho[1,8-*de*][1,3,2]diazaborinine (99.3 mg, 400 µmol, 2.00 Equiv.) and 4-[[[(1,1-dimethylethyl)dimethylsilyl]oxy]methyl]-2-iodoaniline (72.6 mg, 200 µmol, 1.00 Equiv.). Purified by flash column chromatography (silica gel, 0 to 10% EtOAc in hexane) to yield the title compound as a white solid which quickly discolors (55.9 mg, 59%).

^{1}H NMR (400 MHz, $CDCl_3$) δ 7.97 (s, 1H), 7.62 (p, J = 0.8 Hz, 1H), 7.32 (dd, J = 8.4, 0.7 Hz, 1H), 7.26 – 7.19 (m, 1H), 7.16 (dd, J = 8.3, 7.2 Hz, 2H), 7.09 (dd, J = 8.3, 1.1 Hz, 2H), 6.39 (dd, J = 7.2, 1.1 Hz, 2H), 5.95 (s, 2H), 4.89 (s, 2H), 2.99 – 2.88 (m, 2H), 1.78 – 1.66 (m, 2H), 1.46 (h, J = 7.3 Hz, 2H), 1.04 – 0.94 (m, 12H), 0.15 (s, 6H).

^{13}C{^{1}H} NMR (101 MHz, $CDCl_3$) δ 140.9, 137.6, 136.5, 132.5, 128.7, 127.8, 125.8, 122.5, 112.0, 118.2, 117.5, 111.1, 106.3, 66.0, 34.4, 26.2, 25.7, 23.1, 18.7, 14.2, -4.9.

Note: The carbon bearing boron was not observed due to quadrupolar relaxation.

^{11}B NMR (128 MHz, $CDCl_3$) δ 27.6.

IR (ATR, film) ν_{max} 3402, 3329, 2949, 2924, 2852, 1627, 1597, 1550.

HRMS (ESI) *m*/*z*: [M + Na]+ Calcd for $C_{29}H_{38}B_{1}N_{3}O_{1}Si_{1}Na_{1}$ 506.2769; Found 506.2747.

2-(3-Butyl-1*H*-pyrrolo[2,3-*b*]pyridin-2-yl)-2,3-dihydro-1*H*-naphtho[1,8-*de*][1,3,2]diazaborinine (4o)

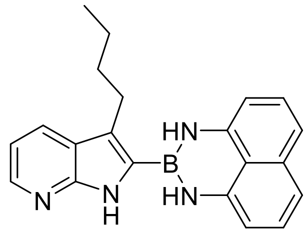

Prepared according to General procedure D from 2-(hex-1-yn-1-yl)-2,3-dihydro-1*H*-naphtho[1,8-*de*][1,3,2]diazaborinine (99.3 mg, 400 µmol, 2.00 Equiv.) and 3-iodopyridin-2-amine (44.0 mg, 200 µmol, 1.00 Equiv.). Purified by flash column chromatography (silica gel, 0 to 20% MeCN in CH_2Cl_2) to yield the title compound as a white solid (25.9 mg, 38%). Crystals suitable for SCXRD analysis were obtained by slow evaporation of an acetone solution of **4o**.

^{1}H NMR (500 MHz, Acetone *d*-6) δ 10.38 (s, 1H), 8.25 (dd, *J* = 4.67, 1.54 Hz, 1H), 8.00 (dd, *J* = 7.92, 1.54 Hz, 1H), 7.41 (s, 2H), 7.12 (t, *J* = 7.77 Hz, 2H), 7.06 – 7.00 (m, 3H), 6.58 (d, *J* = 7.30 Hz, 2H), 3.03 (t, *J* = 7.66 Hz, 2H), 1.72 (tt, *J* = 7.76, 6.44 Hz, 2H), 1.43 (h, *J* = 7.39 Hz, 2H), 0.94 (t, *J* = 7.37 Hz, 3H).

^{13}C{^{1}H} NMR (126 MHz, Acetone *d*-6) δ 151.4, 144.9, 142.6, 137.4, 128.5, 127.9, 124.1, 121.4, 120.9, 118.2, 115.8, 107.0, 36.5, 26.0, 23.3, 14.3.

Note: The carbon bearing boron was not observed due to quadrupolar relaxation.

^{11}B NMR (128 MHz, $CDCl_3$) δ 27.6.

IR (ATR, solid) ν_{max} 2924, 1601, 1586, 1543, 1489, 1416, 1375, 1333, 817, 758 cm^{-1}.

HRMS (ESI) *m/z*: [M + H]+ Calcd for $C_{21}H_{22}B_1N_4$ 341.1932; Found 341.1943.

1-(3-Butyl-2-(1H-naphtho[1,8-*de*][1,3,2]diazaborinin-2(3*H*)-yl)-1*H*-indol-1-yl)ethan-1-one (5a)

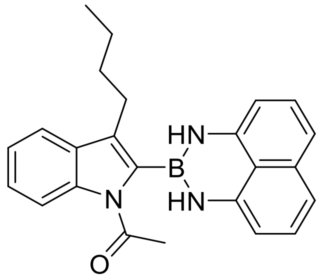

Prepared according to General procedure E from 2-(hex-1-yn-1-yl)-2,3-dihydro-1*H*-naphtho[1,8-*de*][1,3,2]diazaborinine (49.7 mg, 200 µmol, 1.00 Equiv.) and *N*-(2-iodophenyl)acetamide (62.4 mg, 240 µmol, 1.20 Equiv.). Purified by flash column chromatography (silica gel, 0 to 10% EtOAc in hexane) to yield the title compound as a white solid which quickly discolors (70.8 mg, 93%).

^{1}H NMR (400 MHz, $CDCl_3$) δ 7.92 (dt, *J* = 8.4, 0.9 Hz, 1H), 7.59 (ddd, *J* = 7.6, 1.5, 0.7 Hz, 1H), 7.43 – 7.28 (m, 2H), 7.13 (dd, *J* = 8.3, 7.2 Hz, 2H), 7.07 (dd, *J* = 8.4, 1.1 Hz, 2H), 6.35 (dd, *J* = 7.2, 1.1 Hz, 2H), 5.74 (s, 2H), 2.77 (m, 5H), 1.66 (p, *J* = 7.5 Hz, 2H), 1.39 (h, *J* = 7.3 Hz, 2H), 0.92 (t, *J* = 7.3 Hz, 3H).

^{13}C{^{1}H} NMR (101 MHz, $CDCl_3$) δ 169.5, 141.1, 136.9, 136.5, 132.3, 130.3, 127.7, 125.1, 123.1, 119.9, 119.8, 118.0, 114.9, 106.2, 33.1, 26.1, 25.4, 22.9, 14.1.

Note: The carbon bearing boron was not observed due to quadrupolar relaxation.

^{11}B NMR (128 MHz, $CDCl_3$) δ 28.8.

IR (ATR, film) ν_{max} 3392, 3375, 2951, 2926, 2866, 2852, 1672 cm^{-1}.

HRMS (ESI) *m/z*: [M + H]+ Calcd for $C_{24}H_{25}B_1N_3O_1$ 382.2085; Found 382.2085.

1-(3-Butyl-2-(1*H*-naphtho[1,8-*de*][1,3,2]diazaborinin-2(3*H*)-yl)-5-methyl-1*H*-indol-1-yl)ethan-1-one (5b)

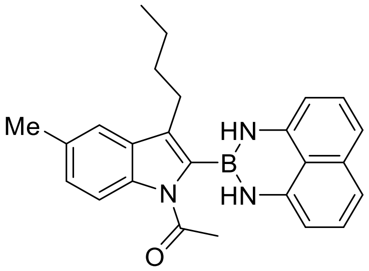

Prepared according to General procedure E from 2-(hex-1-yn-1-yl)-2,3-dihydro-1*H*-naphtho[1,8-*de*][1,3,2]diazaborinine (49.7 mg, 200 µmol, 1.00 Equiv.) and *N*-(2-iodo-4-methylphenyl)acetamide (65.6 mg, 240 µmol, 1.20 Equiv.). Purified by flash column chromatography (silica gel, 0 to 10% EtOAc in hexane) to yield the title compound as a white solid which quickly discolors black (75.1 mg, 94%).

^{1}H NMR (400 MHz, $CDCl_3$) δ 7.77 (d, *J* = 8.5 Hz, 1H), 7.50 – 7.32 (m, 1H), 7.20 – 7.09 (m, 3H), 7.06 (dd, *J* = 8.4, 1.1 Hz, 2H), 6.34 (dd, *J* = 7.2, 1.1 Hz, 2H), 5.73 (s, 2H), 2.85 – 2.67 (m, 5H), 2.49 (s, 3H), 1.65 (p, *J* = 7.5 Hz, 2H), 1.39 (h, *J* = 7.3 Hz, 2H), 0.92 (t, *J* = 7.3 Hz, 3H).

^{13}C{^{1}H} NMR (126 MHz, $CDCl_3$) δ 169.2, 141.0, 136.4, 135.0, 132.6, 132.4, 129.9, 127.6, 126.2, 119.8, 119.7, 117.8, 114.4, 106.0, 32.9, 25.8, 25.3, 22.8, 21.3, 14.0.

Note: The carbon bearing boron was not observed due to quadrupolar relaxation.

^{11}B NMR (128 MHz, $CDCl_3$) δ 28.8.

IR (ATR, film) ν_{max} 3275, 3062, 3037, 1703, 1566, 1519 cm^{-1}.

HRMS (ESI) *m*/*z*: [M + H]+ Calcd for $C_{25}H_{27}B_1N_3O_1$ 396.2242; Found 396.2248.

1-(3-Butyl-2-(1*H*-naphtho[1,8-*de*][1,3,2]diazaborinin-2(3*H*)-yl)-5-(fluoro)-1*H*-indol-1-yl)ethan-1-one (5c)

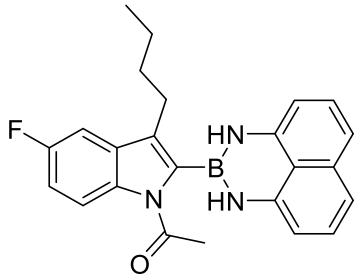

Prepared according to General procedure E from 2-(hex-1-yn-1-yl)-2,3-dihydro-1*H*-naphtho[1,8-*de*][1,3,2]diazaborinine (49.7 mg, 200 µmol, 1.00 Equiv.) and *N*-(2-iodo-4-fluorophenyl)acetamide (66.7 mg, 240 µmol, 1.20 Equiv.). Purified by flash column chromatography (silica gel, 0 to 10% EtOAc in hexane) to yield the title compound as a white solid which quickly discolors black (71.0 mg, 89%).

^{1}H NMR (400 MHz, $CDCl_3$) δ 7.95 (dd, J = 9.2, 4.3 Hz, 1H), 7.20 (d, J = 8.7 Hz, 1H), 7.17 – 7.11 (m, 2H), 7.10 – 6.99 (m, 3H), 6.36 (d, J = 7.1 Hz, 2H), 5.77 (s, 2H), 2.85 – 2.64 (m, 5H), 1.63 (p, J = 7.9 Hz, 2H), 1.38 (h, J = 7.5 Hz, 2H), 0.92 (t, J = 7.3 Hz, 3H).

^{13}C{^{1}H} NMR (101 MHz, $CDCl_3$) δ 169.3, 159.4 (d, J_{CF} = 240.6 Hz), 140.7, 136.4, 133.4, 133.0 (d, J_{CF} = 9.2 Hz), 133.0, 129.8 (d, J_{CF} = 3.6 Hz), 127.6, 119.7, 118.1, 116.1 (d, $J_{C\text{-}F}$ = 9.0 Hz), 112.7, 112.6 (d, J_{CF} = 25.1 Hz), 105.2 (d, J_{CF} = 23.2 Hz), 32.8, 25.9, 25.3, 22.7, 14.0.

Note: The carbon bearing boron was not observed due to quadrupolar relaxation.

^{19}F NMR (377 MHz, CDCl3) δ −120.0.

^{11}B NMR (128 MHz, $CDCl_3$) δ 28.3.

IR (ATR, film) ν_{max} 3538, 2941, 2919, 1620, 1560 cm^{-1}.

HRMS (ESI) *m*/*z*: [M + H]+ Calcd for $C_{24}H_{23}FB_1N_3O_1$ 400.1997; Found 400.1991.

1-(3-Butyl-2-(1*H*-naphtho[1,8-*de*][1,3,2]diazaborinin-2(3*H*)-yl)-5-(trifluoromethyl)-1*H*-indol-1-yl)ethan-1-one (5d)

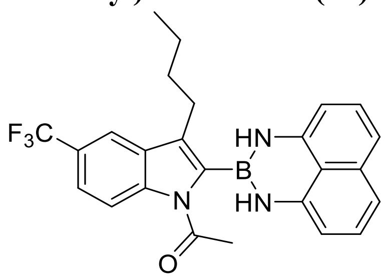

Prepared according to General procedure E from 2-(hex-1-yn-1-yl)-2,3-dihydro-1*H*-naphtho[1,8-*de*][1,3,2]diazaborinine (49.7 mg, 200 µmol, 1.00 Equiv.) and *N*-(2-iodo-4-trifluoromethylphenyl)acetamide (72.8 mg, 240 µmol, 1.20 Equiv.). Purified by flash column chromatography (silica gel, 0 to 10% EtOAc in hexane) to yield the title compound as a white solid which quickly discolors (65.5 mg, 73%).

^{1}H NMR (500 MHz, $CDCl_3$) δ 8.10 (d, J = 8.7 Hz, 1H), 7.90 – 7.76 (m, 1H), 7.58 (dd, J = 8.8, 1.8 Hz, 1H), 7.14 (dd, J = 8.3, 7.2 Hz, 2H), 7.09 (dd, J = 8.3, 1.1 Hz, 2H), 6.37 (dd, J = 7.2, 1.1 Hz, 2H), 5.79 (s, 2H), 2.84 – 2.77 (m, 5H), 1.66 (tt, J = 9.1, 6.9 Hz, 2H), 1.40 (h, J = 7.4 Hz, 2H), 0.93 (t, J = 7.3 Hz, 3H).

^{13}C{^{1}H} NMR (126 MHz, $CDCl_3$) δ 169.7, 140.7, 138.7, 136.5, 131.8, 130.2, 127.7, 125.5 (q, J_{CF} = 32.3 Hz) 124.7 (q, J_{CF} = 272.0 Hz) 121.9 (q, J_{CF} = 3.6 Hz), 119.9, 118.4, 117.0 (q, J_{CF} = 4.1 Hz), 115.5, 106.4, 33.1, 26.2, 25.3, 22.9, 14.1.

Note: The carbon bearing boron was not observed due to quadrupolar relaxation.

^{19}F NMR (377 MHz, $CDCl_3$) δ −61.0.

^{11}B NMR (128 MHz, $CDCl_3$) δ 28.3.

IR (ATR, film) ν_{max} 3441, 3387, 2954, 2927, 2856, 1687, 1627, 1598 cm^{-1}.

HRMS (ESI) *m/z*: [M + H]+ Calcd for $C_{25}H_{24}F_3B_1N_3O_1$ 450.1959; Found 450.1978.

1-(3-Butyl-2-(1*H*-naphtho[1,8-*de*][1,3,2]diazaborinin-2(3*H*)-yl)-5-chloro-1*H*-indol-1-yl)ethan-1-one (5e)

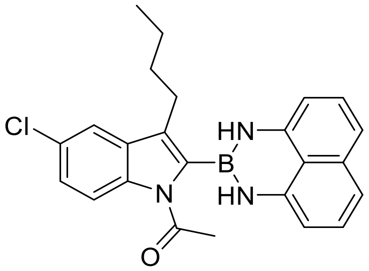

Prepared according to General procedure E from 2-(hex-1-yn-1-yl)-2,3-dihydro-1*H*-naphtho[1,8-*de*][1,3,2]diazaborinine (49.7 mg, 200 µmol, 1.00 Equiv.) and *N*-(2-iodo-4-chlorophenyl)acetamide (70.5 mg, 240 µmol, 1.20 Equiv.). Purified by flash column chromatography (silica gel, 0 to 10% EtOAc in hexane) to yield the title compound as a white solid which quickly discolors (70.5 mg, 85%).

^{1}H NMR (400 MHz, $CDCl_3$) δ 7.92 (d, J = 8.9 Hz, 1H), 7.52 (dd, J = 2.1, 0.5 Hz, 1H), 7.29 (dd, J = 8.8, 2.1 Hz, 1H), 7.17 – 7.04 (m, 4H), 6.36 (dd, J = 7.1, 1.2 Hz, 2H), 5.96 – 5.55 (m, 2H), 2.89 – 2.65 (m, 5H), 1.63 (p, J = 7.5 Hz, 2H), 1.38 (h, J = 7.3 Hz, 2H), 0.92 (t, J = 7.3 Hz, 3H).

^{13}C{^{1}H} NMR (101 MHz, $CDCl_3$) δ 169.5, 140.8, 136.5, 135.6, 133.4, 129.6, 128.9, 127.7, 125.1, 119.9, 119.4, 118.3, 116.2, 106.4, 33.0, 26.0, 25.4, 22.9, 14.1.

^{11}B NMR (128 MHz, $CDCl_3$) δ 28.5.

IR (ATR, film) ν_{max} 3385, 2953, 2926, 2854, 1681 cm^{-1}.

HRMS (ESI) *m/z*: [M + H]+ Calcd for $C_{24}H_{24}Cl_1B_1N_3O_1$ 416.1696; Found 416.1698.

Methyl-1-acetyl-3-butyl-2-(1*H*-naphtho[1,8-*de*][1,3,2]diazaborinin-2(3*H*)-yl)-1*H*-indole-5-carboxylate (5f)

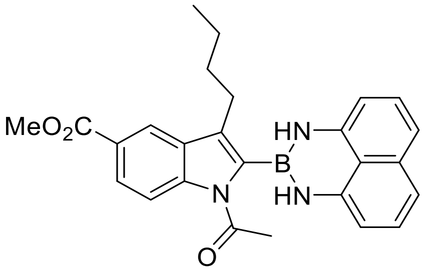

Prepared according to General procedure E from 2-(hex-1-yn-1-yl)-2,3-dihydro-1*H*-naphtho[1,8-*de*][1,3,2]diazaborinine (49.7 mg, 200 µmol, 1.00 Equiv.) and methyl 4-acetylamino-3-iodobenzoate (76.3 mg, 240 µmol, 1.20 Equiv.). Purified by flash column chromatography (silica gel, 10% EtOAc in hexane) to yield the title compound as a white solid which quickly discolors (44.7 mg, 51%).

^{1}H NMR (400 MHz, $CDCl_3$) δ 8.26 (dd, J = 1.7, 0.7 Hz, 1H), 8.00 (qd, J = 8.8, 1.2 Hz, 2H), 7.20 – 7.03 (m, 4H), 6.36 (dd, J = 7.1, 1.2 Hz, 2H), 5.78 (s, 2H), 3.97 (s, 3H), 2.97 – 2.52 (m, 5H), 1.66 (p, J = 7.5 Hz, 2H), 1.39 (h, J = 7.3 Hz, 2H), 0.92 (t, J = 7.3 Hz, 3H).

^{13}C{^{1}H} NMR (101 MHz, $CDCl_3$) δ 169.6, 167.5, 140.8, 139.7, 136.5, 132.0, 130.6, 127.7, 126.4, 125.1, 121.9, 119.9, 118.2, 114.7, 106.3, 52.3, 33.2, 26.2, 25.4, 22.9, 14.1.

Note: The carbon bearing boron was not observed due to quadrupolar relaxation.

^{11}B NMR (128 MHz, $CDCl_3$) δ 28.4.

IR (ATR, film) ν_{max} 3400, 3345, 2934, 2865, 1690, 1630, 1599 cm^{-1}.

HRMS (ESI) *m*/*z*: [M + H]+ Calcd for $C_{26}H_{27}B_1N_3O_3$ 440.2140; Found 440.2141.

1-(3-Butyl-2-(1*H*-naphtho[1,8-de][1,3,2]diazaborinin-2(3*H*)-yl)-6-chloro-1*H*-indol-1-yl)ethan-1-one (5g)

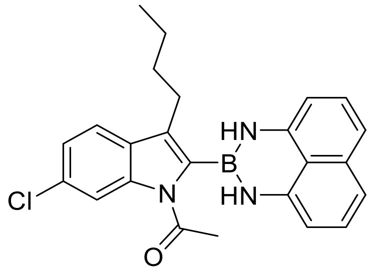

Prepared according to General procedure E from 2-(hex-1-yn-1-yl)-2,3-dihydro-1*H*-naphtho[1,8-*de*][1,3,2]diazaborinine (49.7 mg, 200 µmol, 1.00 Equiv.) *N*-(2-iodo-5-chlorophenyl)acetamide (70.5 mg, 240 µmol, 1.20 Equiv.). Purified by flash column chromatography (silica gel, 0 to 10% EtOAc in hexane) to yield the title compound as a white solid which quickly discolors (72.0 mg, 87%).

^{1}H NMR (500 MHz, $CDCl_3$) δ 8.03 (d, *J* = 1.8 Hz, 1H), 7.47 (d, *J* = 8.3 Hz, 1H), 7.30 – 7.21 (m, 1H, *overlaps with* $CDCl_3$ *signal*), 7.14 (dd, *J* = 8.3, 7.2 Hz, 2H), 7.08 (dd, *J* = 8.4, 1.1 Hz, 2H), 6.36 (dd, *J* = 7.2, 1.1 Hz, 2H), 5.77 (s, 2H), 2.82 – 2.69 (m, 5H), 1.63 (p, *J* = 7.6 Hz, 2H), 1.37 (h, *J* = 7.3 Hz, 2H), 0.91 (t, *J* = 7.3 Hz, 3H).

^{13}C{^{1}H} NMR (126 MHz, $CDCl_3$) δ 169.5, 140.8, 137.6, 136.5, 131.2, 130.5, 130.0, 127.7, 123.7, 120.4, 119.9, 118.3, 115.6, 106.4, 33.0, 26.1, 25.4, 22.9, 14.1.

Note: The carbon bearing boron was not observed due to quadrupolar relaxation.

^{11}B NMR (128 MHz, $CDCl_3$) δ 28.3.

IR (ATR, film) ν_{max} 3406, 3375, 2924, 2856, 1665, 1627, 1601, 1550 cm^{-1}.

HRMS (ESI) *m/z*: [M + H]+ Calcd for $C_{24}H_{24}Cl_1B_1N_3O_1$ 416.1696; Found 416.1698.

1-(7-Butyl-6-(1*H*-naphtho[1,8-*de*][1,3,2]diazaborinin-2(3*H*)-yl)-5*H*-[1,3]dioxolo[4,5-f]indol-5-yl)ethan-1-one (5h)

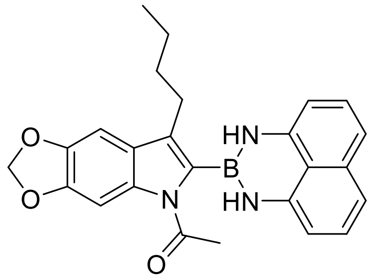

Prepared according to General procedure E from 2-(hex-1-yn-1-yl)-2,3-dihydro-1*H*-naphtho[1,8-*de*][1,3,2]diazaborinine (49.7 mg, 200 µmol, 1.00 Equiv.) *N*-(6-iodo-1,3-benzodioxol-5-yl)acetamide (80.0 mg, 240 µmol, 1.20 Equiv.). Purified by flash column chromatography (silica gel, 0 to 10% EtOAc in hexane) to yield the title compound as a white solid (77.3 mg, 91%).

^{1}H NMR (400 MHz, $CDCl_3$) δ 7.63 (s, 1H), 7.13 (dd, *J* = 8.3, 7.1 Hz, 2H), 7.07 (dd, *J* = 8.4, 1.2 Hz, 2H), 6.91 (s, 1H), 6.35 (dd, *J* = 7.2, 1.2 Hz, 2H), 6.02 (s, 2H), 5.79 (s, 2H), 2.79 – 2.59 (m, 5H), 1.72 – 1.52 (m, 2H, *overlaps with H_2O signal*), 1.37 (h, *J* = 7.3 Hz, 2H), 0.91 (t, *J* = 7.3 Hz, 3H).

$^{13}C\{^1H\}$ NMR (101 MHz, $CDCl_3$) δ 169.7, 146.9, 144.8, 140.9, 136.5, 132.3, 130.6, 127.7, 126.0, 119.9, 118.2, 106.3, 101.6, 98.4, 97.5, 33.0, 26.1, 25.6, 22.9, 14.1.

Note: The carbon bearing boron was not observed due to quadrupolar relaxation.

^{11}B NMR (128 MHz, $CDCl_3$) δ 28.5.

IR (ATR, film) ν_{max} 3421, 3410, 2531, 1683, 1598, 1570, 1538 cm^{-1}.

HRMS (ESI) *m/z*: [M + H]+ Calcd for $C_{25}H_{25}B_1N_3O_3$ 426.1984; Found 426.1987.

1-(3-(2-(Benzyloxy)ethyl)-2-(1*H*-naphtho[1,8-*de*][1,3,2]diazaborinin-2(3*H*)-yl)-1*H*-indol-1-yl)ethan-1-one (5i)

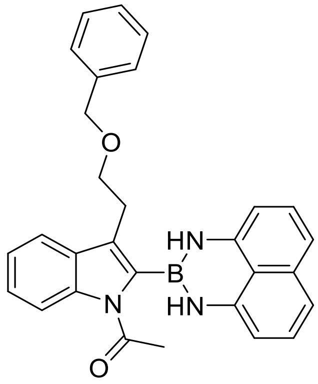

Prepared according to General procedure E from 2-(4-(Benzyloxy)but-1-yn-1-yl)-2,3-dihydro-1H-naphtho[1,8-de][1,3,2]diazaborinine (65.2 mg, 200 µmol, 1.00 Equiv.) and *N*-(2-iodophenyl)acetamide (62.4 mg, 240 µmol, 1.20 Equiv.). Purified by flash column chromatography (silica gel, 0 to 10% EtOAc in hexane) to yield the title compound as a white solid which quickly discolors (76.2 mg, 83%).

^{1}H NMR (400 MHz, $CDCl_3$) δ 8.13 (d, *J* = 8.3 Hz, 1H), 7.53 (d, *J* = 6.7 Hz, 1H), 7.38 (ddd, *J* = 8.5, 7.2, 1.3 Hz, 1H), 7.34 – 7.20 (m, 7H), 7.13 – 6.96 (m, 4H), 6.18 (s, 2H), 6.03 (dd, *J* = 6.8, 1.6 Hz, 2H), 4.48 (s, 2H), 3.81 (t, *J* = 5.8 Hz, 2H), 3.09 (t, *J* = 5.8 Hz, 2H), 2.77 (s, 3H).

^{13}C{^{1}H} NMR (101 MHz, $CDCl_3$) δ 170.2, 141.0, 138.1, 137.8, 136.5, 131.4, 128.7, 128.2, 128.1, 127.6, 127.5, 125.4, 123.3, 119.9, 119.2, 117.8, 115.6, 106.2, 73.6, 69.8, 26.6, 26.4.

Note: The carbon bearing boron was not observed due to quadrupolar relaxation.

^{11}B NMR (128 MHz, $CDCl_3$) δ 27.4.

IR (ATR, film) ν_{max} 3342, 2899, 2862, 2792, 1681, 1627, 1600 cm^{-1}.

HRMS (ESI) *m*/*z*: [M + H]+ Calcd for $C_{29}H_{27}B_1N_3O_2$ 460.2191; Found 460.2197.

1-(3-(Cyclohex-1-en-1-yl)-2-(1*H*-naphtho[1,8-*de*][1,3,2]diazaborinin-2(3*H*)-yl)-1*H*-indol-1-yl)ethan-1-one (5j)

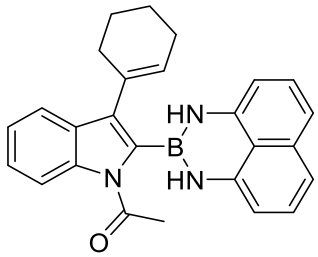

Prepared according to General procedure E from 2-(Cyclohex-1-en-1-ylethynyl)-2,3-dihydro-1H-naphtho[1,8-de][1,3,2]diazaborinine (54.4 mg, 200 µmol, 1.00 Equiv.) and *N*-(2-iodophenyl)acetamide (62.4 mg, 240 µmol, 1.20 Equiv.). Purified by flash column chromatography (silica gel, 0 to 10% EtOAc in hexane) to yield the title compound as a white solid which quickly discolors (65.6 mg, 81%).

^{1}H NMR (400 MHz, $CDCl_3$) δ 7.93 (d, J = 8.3 Hz, 1H), 7.63 (ddd, J = 7.8, 1.4, 0.7 Hz, 1H), 7.36 (td, J = 7.8, 1.5 Hz, 1H), 7.29 (d, J = 7.3 Hz, 1H), 7.19 – 7.09 (m, 2H), 7.05 (d, J = 7.3 Hz, 2H), 6.33 (dd, J = 7.2, 1.1 Hz, 2H), 6.13 – 5.91 (m, 1H), 5.75 (s, 2H), 2.79 (s, 3H), 2.45 – 2.34 (m, 2H), 2.28 – 2.14 (m, 2H), 1.86 – 1.59 (m, 4H).

^{13}C{^{1}H} NMR (101 MHz, $CDCl_3$) δ 170.0, 141.2, 136.8, 136.5, 134.1, 131.6, 131.0, 128.8, 127.7, 125.3, 123.3, 121.0, 119.8, 117.8, 114.8, 106.1, 30.5, 26.4, 25.8, 23.2, 22.2.

Note: The carbon bearing boron was not observed due to quadrupolar relaxation.

^{11}B NMR (128 MHz, $CDCl_3$) δ 29.3.

IR (ATR, film) ν_{max} 3434, 2922, 2852, 2831, 1691, 1625, 1599 cm^{-1}.

HRMS (ESI) *m/z*: [M + H]+ Calcd for $C_{26}H_{25}B_1N_3O_1$ 406.2085; Found 406.2079.

1-(3-(Cyclohex-1-en-1-yl)-2-(1*H*-naphtho[1,8-*de*][1,3,2]diazaborinin-2(*3H*)-yl)-4-methyl-*1H*-indol-1-yl)ethan-1-one (5k)

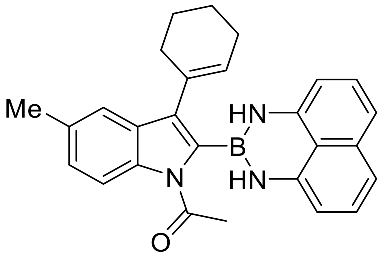

Prepared according to General procedure E from 2-(Cyclohex-1-en-1-ylethynyl)-2,3-dihydro-1H-naphtho[1,8-de][1,3,2]diazaborinine (54.4 mg, 200 µmol, 1.00 Equiv.) and *N*-(2-iodo-4-methylphenyl)acetamide (65.6 mg, 240 µmol, 1.20 Equiv.). Purified by flash column chromatography (silica gel, 0 to 10% EtOAc in hexane) to yield the title compound as a white solid which quickly discolors (65.4 mg, 78%).

^{1}H NMR (400 MHz, $CDCl_3$) δ 7.78 (d, *J* = 8.4 Hz, 1H), 7.39 (dt, *J* = 1.6, 0.8 Hz, 1H), 7.16 (ddd, *J* = 8.5, 1.8, 0.6 Hz, 1H), 7.12 (dd, *J* = 8.3, 7.2 Hz, 2H), 7.05 (dd, *J* = 8.4, 1.1 Hz, 2H), 6.32 (dd, *J* = 7.3, 1.1 Hz, 2H), 5.93 (tt, *J* = 3.9, 1.8 Hz, 1H), 5.75 (s, 2H), 2.76 (s, 3H), 2.47 (s, 3H), 2.40 – 2.34 (m, 2H), 2.25 – 2.15 (m, 2H), 1.89 – 1.59 (m, 4H).

^{13}C{^{1}H} NMR (101 MHz, $CDCl_3$) δ 169.8, 141.2, 136.5, 135.0, 133.9, 132.9, 131.9, 131.1, 128.6, 127.7, 126.5, 120.8, 119.8, 117.8, 114.5, 106.1, 30.5, 26.3, 25.8, 23.2, 22.3, 21.4.

Note: The carbon bearing boron was not observed due to quadrupolar relaxation.

^{11}B NMR (128 MHz, $CDCl_3$) δ 29.1.

IR (ATR, film) ν_{max} 3420, 3410, 3050, 2957, 2949, 1681 cm^{-1}.

HRMS (ESI) *m*/*z*: [M + H]+ Calcd for $C_{27}H_{27}B_1N_3O_1$ 420.2247; Found 420.2244.

1-(3-(Phenyl)-2-(1*H*-naphtho[1,8-*de*][1,3,2]diazaborinin-2(3*H*)-yl)-1*H*-indol-1-yl)ethan-1-one (5l)

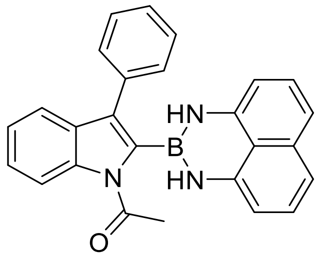

Prepared according to General procedure E from 2-(Phenylethynyl)-2,3-dihydro-1H-naphtho[1,8-de][1,3,2]diazaborinine (53.5 mg, 200 µmol, 1.00 Equiv.) and *N*-(2-iodophenyl)acetamide (62.4 mg, 240 µmol, 1.20 Equiv.). Purified by flash column chromatography (silica gel, 0 to 10% EtOAc in hexane) to yield the title compound as a white solid which quickly discolors (35.2 mg, 44%).

^{1}H NMR (400 MHz, $CDCl_3$) δ 7.98 (dt, *J* = 8.4, 0.9 Hz, 1H), 7.69 (ddd, *J* = 7.9, 1.3, 0.7 Hz, 1H), 7.65 – 7.58 (m, 2H), 7.48 – 7.37 (m, 3H), 7.36 – 7.28 (m, 2H), 7.08 (dd, *J* = 8.3, 7.1 Hz, 2H), 7.03 (dd, *J* = 8.4, 1.2 Hz, 2H), 6.26 (dd, *J* = 7.1, 1.2 Hz, 2H), 5.70 (s, 2H), 2.84 (s, 3H).

^{13}C{^{1}H} NMR (101 MHz, $CDCl_3$) δ 170.0, 140.9, 136.9, 136.4, 133.6, 131.5, 131.0, 129.3, 128.9, 127.7, 127.6, 125.6, 123.7, 120.8, 119.7, 117.9, 114.8, 106.2, 26.4.

Note: The carbon bearing boron was not observed due to quadrupolar relaxation.

^{11}B NMR (128 MHz, $CDCl_3$) δ 28.9.

IR (ATR, film) ν_{max} 3396, 2923, 2852, 2833, 1702, 1683, 1597 cm^{-1}.

HRMS (ESI) *m*/*z*: [M + H]+ Calcd for $C_{26}H_{21}B_{1}N_{3}O_{1}$ 402.1772; Found 402.1773.

6. Scale-up Reaction

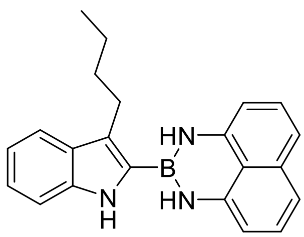

A flame-dried Schlenk flask was charged from 2-(hex-1-yn-1-yl)-2,3-dihydro-1*H*-naphtho[1,8-*de*][1,3,2]diazaborinine (500 mg, 2.00 mmol, 2.00 Equiv.), Pd(dtbpf)Cl_2 (61 mg, 0.1 mmol), 2-iodoaniline (437 mg, 1.00 mmol, 1.00 Equiv.) and NaOAc (205 mg, 2.50 mmol). The flask was sealed, evacuated and backfilled with N_2. Anhydrous DMSO (10.0 mL) was added and the mixture was stirred at 80 ºC for 15 hours. After this time, the reaction mixture was allowed to cool to room temperature before 10% aq. LiCl (20.0 mL) was added and the solution was extracted with EtOAc (3 × 10.0 mL). The combined organic extracts were washed with 10% aq. LiCl (2 × 20.0 mL), dried over Na_2SO_4 before being filtered and concentrated under reduced pressure. The crude product was then purified *via* flash column chromatography to afford 2-(3-butyl-1*H*-indol-2-yl)-2,3-dihydro-1*H*-naphtho[1,8-*de*][1,3,2]diazaborinine as a white solid which quickly discolors (299 mg, 89%).

7. Alkaloid Synthesis

2-Chloro-4-methoxyquinazoline (6)

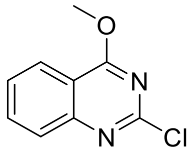

A flame-dried Schlenk flask was charged with 2,4-dichloroquinazoline (400 mg, 2.00 mmol, 1.00 Equiv.). The flask was sealed, evacuated and backfilled with N_2 before anhydrous MeOH (5.00 mL) was added. The flask was then unsealed before NaOMe (104 mg, 2.00 mmol, 1.00 Equiv.) was added in one portion under a flow of N_2 and the flask resealed. The reaction was stirred for 15 hours at room temperature. After this time, the mixture was concentrated under reduced pressure to afford an off-white solid. This solid was suspended in CH_2Cl_2 before being filtered through a plug of silica and concentrated under reduced pressure to afford the title compound as an off-white solid (384 mg, 99%). Spectral data is in agreement with literature reports.[9]

^{1}H NMR (400 MHz, $CDCl_3$) δ 8.12 (d, *J* = 8.9 Hz, 1H), 7.85 (m, 2H), 7.55 (t, *J* = 8.1 Hz, 1H), 4.21 (s, 3H).

2-(4-Methoxy-2-quinazolinyl)-1*H*-indole-3-ethanol (7)

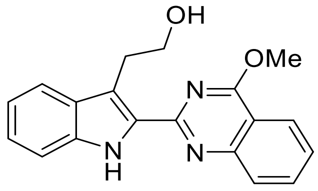

To a flame-dried Schlenk flask under an N_2 atmosphere was added $Pd_2(dba)_3$•$CHCl_3$ (5.2 mg, 5.0 µmol, 5 mol%), XPhos (9.5 mg, 20 µmol, 20 mol%), **4m** (44 mg, 0.10 mmol, 1.00 Equiv.), 2-chloro-4-methoxyquinazoline (29.1 mg, 0.15 mmol, 1.50 Equiv.) and anhydrous 1,4-dioxane (1.00 mL). The mixture was stirred for 30 minutes at 90 ˚C after which time *t*BuOK (1 M in THF, 150 µL, 0.15 mmol, 1.50 Equiv.) was added dropwise. The mixture was stirred for 3 hours at 90 ˚C before being quenched *via* addition of brine (5.00 mL) and extracted with EtOAc (3 × 5.00 mL). The combined organic extracts were dried over Na_2SO_4, filtered and concentrated under reduced pressure to afford a brown oil. The oil was redissolved in CH_2Cl_2 (5.00 mL) and filtered through celite before again being concentrated under reduced pressure to afford a brown oil. The residue was then dissolved in THF (1.00 mL) before TBAF (1 M in THF, 150 µL, 1.50 Equiv.) was added and the mixture stirred for 1 hour at room temperature being quenched *via* addition of brine (5.00 mL) and extracted with EtOAc (3 × 5.00 mL). The combined organic extracts were dried over Na_2SO_4, filtered and concentrated under reduced pressure. The crude product was then purified by flash column chromatography (silica gel, 0 to 5% acetone in hexane) to afford the title compound as an orange solid. (22.7 mg, 71%). Spectral data is in agreement with literature reports.[10]

^{1}H NMR (400 MHz, $CDCl_3$) δ 9.49 (s, 1H), 8.22 – 8.13 (m, 1H), 8.00 (s, 1H), 7.86 (t, *J* = 7.4 Hz, 1H), 7.73 (d, *J* = 8.1 Hz, 1H), 7.55 (t, *J* = 7.6 Hz, 1H), 7.49 (d, *J* = 8.2 Hz, 1H), 7.33 (t, *J* = 7.0 Hz, 1H), 7.18 (t, *J* = 7.0 Hz, 1H), 4.35 (s, 3H), 4.15 (t, *J* = 6.0 Hz, 2H), 3.72 (t, *J* = 6.0 Hz, 2H).

Rutaecarpine (8)

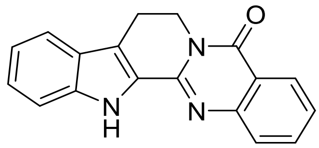

Prepared according to previously reported procedures.[10] To an oven-dried microwave vial was added added 2-(4-methoxy-2-quinazolinyl)-1*H*-indole-3-ethanol (10.0 mg, 31 µmol, 1.00 Equiv.), *n*BuOH (1.00 mL) and aq. HCl (6 M, 25 µL, 150 µmol, 5.00 Equiv.) and the bright yellow mixture was heated to 110 ˚C in a sand bath for 10 days. The mixture was allowed to cool to room temperature before sat. aq. $NaHCO_3$ was added to adjust the solution pH to 8. The mixture was extracted with EtOAc (3 × 5.00 mL), dried over Na_2SO_4, filtered and concentrated under reduced pressure. The crude product was then purified by flash column chromatography (10 to 30% EtOAc in hexane) to afford the title compound as an off-white solid (8.1 mg, 91%). Spectral data is in accordance with literature reports.[11]

^{1}H NMR (400 MHz, $CDCl_3$) δ 9.32 (s, 1H), 8.30 (d, *J* = 7.6 Hz, 1H), 7.77 – 7.73 (m, 2H), 7.61 (d, *J* = 8.1 Hz, 1H), 7.46 (m, 2H), 7.34 (ddd, *J* = 8.2, 7.0, 1.1 Hz, 1H), 7.18 (ddd, *J* = 8.1, 7.0, 1.0 Hz, 1H), 4.21 (t, *J* = 5.2 Hz, 2H), 3.33 (t, *J* = 5.3 Hz, 2H).

6*H*-Indolo[2′,3′:3,4]pyrido[1,2-*a*]quinazolin-5-ium,7,12-dihydro-14-methoxy-chloride (9')

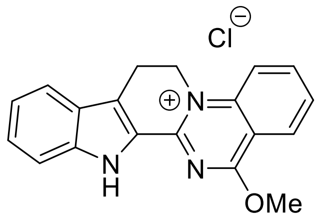

Prepared according to previously reported procedures.[10] To a flame-dried Schlenk flask under an N_2 atmosphere was added 2-(4-methoxy-2-quinazolinyl)-1*H*-indole-3-ethanol (10.0 mg, 31 µmol, 1.00 Equiv.) and CH_2Cl_2 (500 µL). DMAP (0.36 mg, 3 µmol, 10 mol%), pyridine (3.6 µL, 4.5 µmol, 1.50 Equiv.) and toluenesulfonyl chloride (7.6 mg, 40 µmol, 1.30 Equiv.) were sequentially added at room temperature and the mixture was stirred for 4 hours at room temperature over which time a yellow precipitate had formed. The precipitate was collected by filtration, washed with CH_2Cl_2 (1.00 mL) and dried under vacuum to afford the title compound (10.0 mg, 99%). Spectral data is in agreement with literature reports.[10]

^{1}H NMR (400 MHz, *d*6-DMSO) δ 12.43 (s, 1H), 8.46 (d, *J* = 8.5 Hz, 1H), 8.39 (d, *J* = 7.6 Hz, 1H), 8.29 (t, *J* = 7.8 Hz, 1H), 7.87 (m, 2H), 7.62 (d, *J* = 8.8 Hz, 1H), 7.47 (t, *J* = 7.0 Hz, 1H), 7.22 (t, *J* = 6.9 Hz, 1H), 5.04 (t, *J* = 7.8 Hz, 2H), 4.54 (s, 3H), 3.54 (t, *J* = 7.7 Hz, 2H).

Indolo[2′,3′:3,4]pyrido[1,2-*a*]quinazolin-14(6*H*)-one, 7,12-dihydro (9)

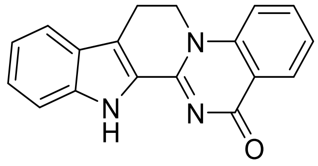

Prepared according to previously reported procedures.[10] To an oven-dried microwave vial was added 6*H*-indolo[2′,3′:3,4]pyrido[1,2-*a*]quinazolin-5-ium, 7,12-dihydro-14-methoxy-chloride (10.0 mg, 31 µmol, 1.00 Equiv.) and DMSO (500 µL) The vial was then capped and the mixture heated to 140 ˚C in a sand bath for 20 minutes. Removal of solvent under high vacuum afforded the title compound as a pale-yellow solid (9.7 mg, 99%). Spectral data is in agreement with literature reports. [10]

^{1}H NMR (400 MHz, *d*6-DMSO) δ 12.04 (s, 1H), 8.15 (dd, *J* = 7.9, 1.6 Hz, 1H), 7.95 (d, *J* = 7.6 Hz, 1H), 7.84 (ddd, *J* = 8.7, 7.1, 1.7 Hz, 1H), 7.74 – 7.68 (m, 1H), 7.53 – 7.44 (m, 2H), 7.29 (ddd, *J* = 8.2, 6.9, 1.2 Hz, 1H), 7.12 (ddd, *J* = 8.1, 6.9, 1.0 Hz, 1H), 4.60 (t, *J* = 7.2 Hz, 2H). *Note: Second CH_2 not observable due to overlap with H_2O signal.*

8. X-ray Crystallography

X-ray diffraction data for compound **4o** were collected at 100 K using a Rigaku MM-007HF High Brilliance RA generator/confocal optics with XtaLAB P200 diffractometer [Cu Kα radiation (λ = 1.54187 Å)]. Data for all compounds analysed were collected (using a calculated strategy) and processed (including correction for Lorentz, polarization and absorption) using CrysAlisPro.[12] Structures were solved by dual-space methods (SHELXT[13]) and refined by full-matrix least-squares against F2 (SHELXL-2019/3[14]). Non-hydrogen atoms were refined anisotropically, and hydrogen atoms were refined using a riding model except for the hydrogen atoms on N1, N3, and N15 which were located from the difference Fourier map and refined isotropically subject to a distance restraint. Selected crystallographic data: $C_{21}H_{21}BN_4$, M = 340.23, monoclinic, a = 18.8255(8), b = 5.20201(19), c = 18.5130(9) Å, β = 106.396(5)°, U = 1739.26(14) Å3, T = 100 K, space group P21/c (no. 14), Z = 4, 18673 reflections measured, 3538 unique (Rint = 0.0869), which were used in all calculations. The final R1 [I > 2σ(I)] was 0.0881 and wR2 (all data) was 0.2461. The data showed twinning with a second component located at [1 0 0.574 0 -1 0 0 0 -1] with HKL5 generated by the TwinRotMat routine in PLATON[15] to account for contribution to diffraction pattern from minor component. All calculations except TwinRotMat were performed using the Olex2[16] interface. CCDC 2415036 contains the supplementary crystallographic data for this paper. These data can be obtained free of charge from The Cambridge Crystallographic Data Centre via www.ccdc.cam.ac.uk/structures.

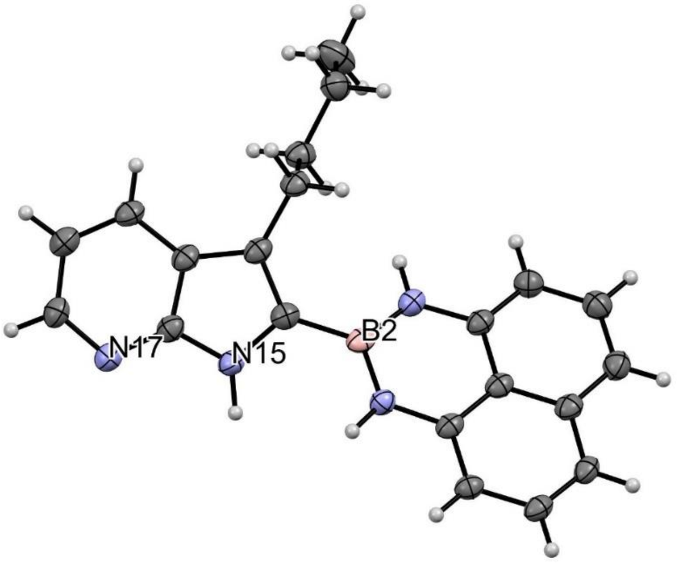

Figure S1. SCXRD Structure of 4o. Ellipsoid contour probability levels set at 50%.

9. Bibliography

(1) Bell, G. E.; Fyfe, J. W. B.; Israel, E. M.; Slawin, A. M. Z.; Campbell, M.; Watson, A. J. B. Synthesis of 2-BMIDA Indoles via Heteroannulation: Applications in Drug Scaffold and Natural Product Synthesis. *Org. Lett.* **2022**, *24*, 3024-3027.

(2) Wu, S.; Hu, W.-Y.; Zhang, S.-L. Potassium carbonate-mediated tandem C–S and C–N coupling reaction for the synthesis of phenothiazines under transition-metal-free and ligand-free conditions. *RSC Advances* **2016**, *6*, 24257-24260.

(3) Chaisan, N.; Kaewsri, W.; Thongsornkleeb, C.; Tummatorn, J.; Ruchirawat, S. PtCl4-catalyzed cyclization of N-acetyl-2-alkynylanilines: A mild and efficient synthesis of N-acetyl-2-substituted indoles. *Tetrahedron Letters* **2018**, *59*, 675-680.

(4) Melot, R.; Saiegh, T. J.; Fürstner, A. Regioselective trans-Hydrostannation of Boron-Capped Alkynes. *Chem. Eur. J.* **2021**, *27*, 17002-17011.

(5) Halford-McGuff, J. M.; Cordes, D. B.; Watson, A. J. B. Synthesis of complex aryl MIDA boronates by Rh-catalyzed [2+2+2] cycloaddition. *Chem. Commun.* **2023**, *59*, 7759-7762.

(6) Tani, T.; Sawatsugawa, Y.; Sano, Y.; Hirataka, Y.; Takahashi, N.; Hashimoto, S.; Sugiura, T.; Tsuchimoto, T. Alkynyl−B(dan)s in Various Palladium-Catalyzed Carbon−Carbon Bond-Forming Reactions Leading to Internal Alkynes, 1,4-Enynes, Ynones, and Multiply Substituted Alkenes. *Adv. Synth. Catal.* **2019**, *361*, 1815-1834.

(7) Li, J.; Tanaka, H.; Imagawa, T.; Tsushima, T.; Nakamoto, M.; Tan, J.; Yoshida, H. Ethynyl-B(dan) in [3+2] Cycloaddition and Larock Indole Synthesis: Synthesis of Stable Boron-Containing Heteroaromatic Compounds. *Chem. Eur. J.* **2024**, *30*, e202303403.

(8) Tsuchimoto, T.; Utsugi, H.; Sugiura, T.; Horio, S. Alkynylboranes: A Practical Approach by Zinc-Catalyzed Dehydrogenative Coupling of Terminal Alkynes with 1,8-Naphthalenediaminatoborane. *Adv. Synth. Catal.* **2015**, *357*, 77-82.

(9) Kazemi, S. S.; Keivanloo, A.; Nasr-Isfahani, H.; Bamoniri, A. Synthesis of novel 1,5-disubstituted pyrrolo[1,2-a]quinazolines and their evaluation for anti-bacterial and anti-oxidant activities. *RSC Advances* **2016**, *6*, 92663-9266.

(10) Pan, X.; Bannister, T. D. Sequential Sonagashira and Larock Indole Synthesis Reactions in a General Strategy To Prepare Biologically Active β-Carboline-Containing Alkaloids. *Org. Lett.* **2014**, *16*, 6124-6127.

(11) Tseng, M.-C.; Cheng, H.-T.; Shen, M.-J.; Chu, Y.-H. Bicyclic 1,2,3-Triazolium Ionic Liquids: Synthesis, Characterization, and Application to Rutaecarpine Synthesis. *Org. Lett.* **2011**, *13*, 4434-4437.

12. CrysAlisPro v1.171.43.109a Rigaku Oxford Diffraction, Rigaku Corporation, Tokyo, Japan, 2023.

13. Sheldrick, G. M. SHELXT – Integrated space-group and crystal structure determination. Acta Crystallogr., Sect. A: Found. Adv. 2015, 71, 3-8. doi:

14. Sheldrick, G. M. Crystal structure refinement with SHELXL. Acta Crystallogr., Sect. C: Struct. Chem. 2015, 71, 3-8.

15. Spek, A. L. Structure validation in chemical crystallography. Acta Crystallogr. Sect D: Biol. Crystallogr. 2009, 65, 148-155.

16. Dolomanov, O. V.; Bourhis, L. J.; Gildea, R. J.; Howard, J. A. K.; Puschmann, H. OLEX2: a complete structure solution, refinement and analysis program. J. Appl. Crystallogr. 2009, 42, 339-341.

Copies of NMR Spectra

(3f) ^{1}H NMR (500 MHz, $CDCl_3$):

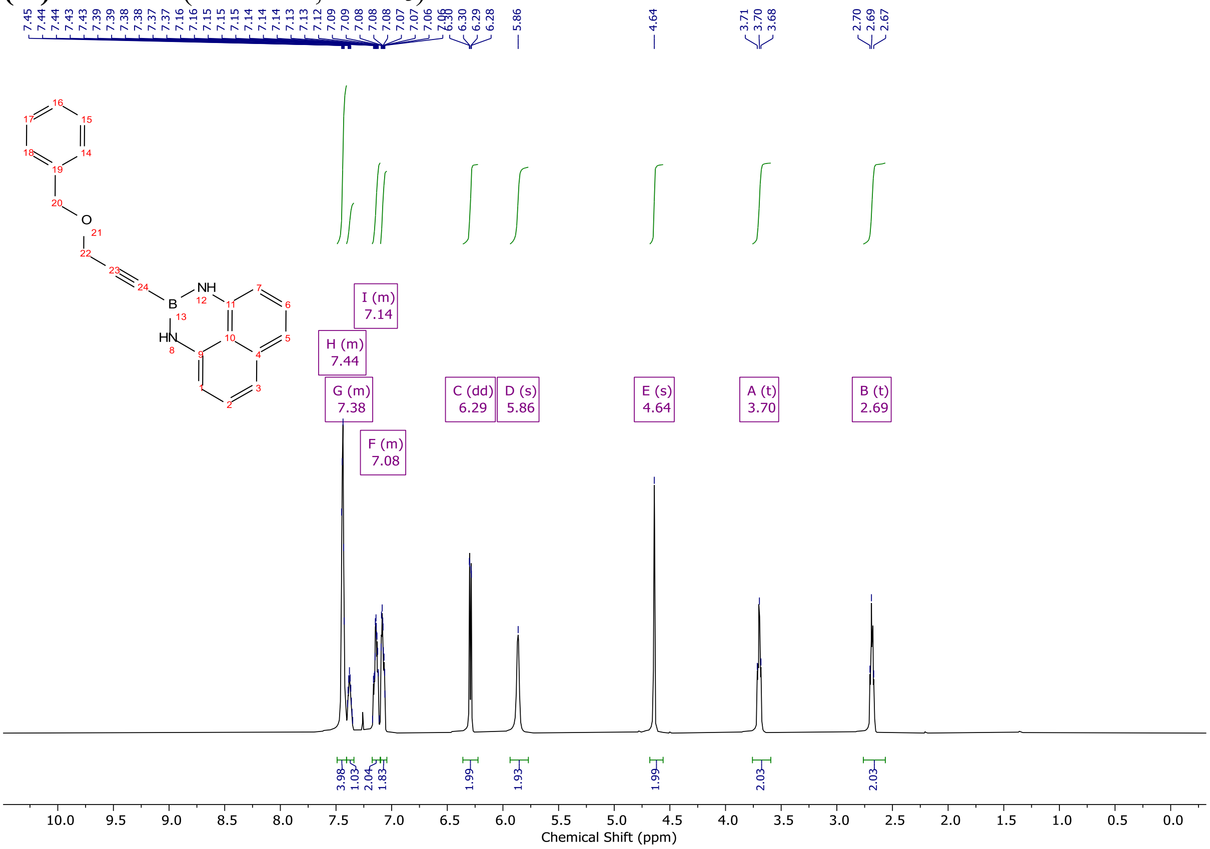

^{13}C{^{1}H} NMR (126 MHz, $CDCl_3$):

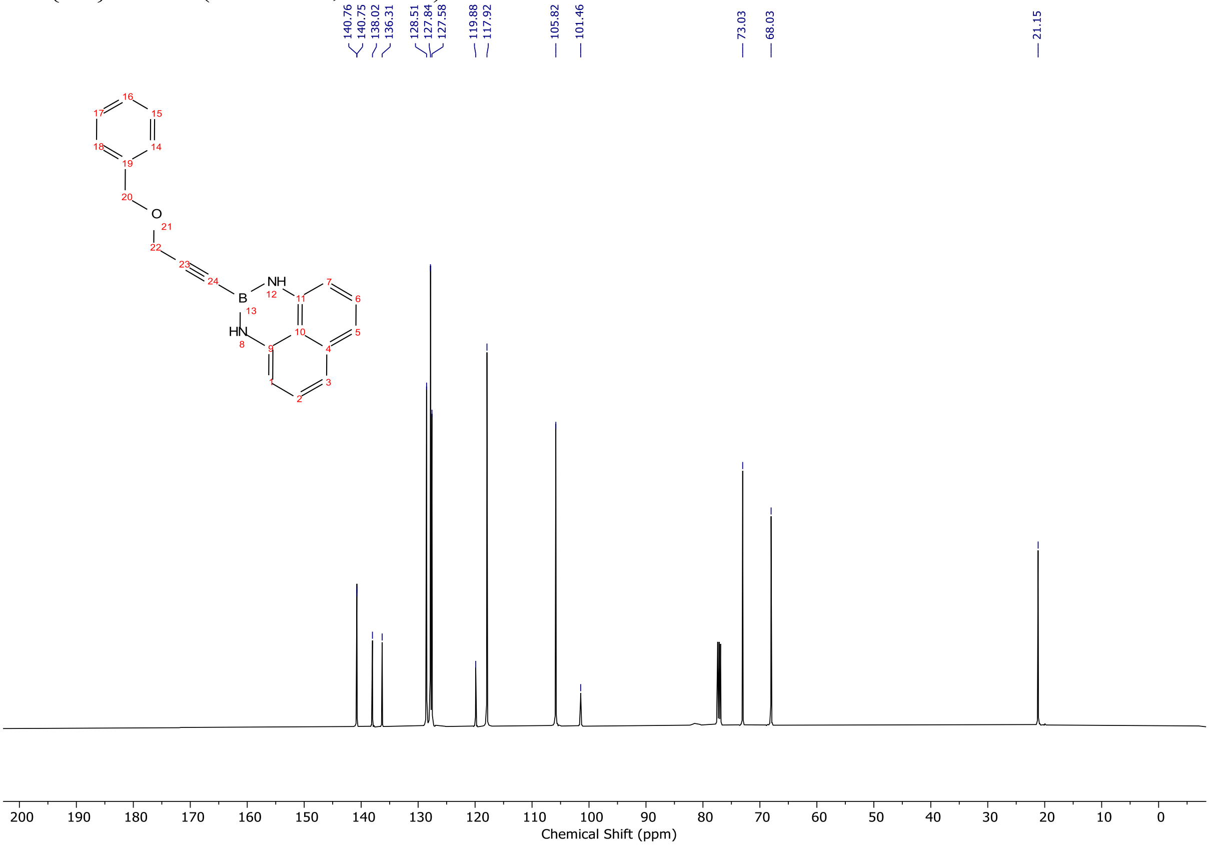

(4a) ^{1}H NMR (400 MHz, $CDCl_3$):

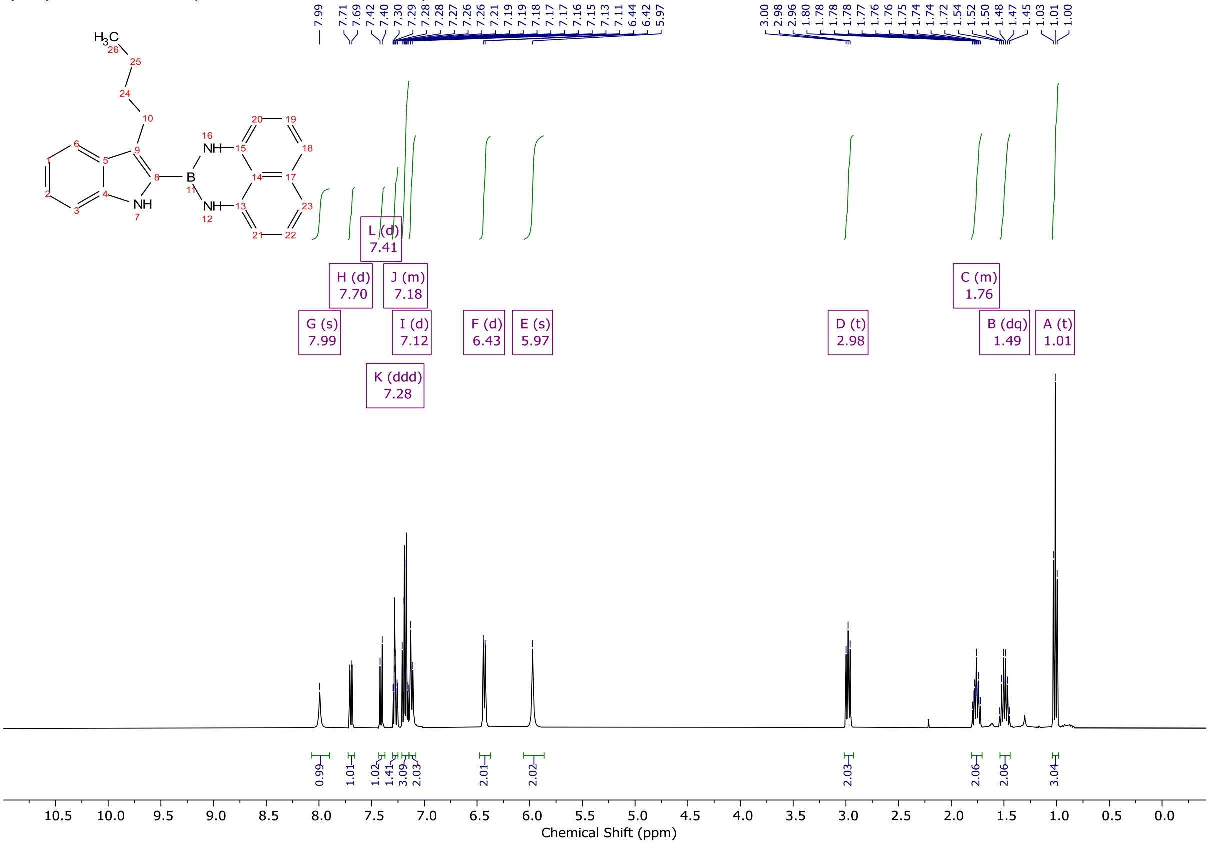

^{13}C DEPTQ (101 MHz, $CDCl_3$):

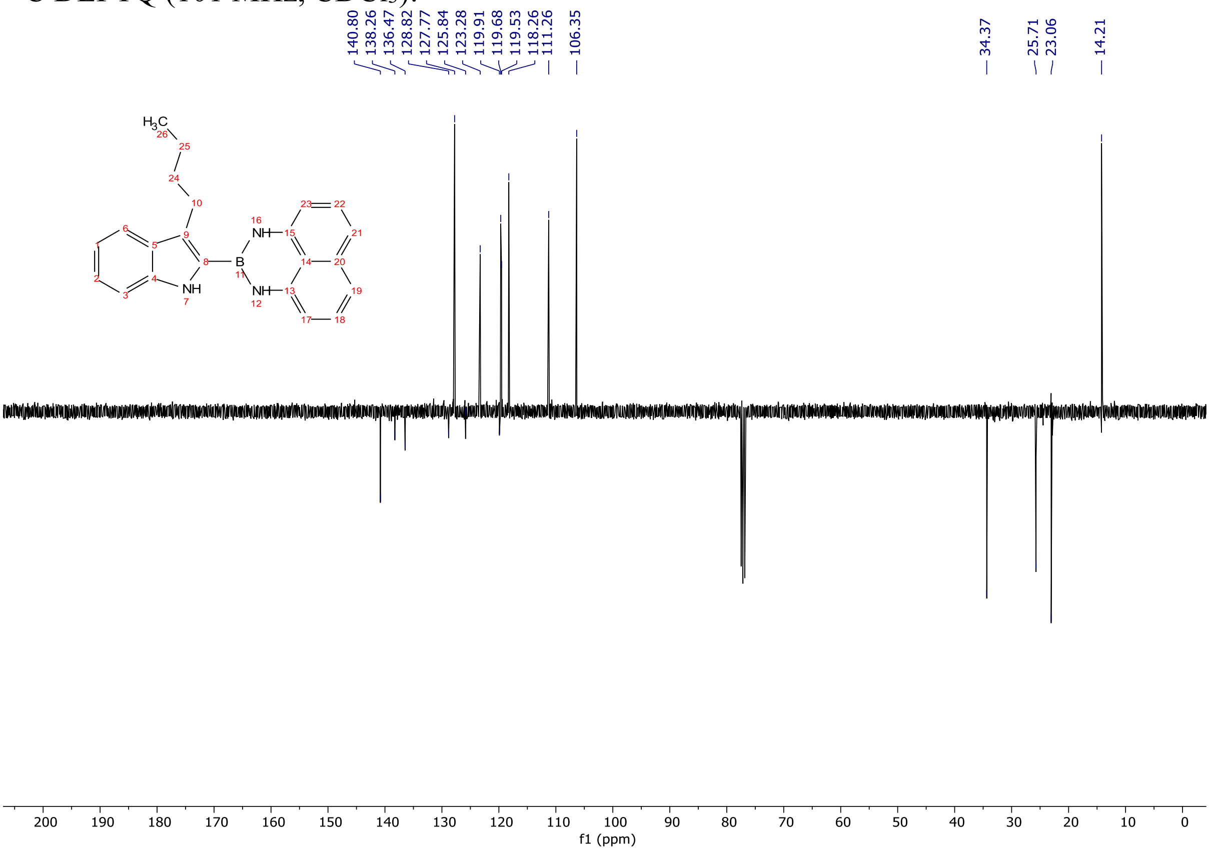

(4b) ^{1}H NMR (400 MHz, $CDCl_3$):

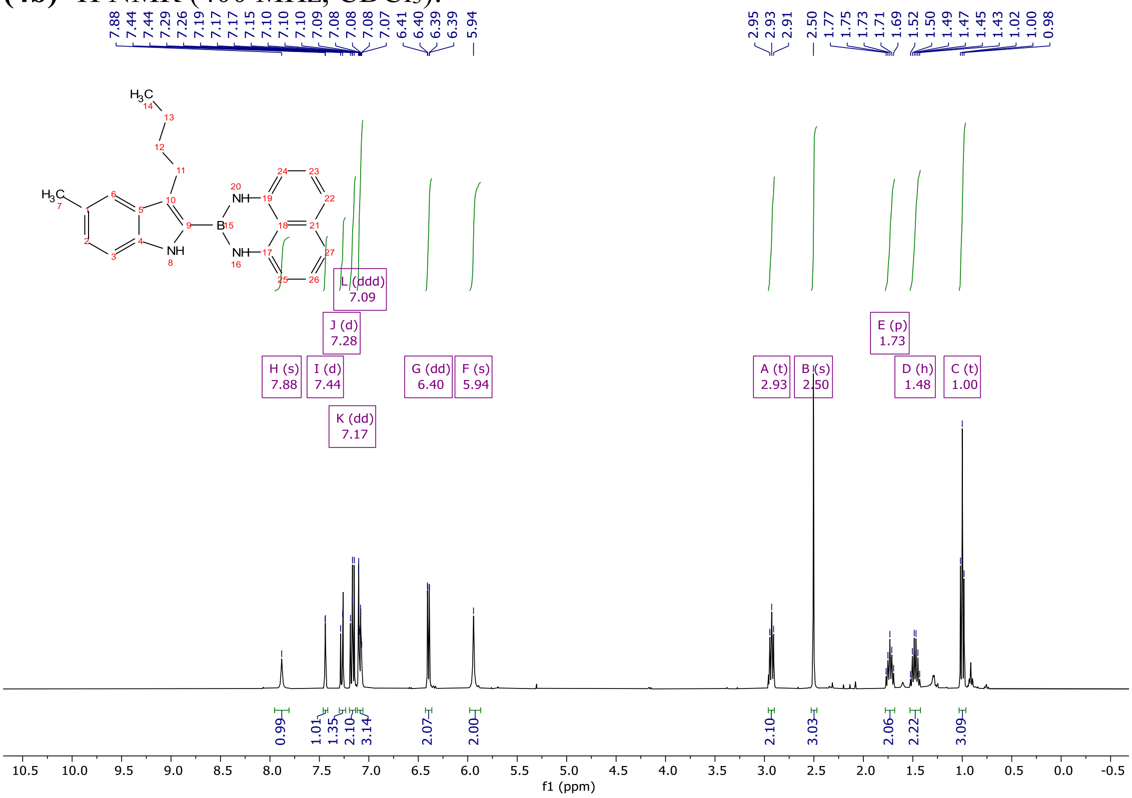

^{13}C DEPTQ (101 MHz, $CDCl_3$):

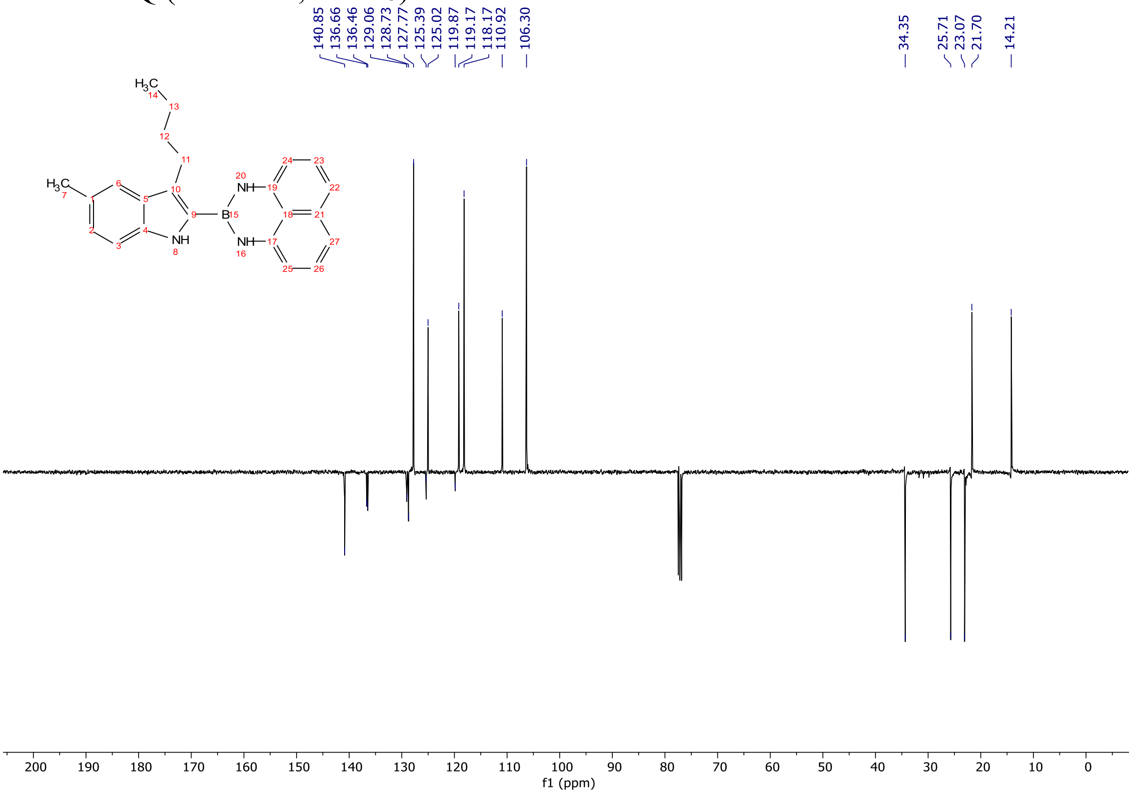

(4c) ^{1}H NMR (400 MHz, $CDCl_3$):

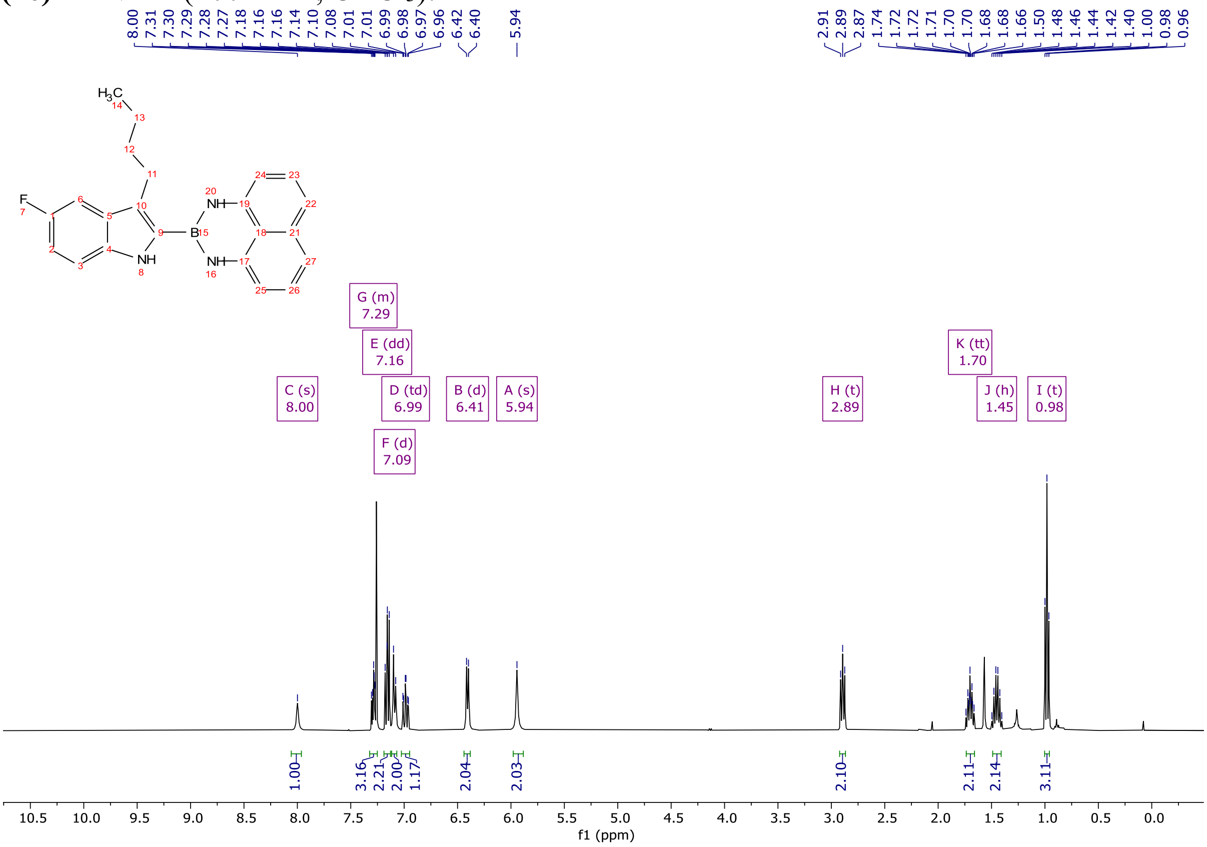

$^{13}C\{^{1}H\}$ NMR (101 MHz, $CDCl_3$):

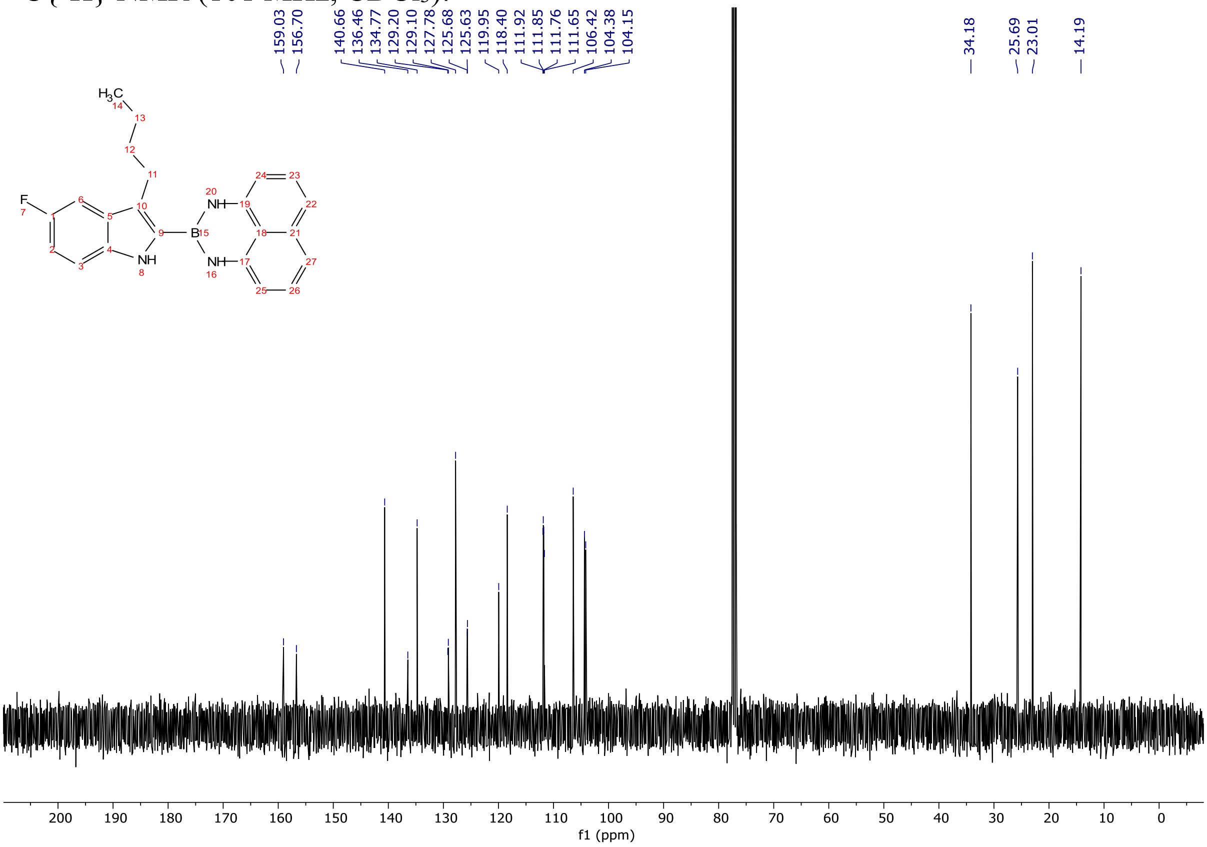

^{19}F NMR (376 MHz, $CDCl_3$):

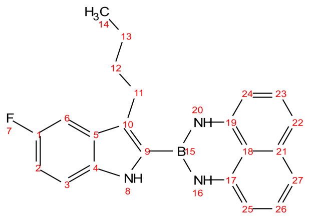

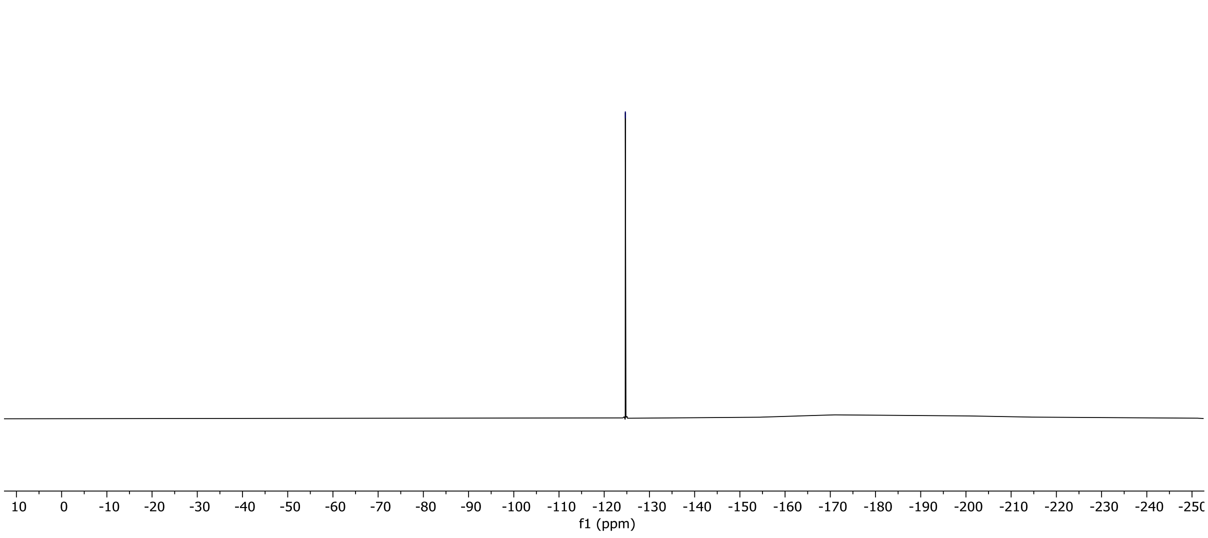

(4d) ^{1}H NMR (400 MHz, $CDCl_3$):

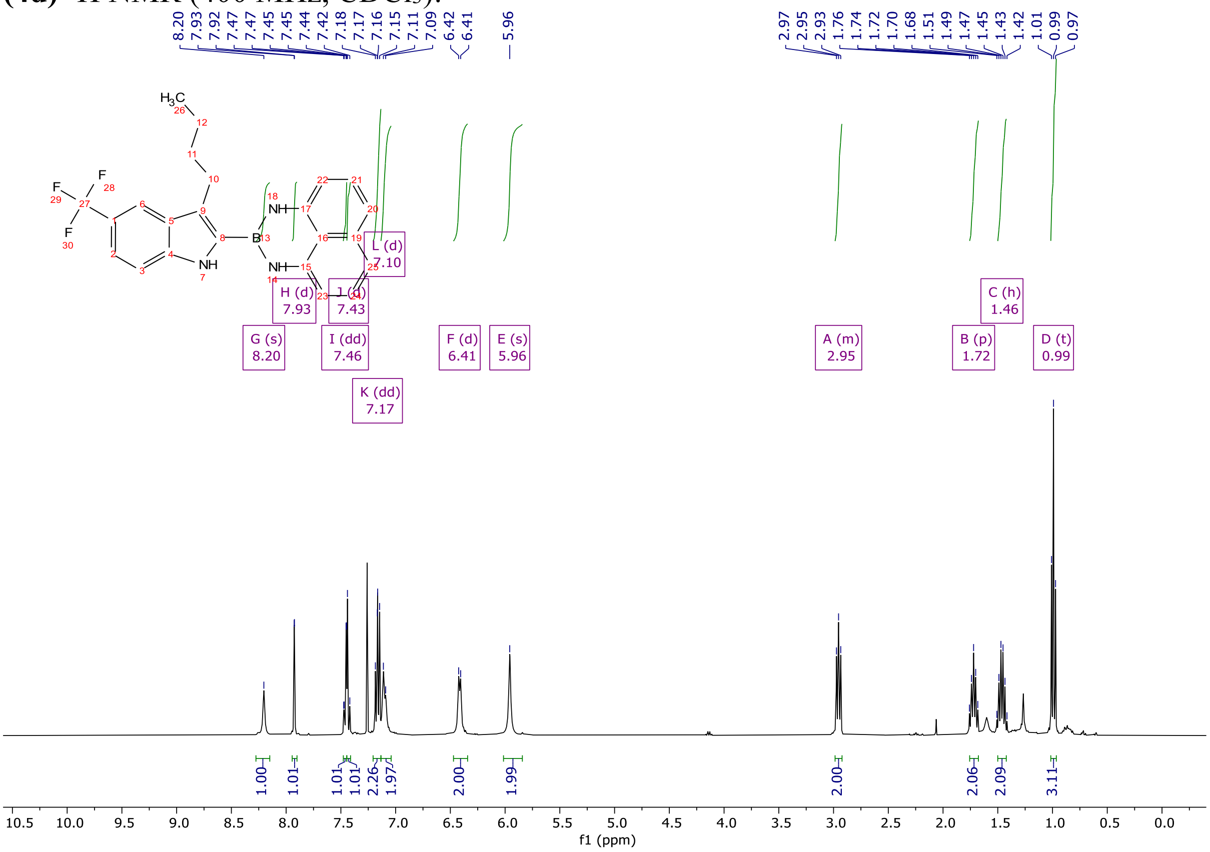

^{13}C{^{1}H} NMR (101 MHz, $CDCl_3$):

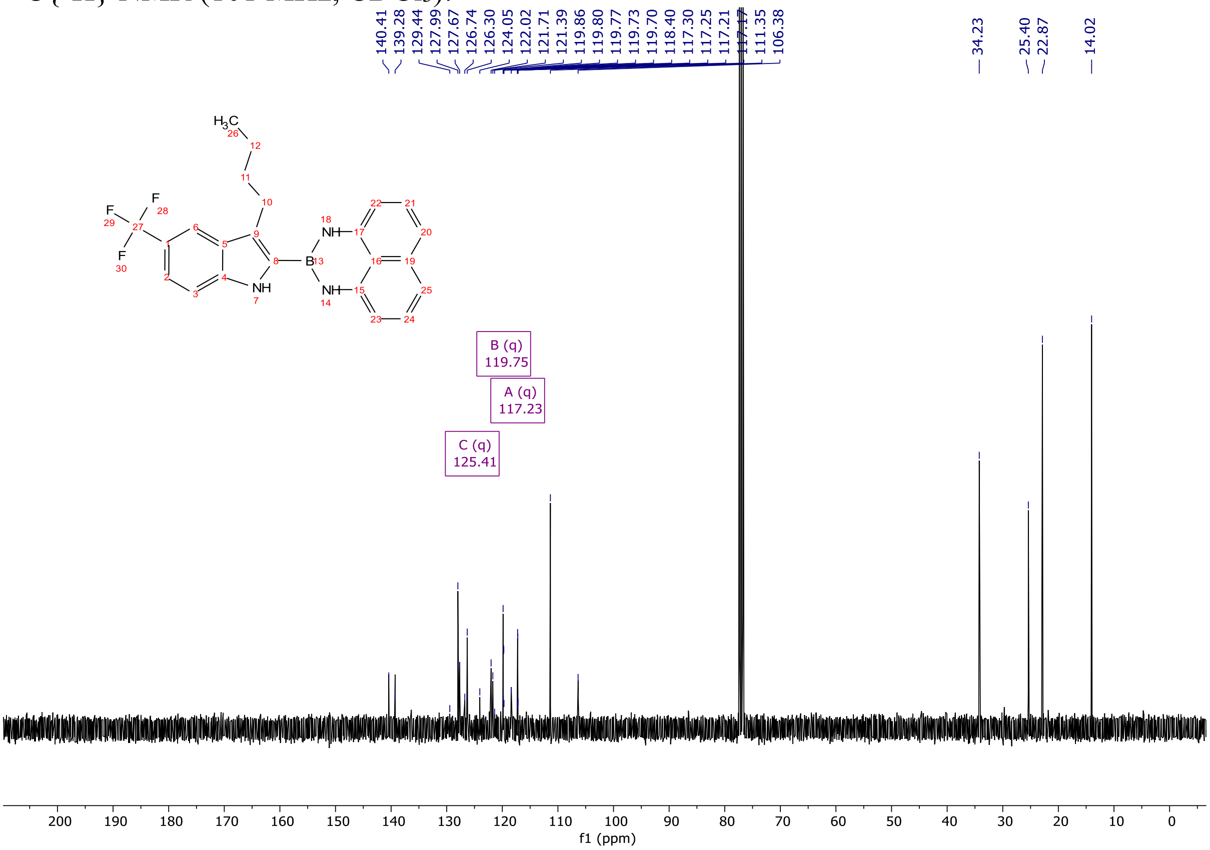

^{19}F NMR (376 MHz, $CDCl_3$):

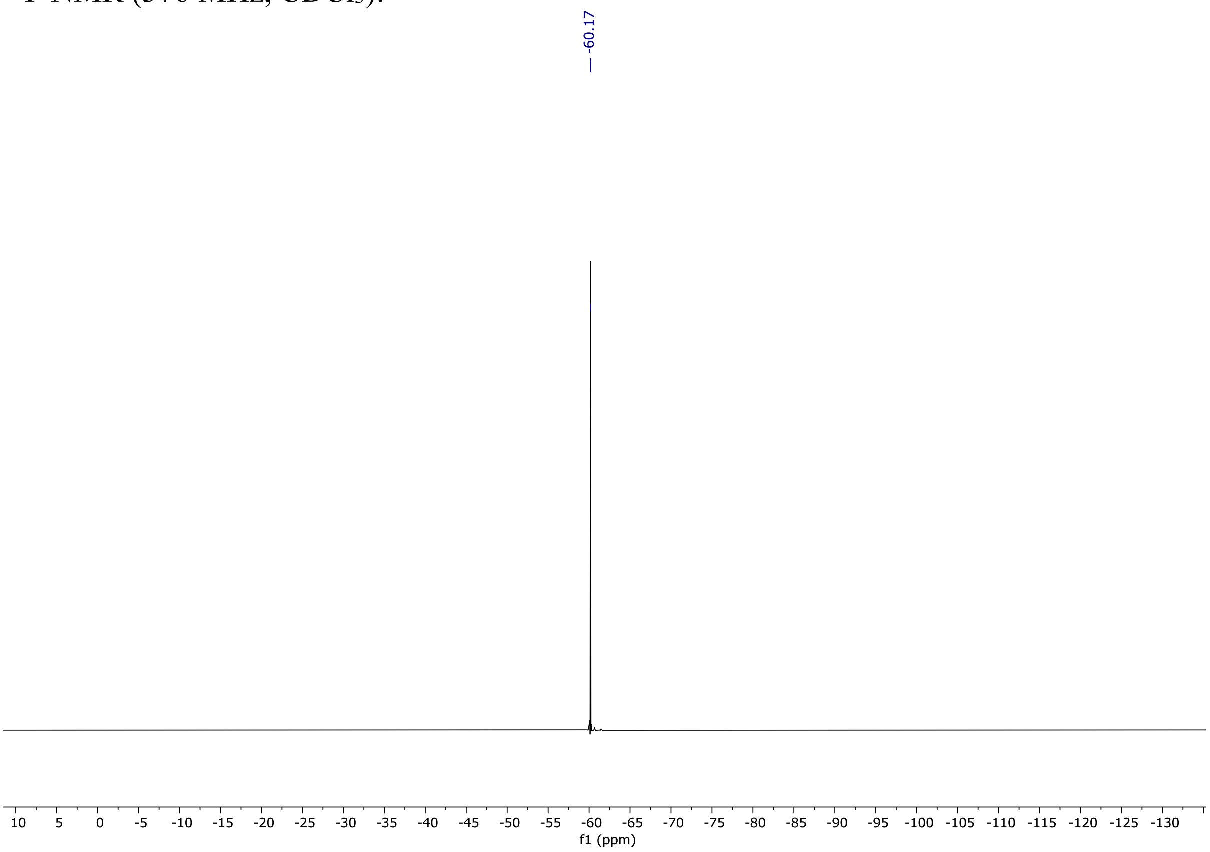

(4e) ^{1}H NMR (700 MHz, $CDCl_3$):

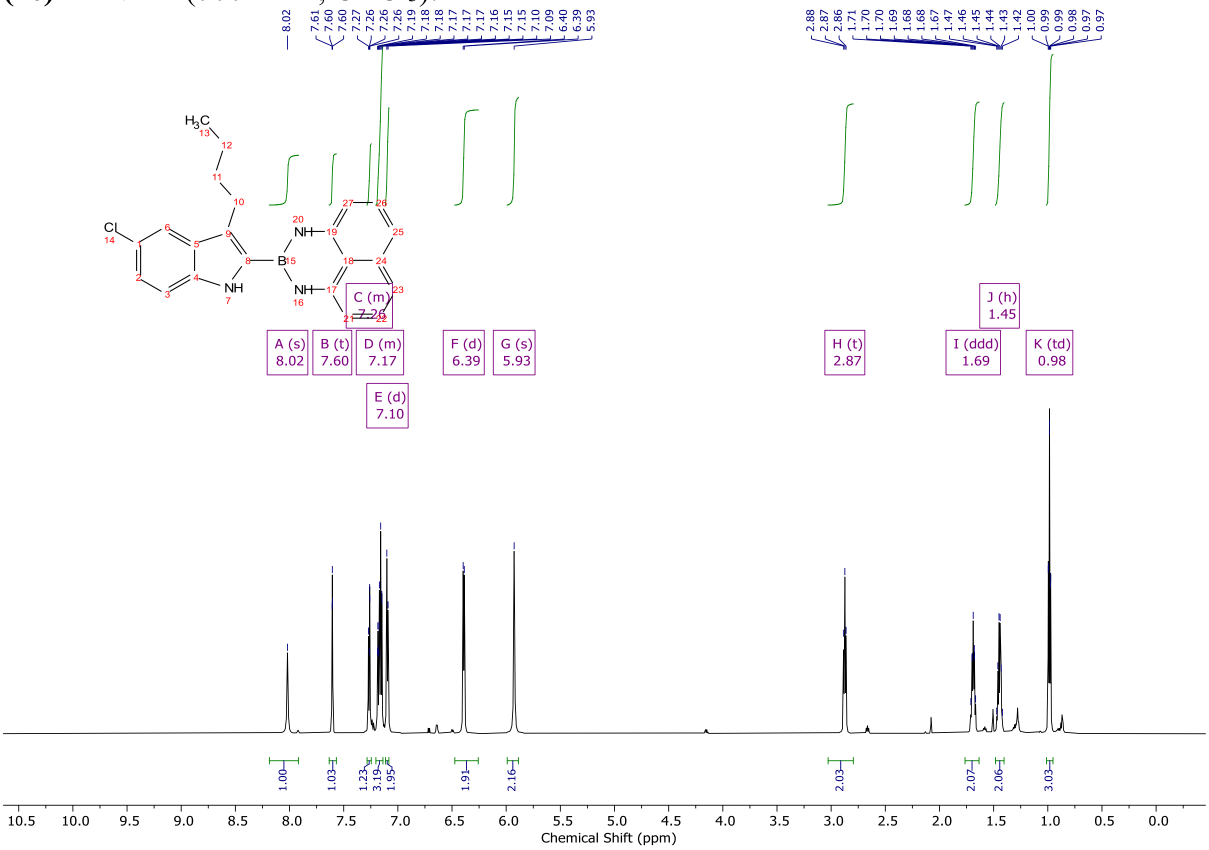

$^{13}C\{^{1}H\}$ NMR (176 MHz, $CDCl_3$):

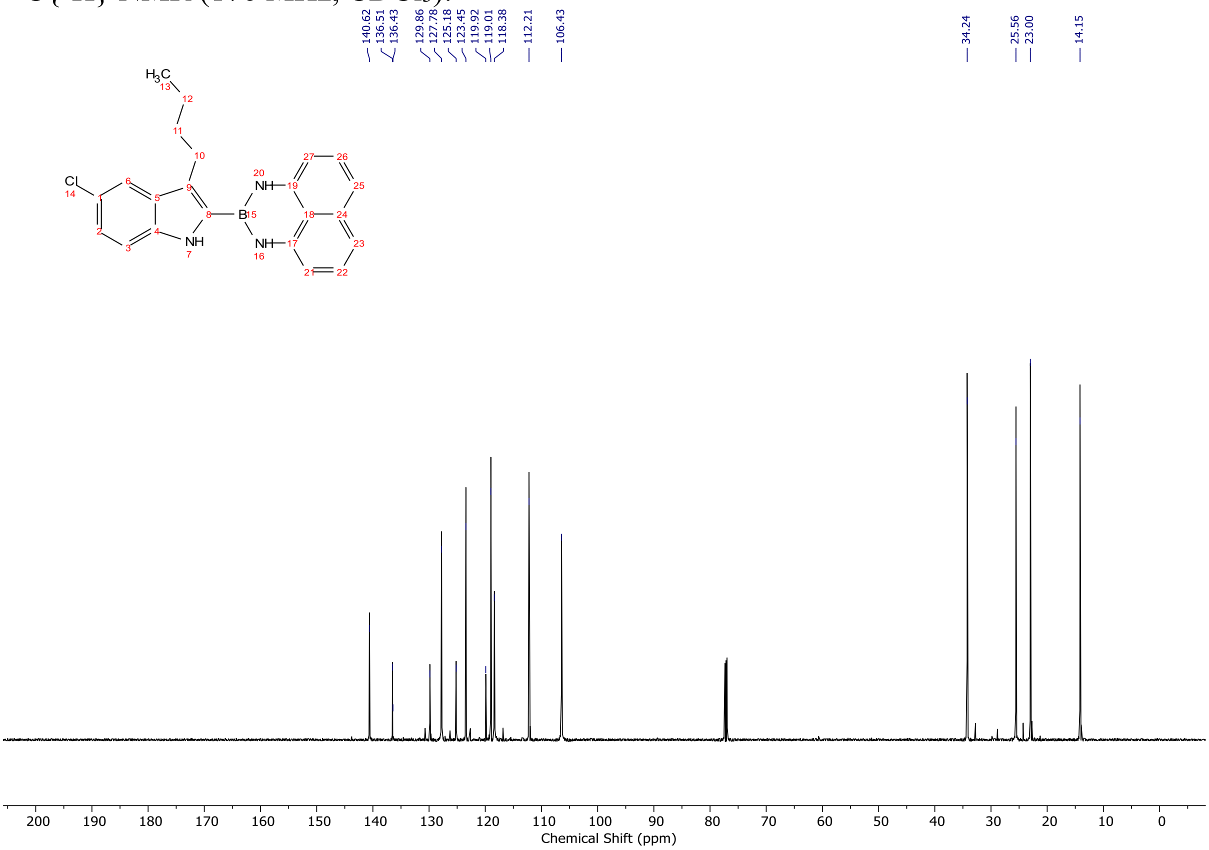

(4f) ^{1}H NMR (500 MHz, $CDCl_3$):

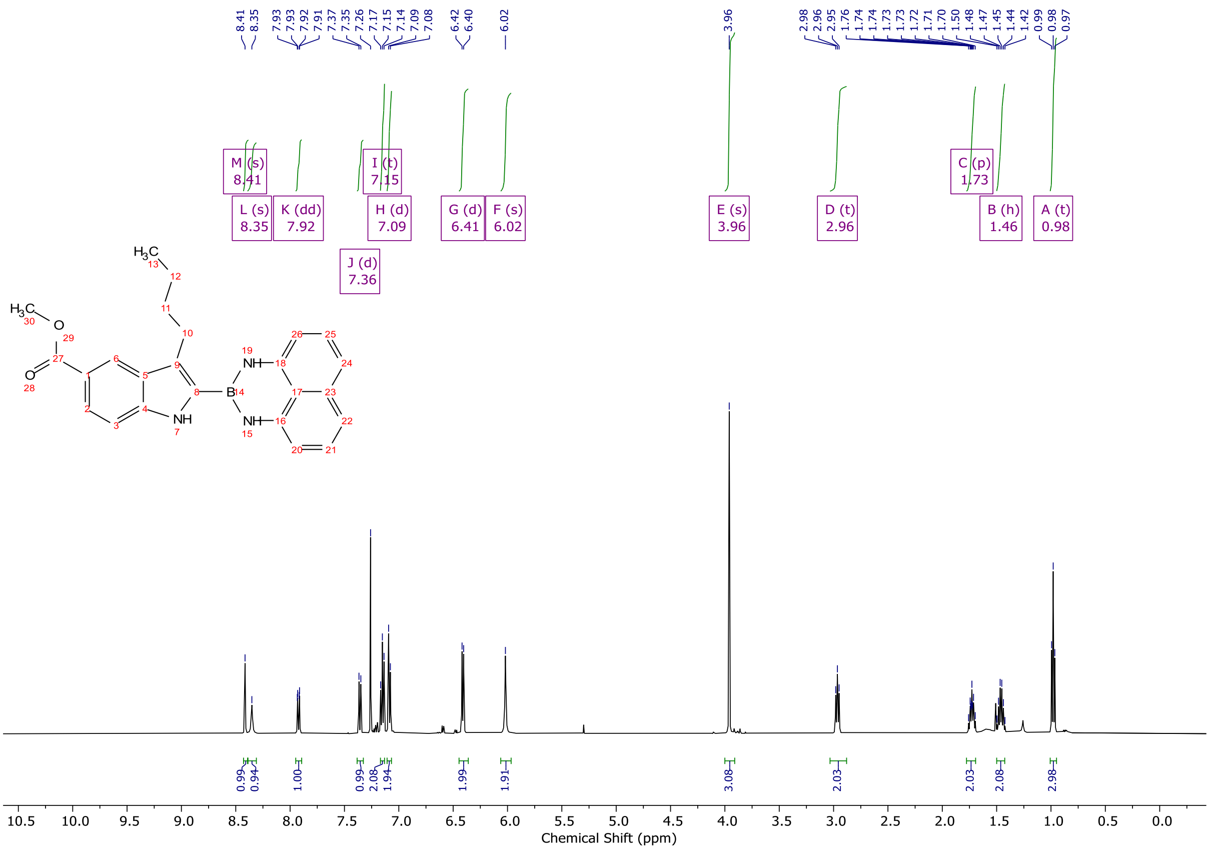

^{13}C DEPTQ (126 MHz, $CDCl_3$):

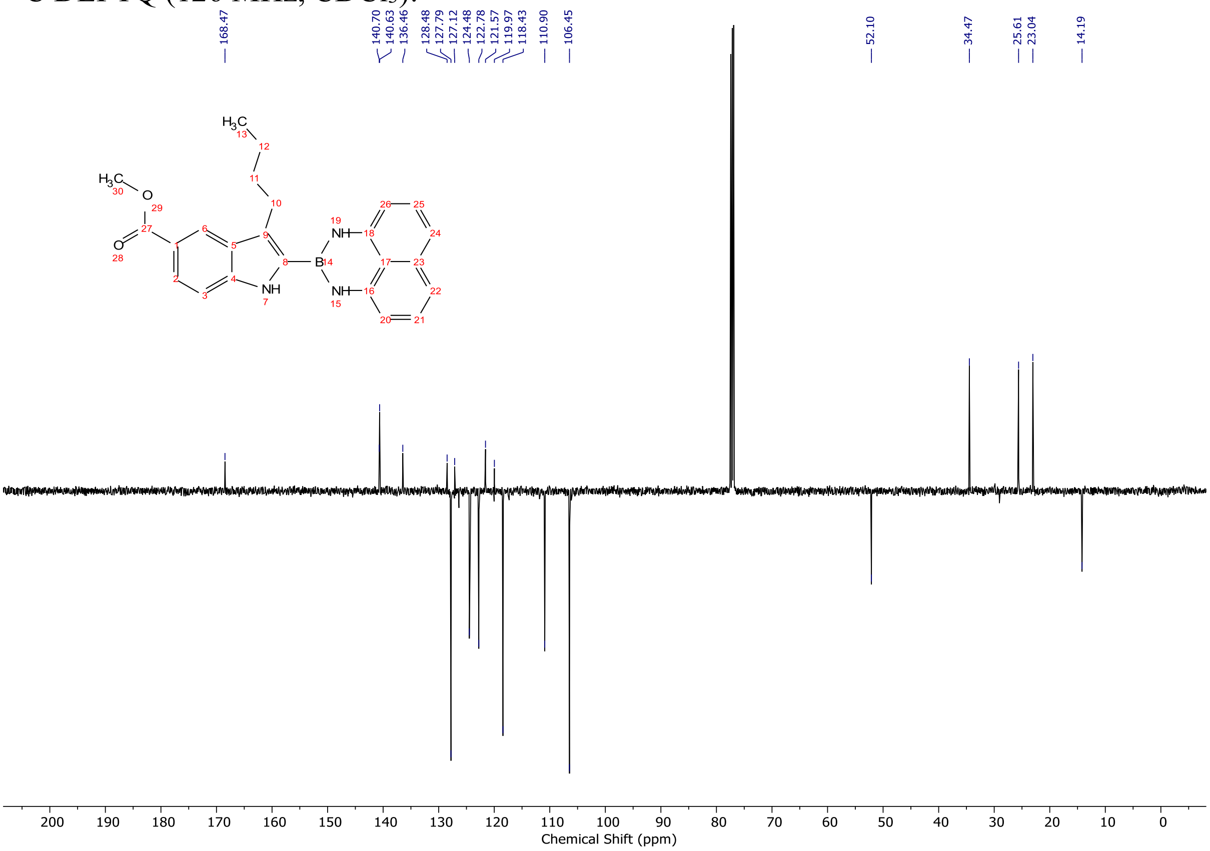

(4g) ^{1}H NMR (700 MHz, $CDCl_3$):

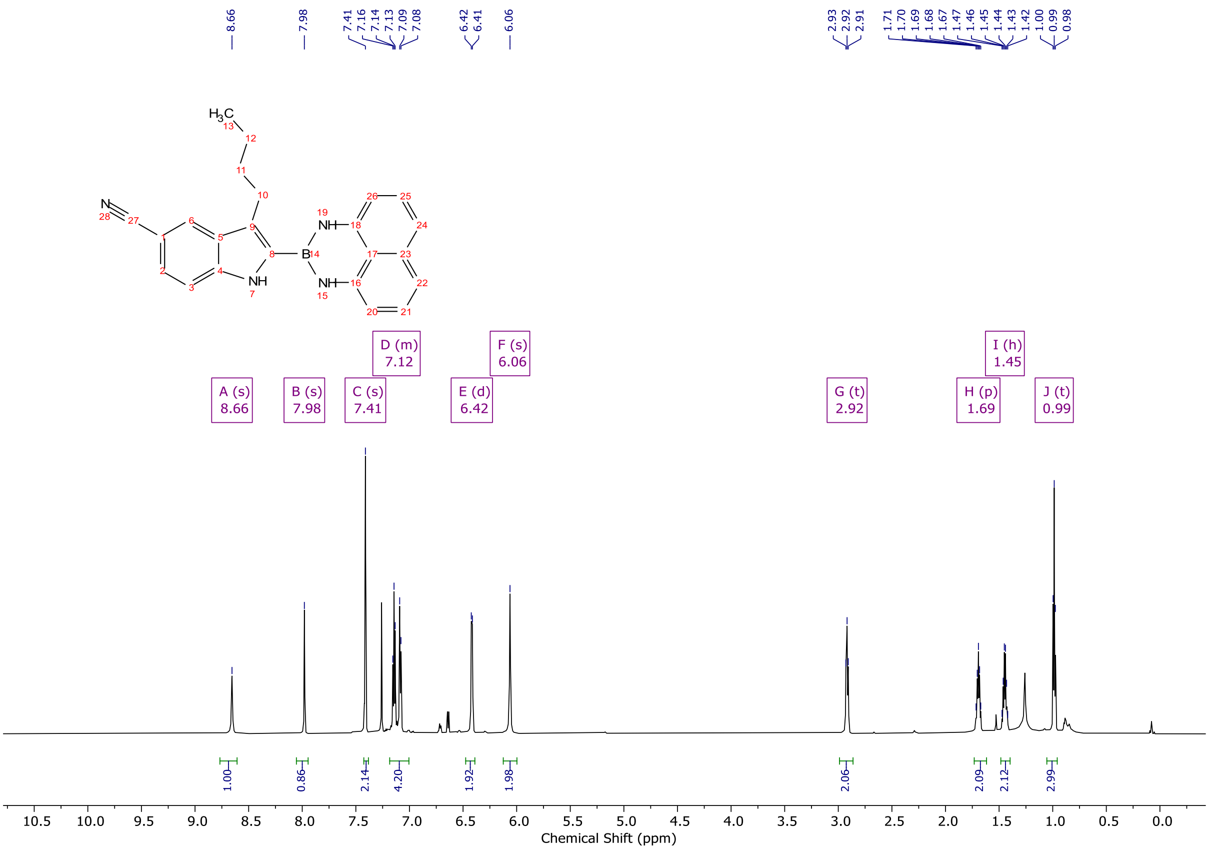

^{13}C DEPTQ (176 MHz, $CDCl_3$):

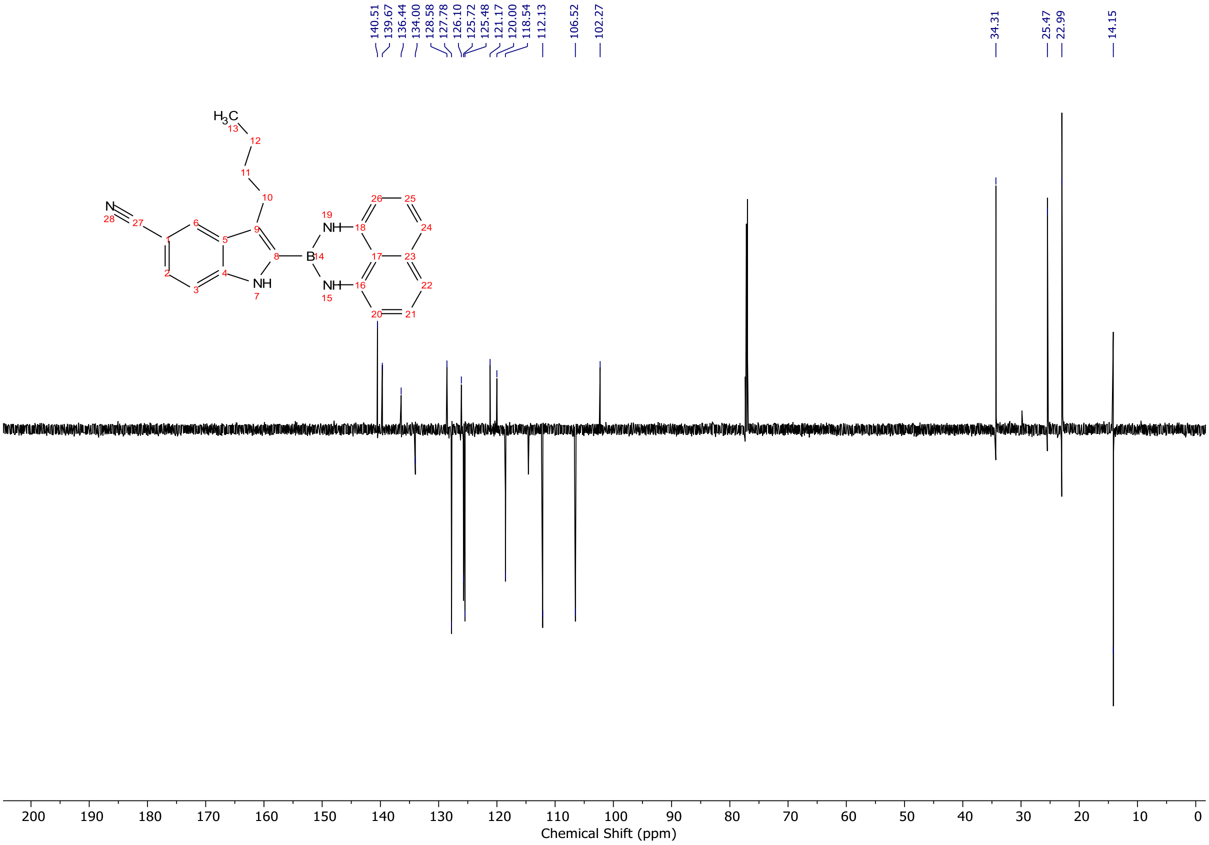

(4h) ^{1}H NMR (400 MHz, $CDCl_3$):

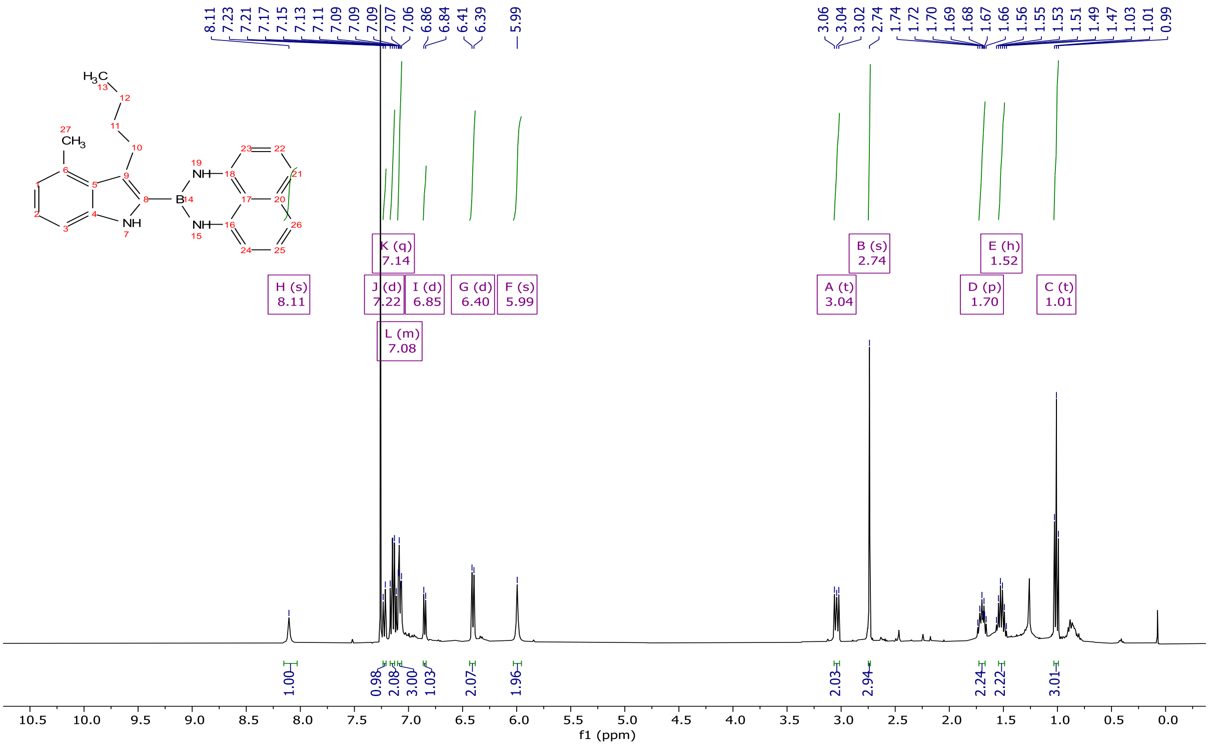

1313C{^{1}H} NMR (101 MHz, $CDCl_3$):

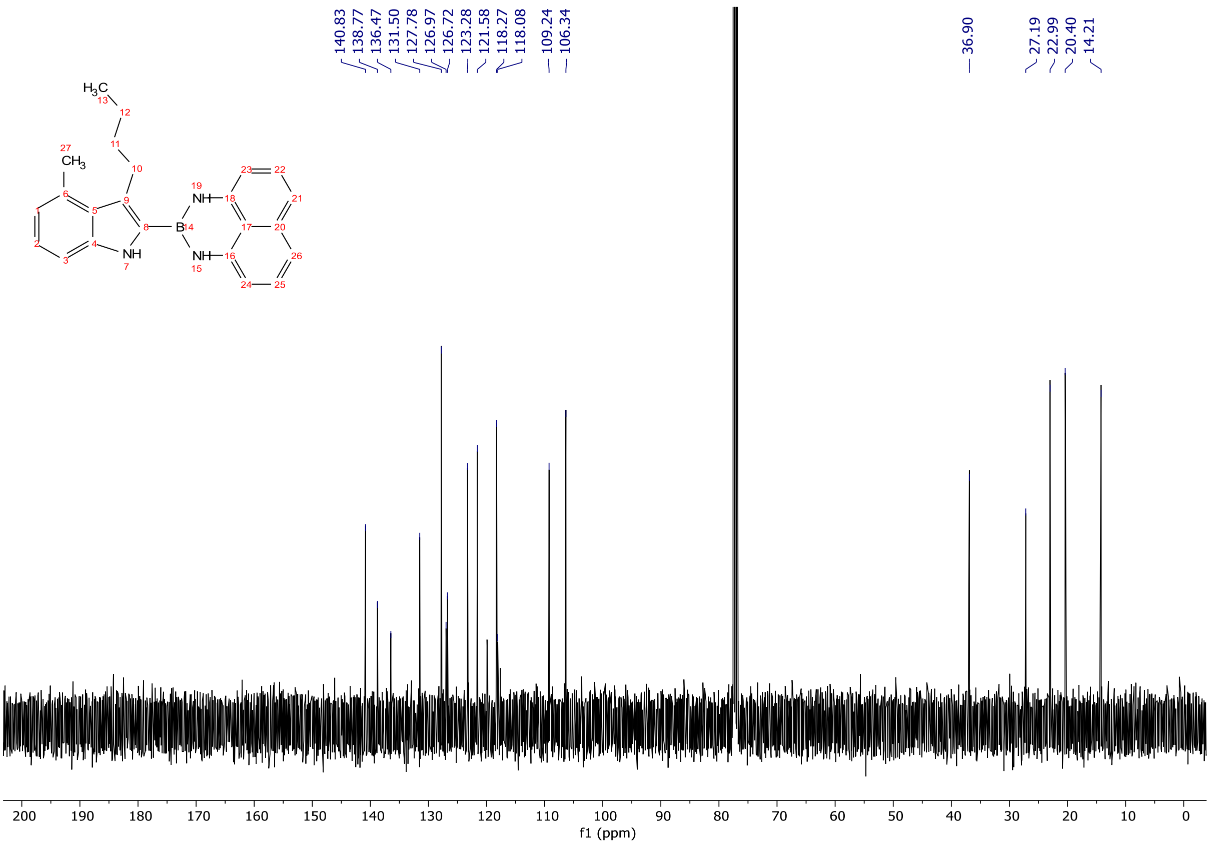

(4i) ^{1}H NMR (400 MHz, $CDCl_3$):

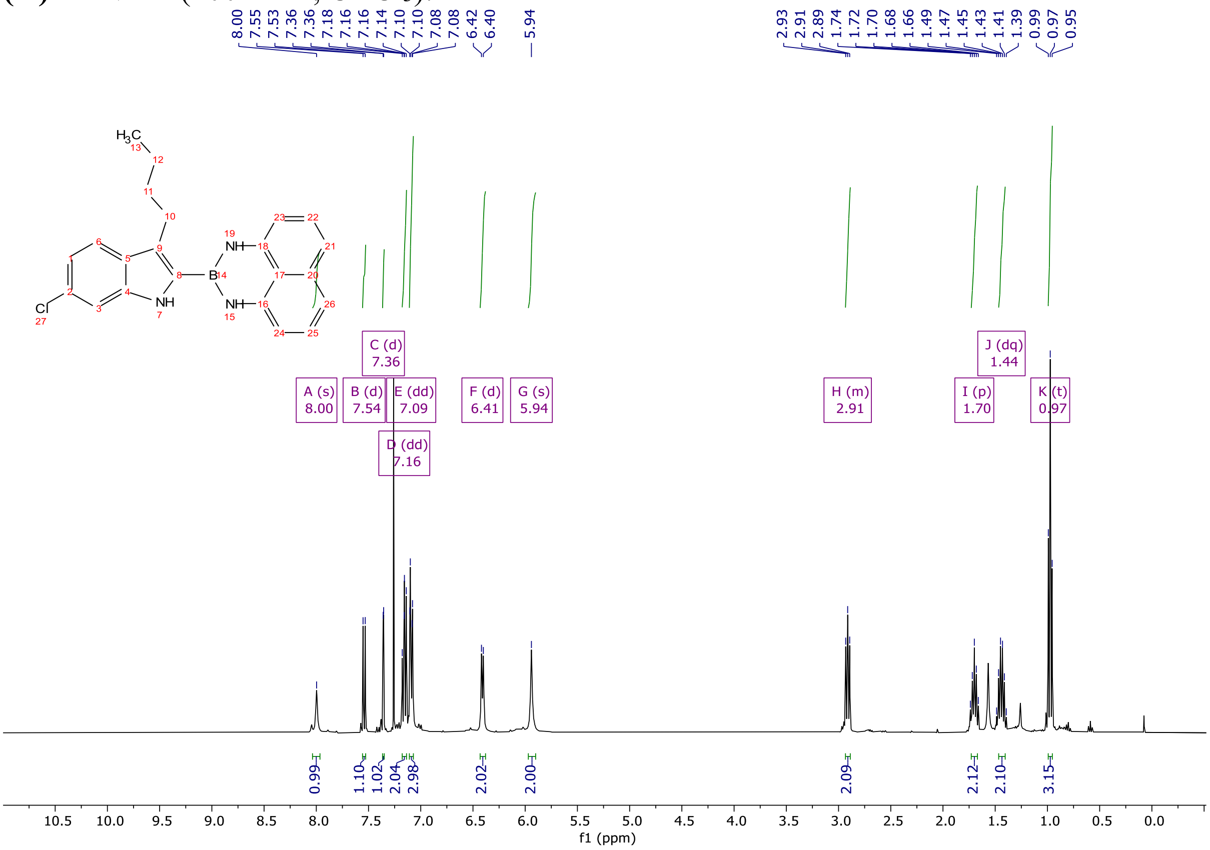

^{13}C DEPTQ (101 MHz, $CDCl_3$):

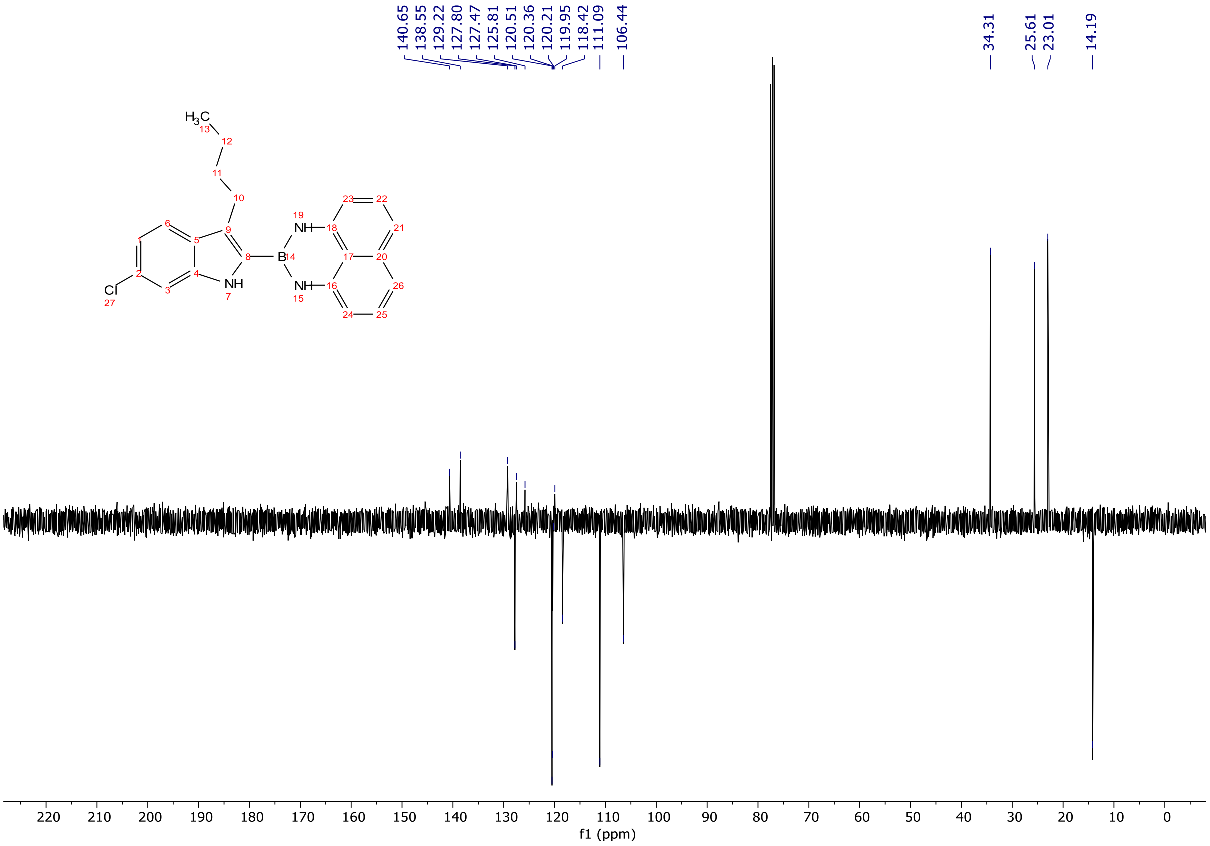

(4j) ^{1}H NMR (500 MHz, $CDCl_3$):

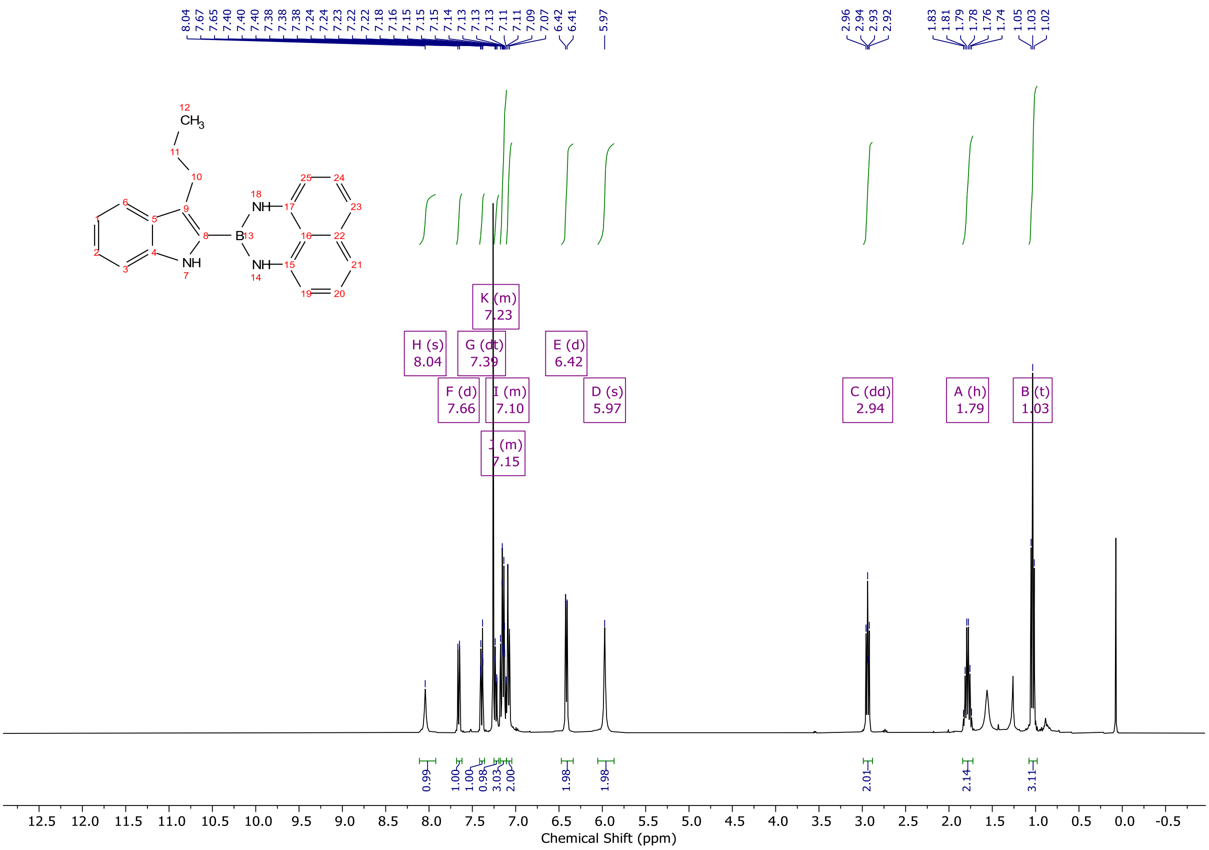

$^{13}C\{^{1}H\}$ NMR (126 MHz, $CDCl_3$):

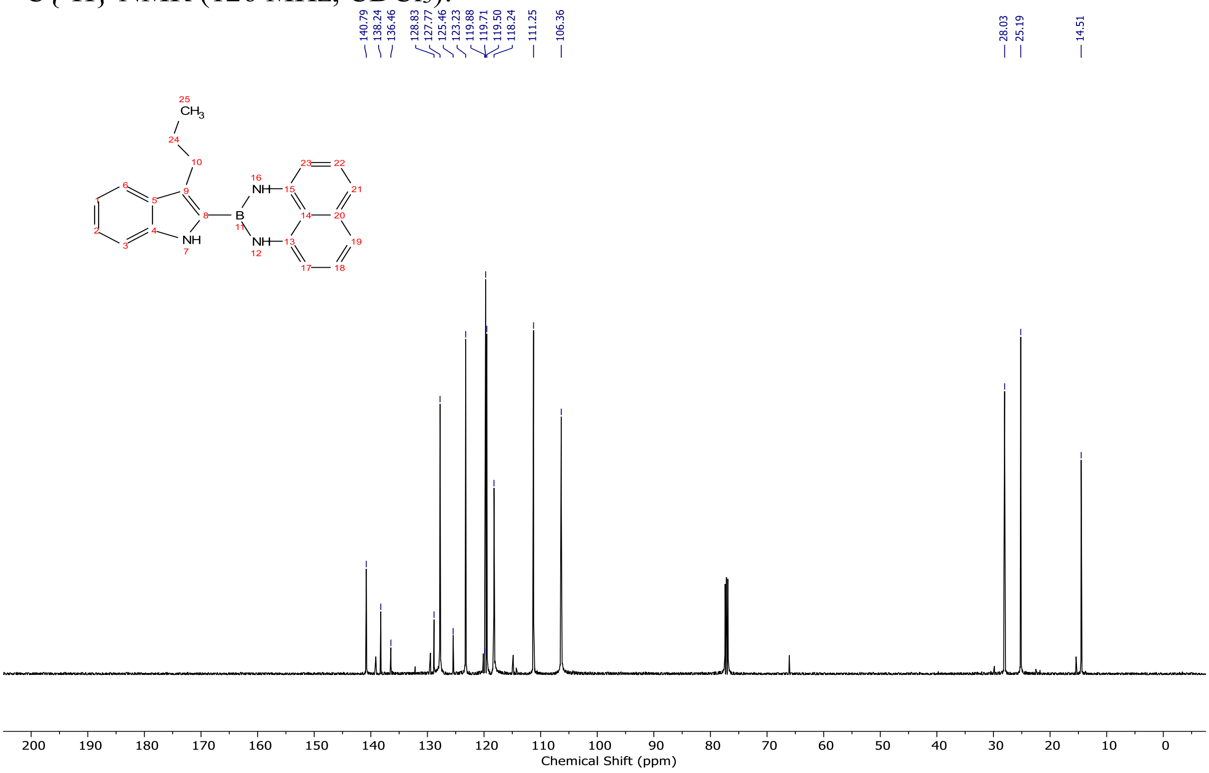

(4k) ^{1}H NMR (400 MHz, $CDCl_3$):

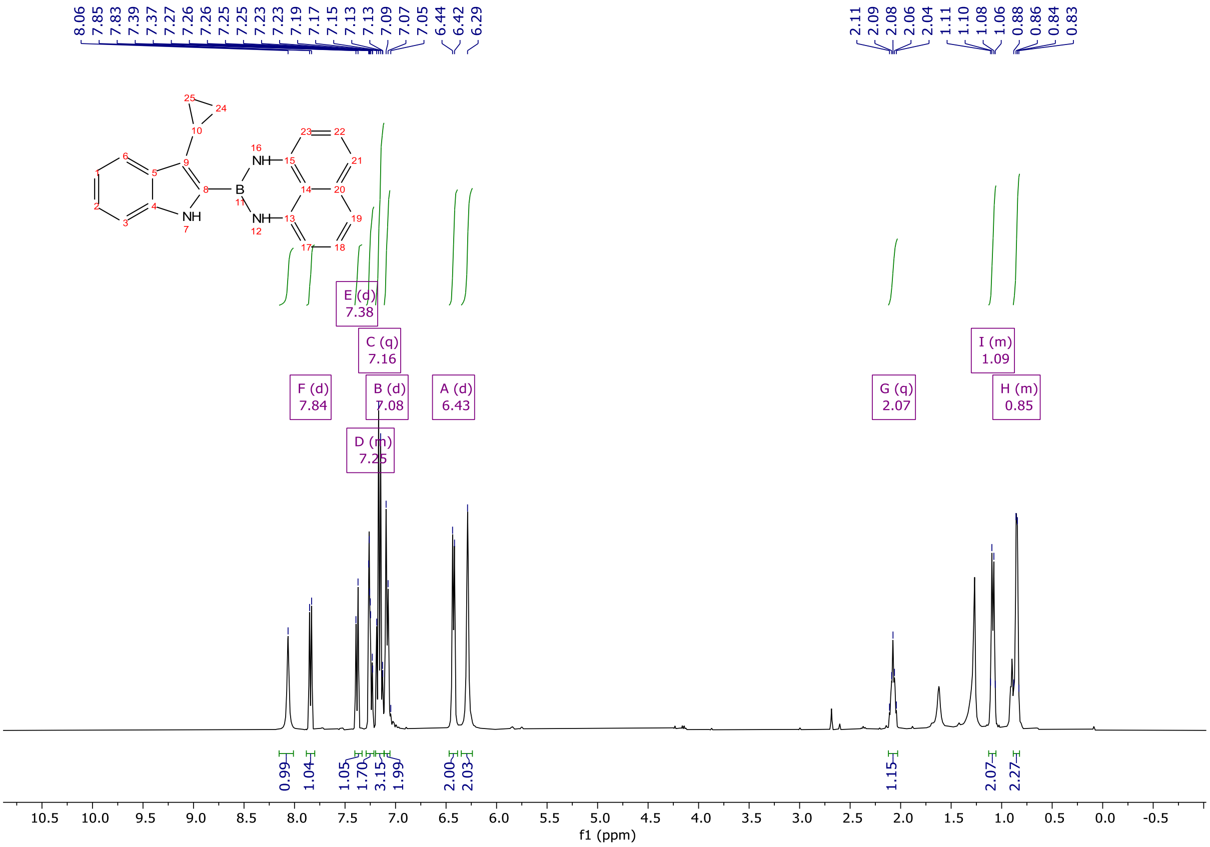

$^{13}C\{^1H\}$ NMR (101 MHz, $CDCl_3$):

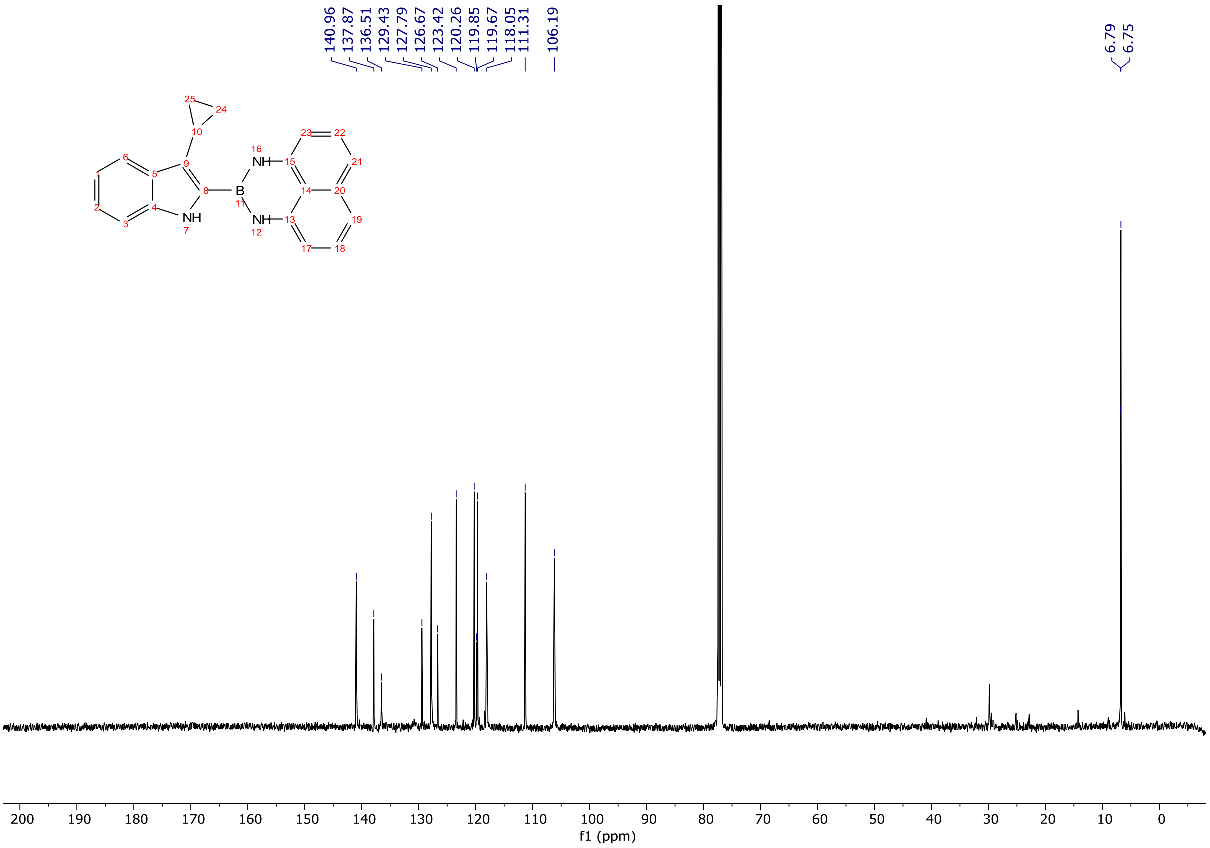

(4l) ^{1}H NMR (400 MHz, $CDCl_3$):

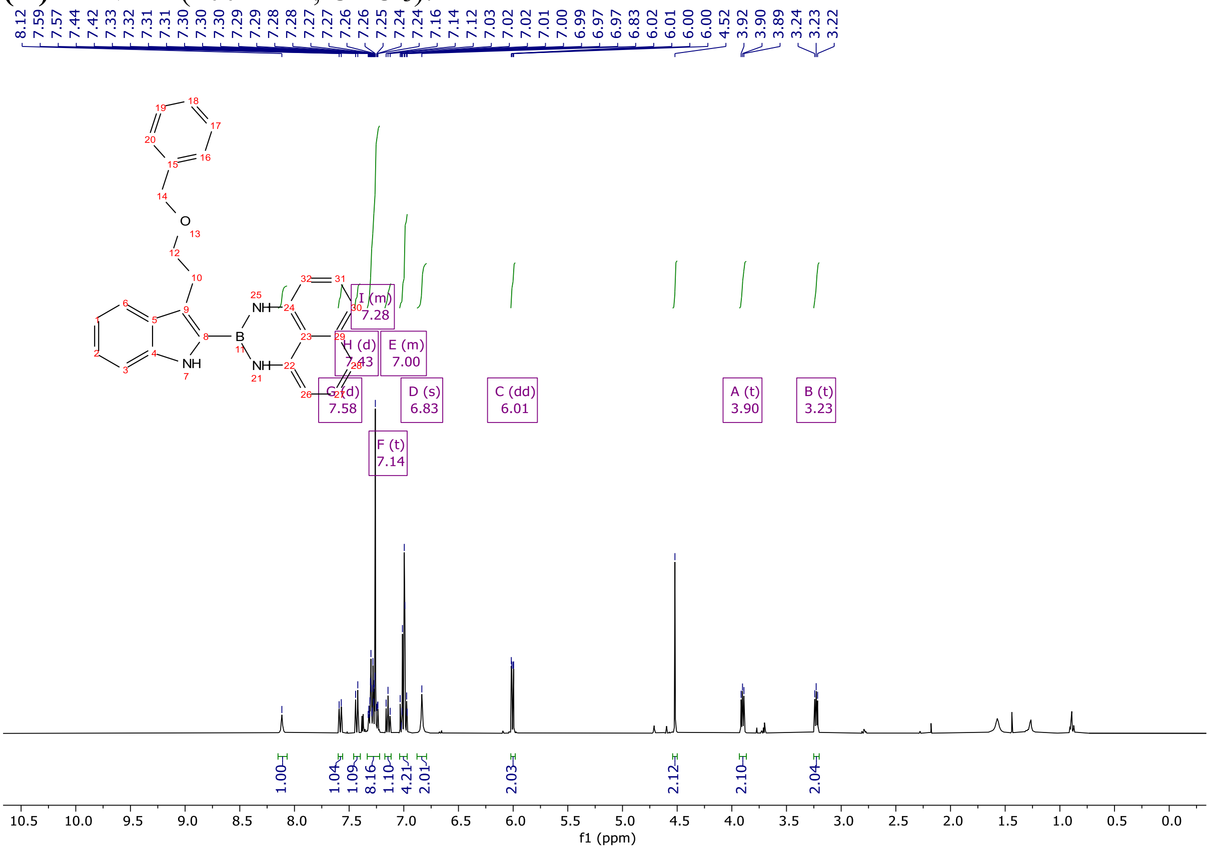

$^{13}C\{^{1}H\}$ NMR (101 MHz, $CDCl_3$):

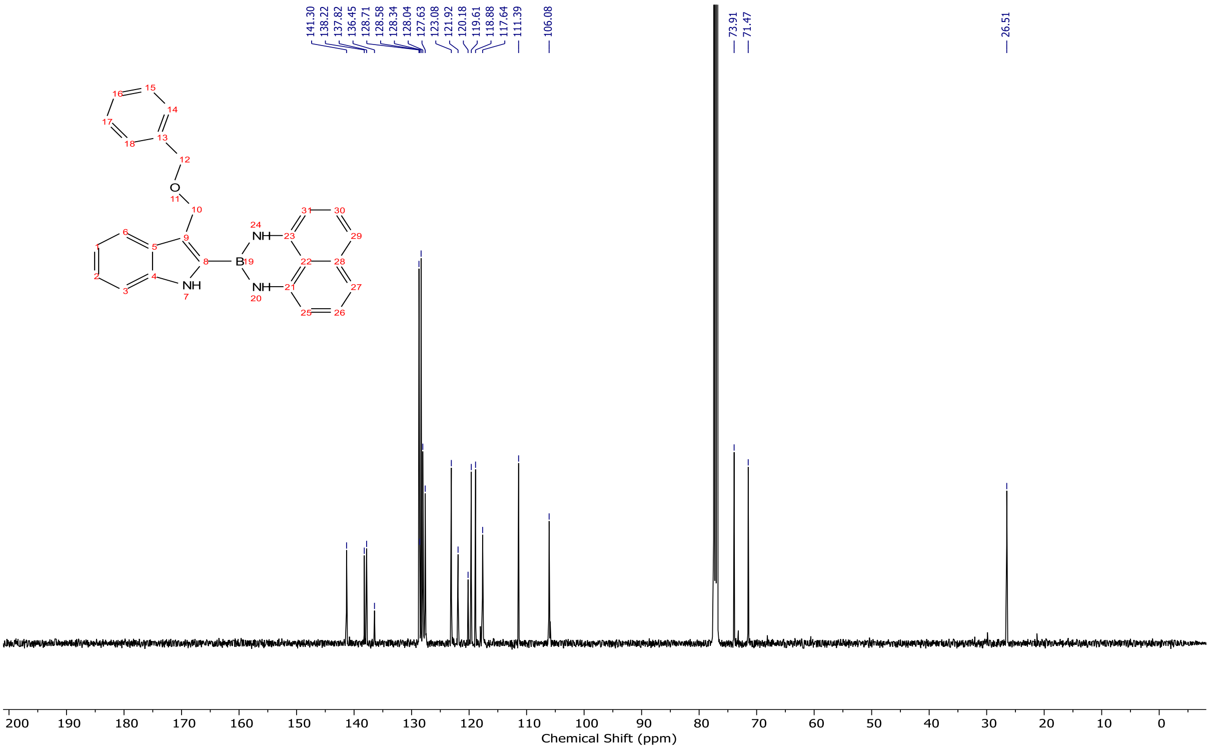

(4m) ^{1}H NMR (400 MHz, CDCl$_3$):

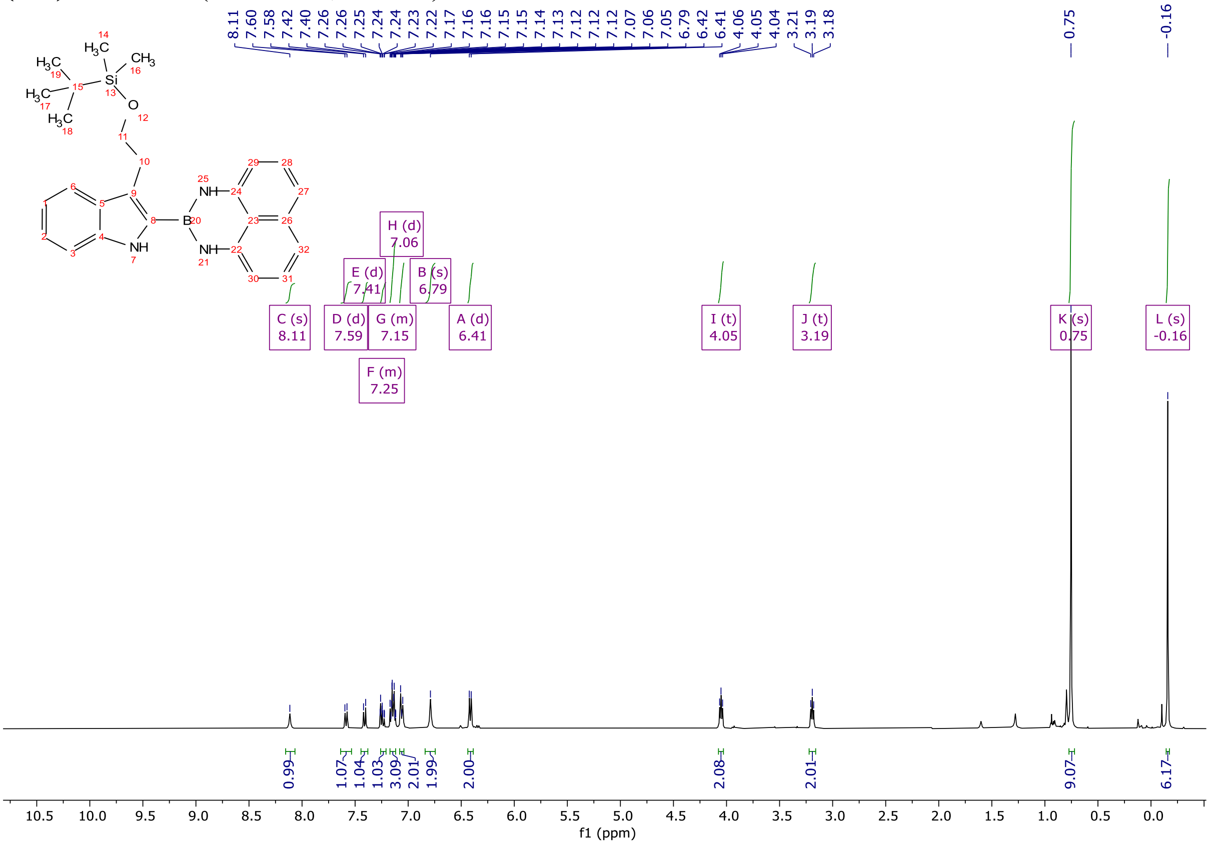

^{13}C DEPTQ (101 MHz, CDCl$_3$):

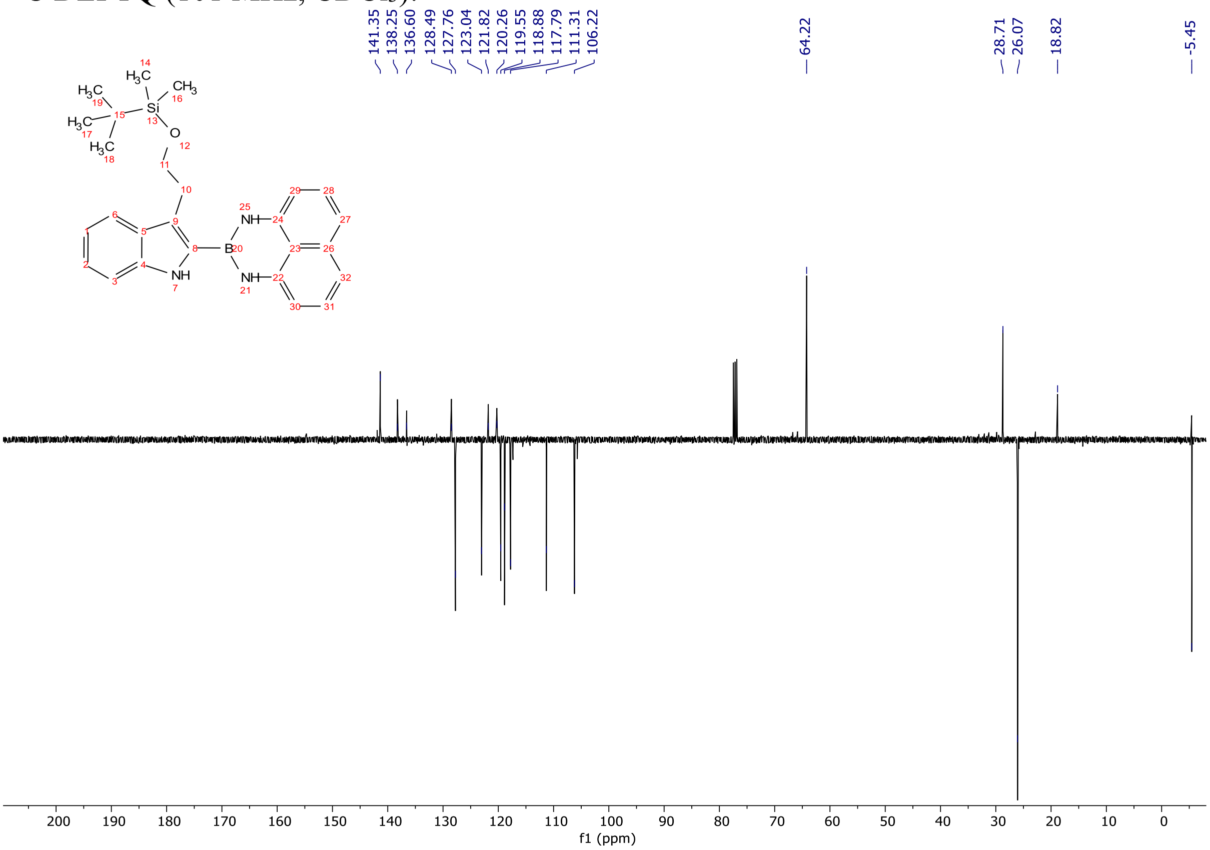

(4n) ^{1}H NMR (400 MHz, $CDCl_3$):

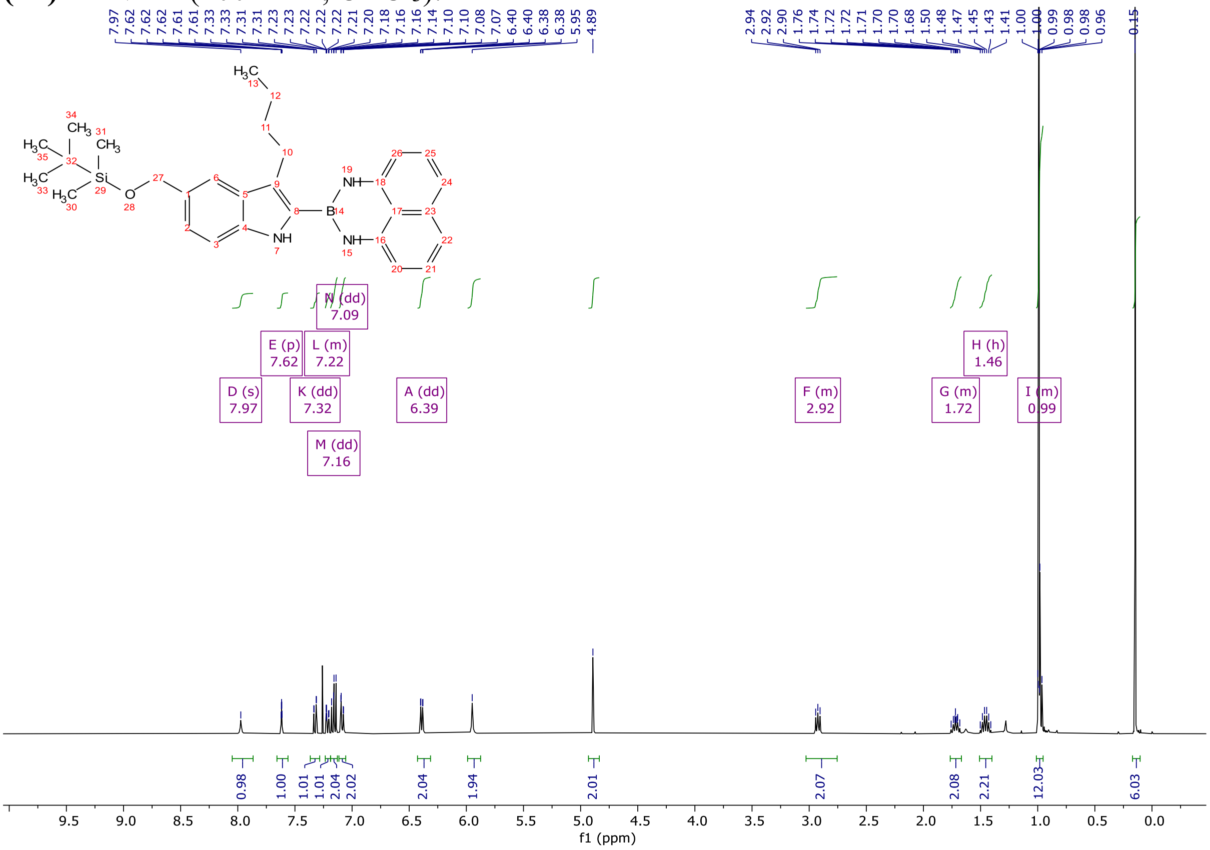

^{13}C DEPTQ (101 MHz, $CDCl_3$):

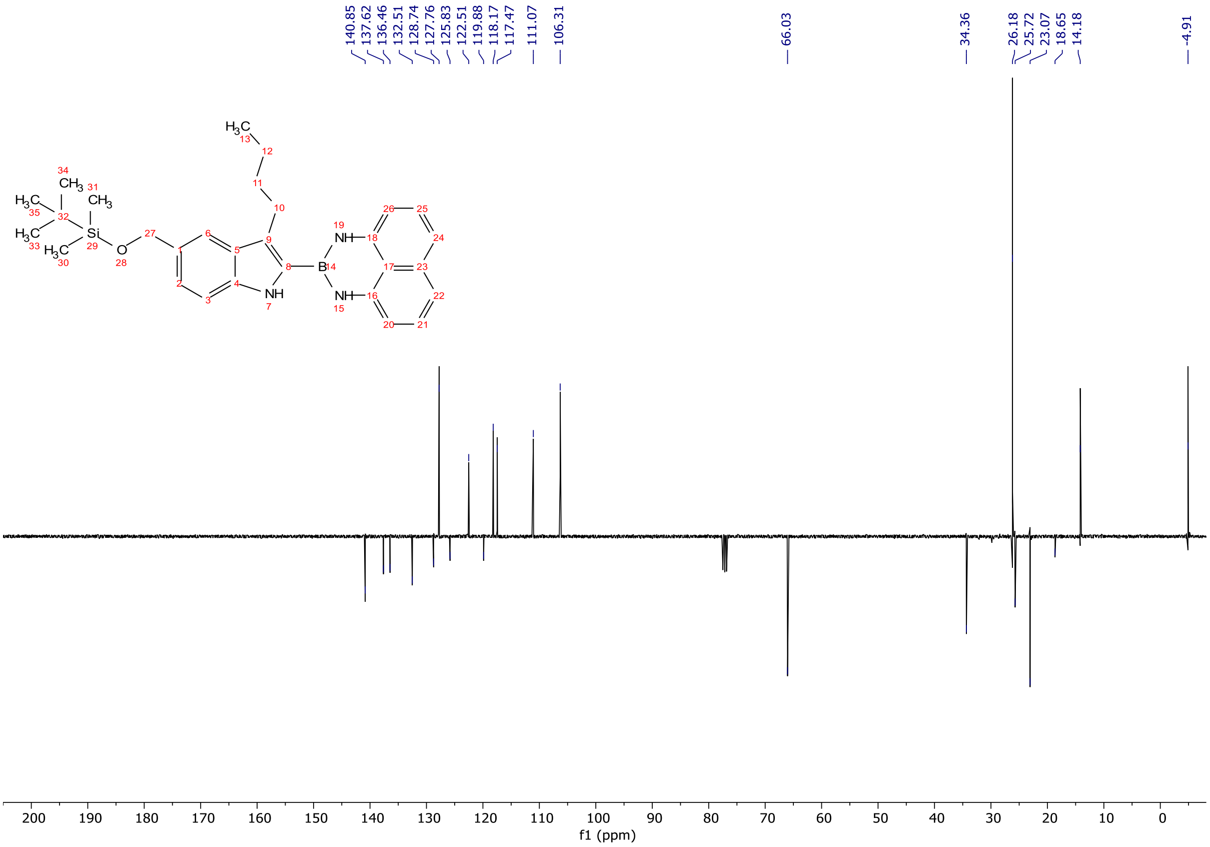

(4o) ^{1}H NMR (500 MHz, Acetone−d_6):

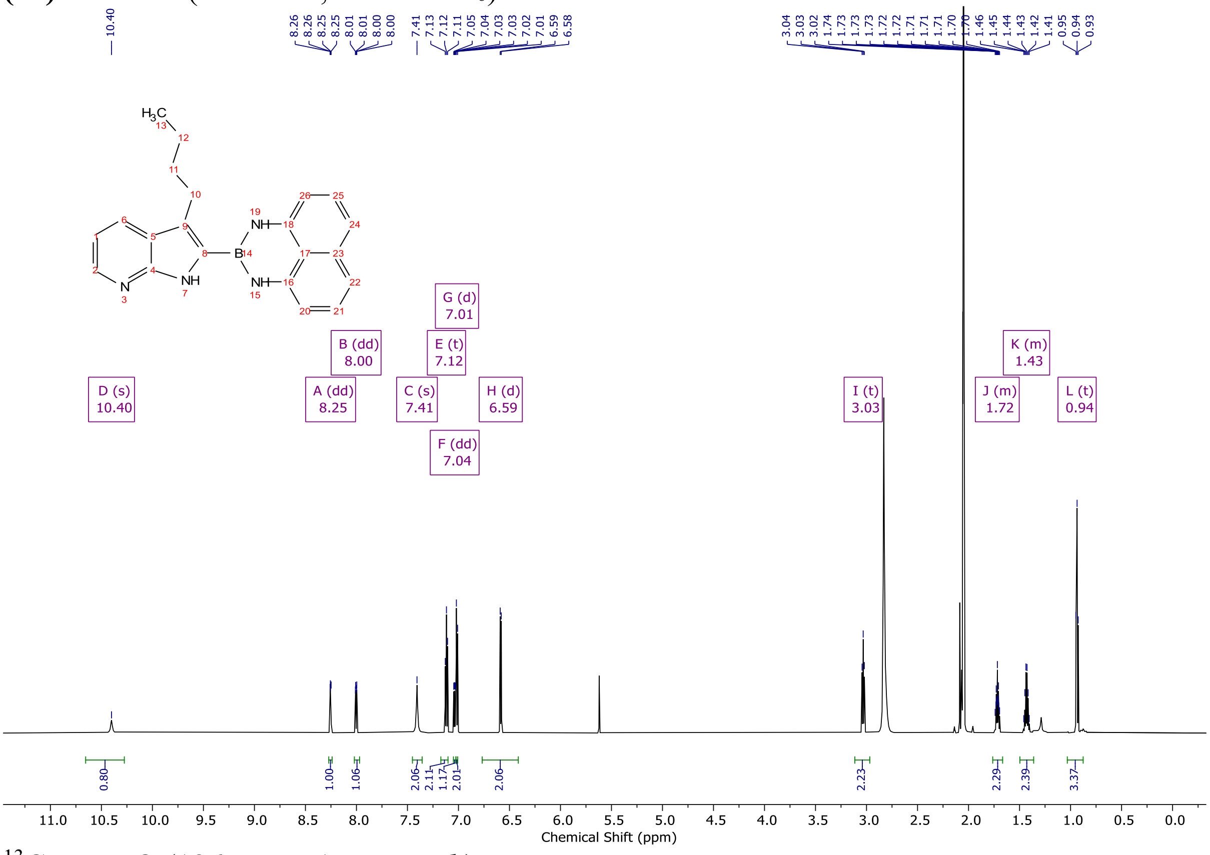

^{13}C DEPTQ (126 MHz, Acetone−d_6):

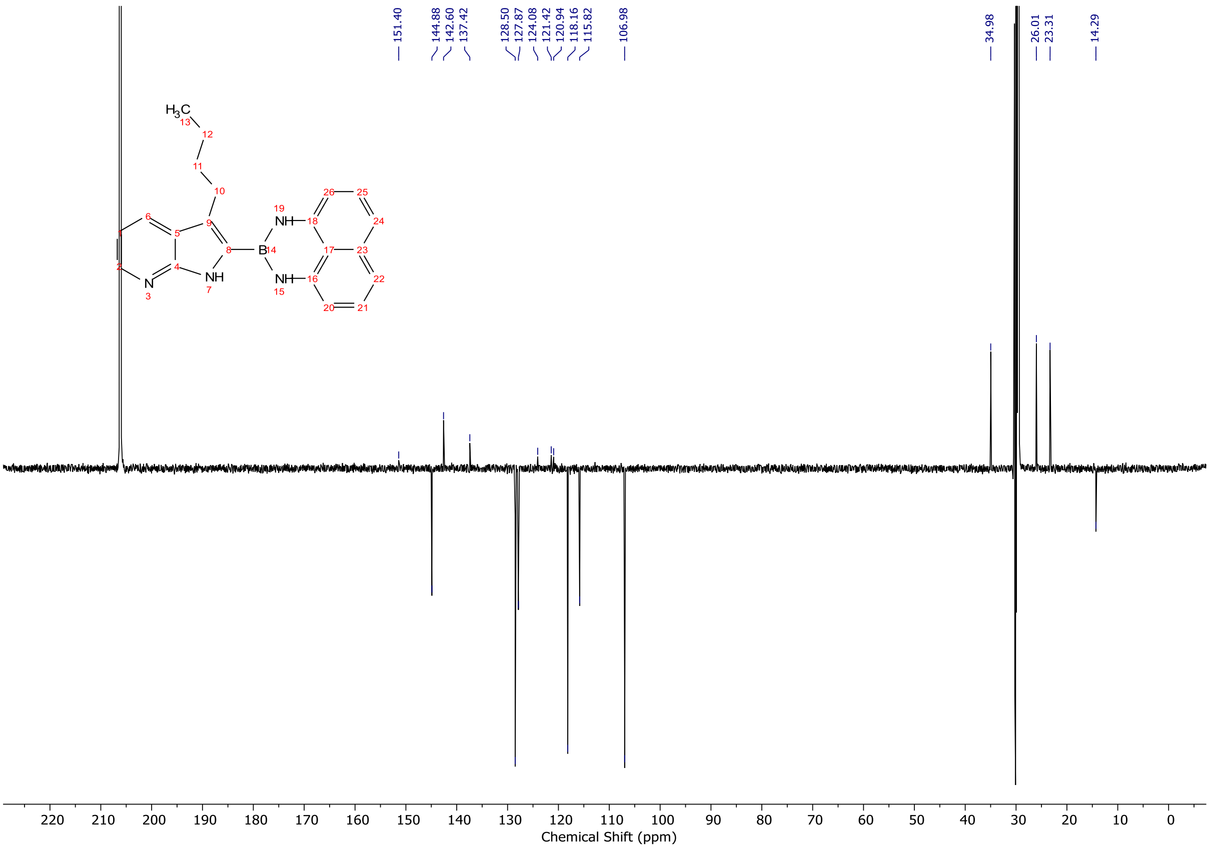

(5a) ^{1}H NMR (400 MHz, $CDCl_3$):

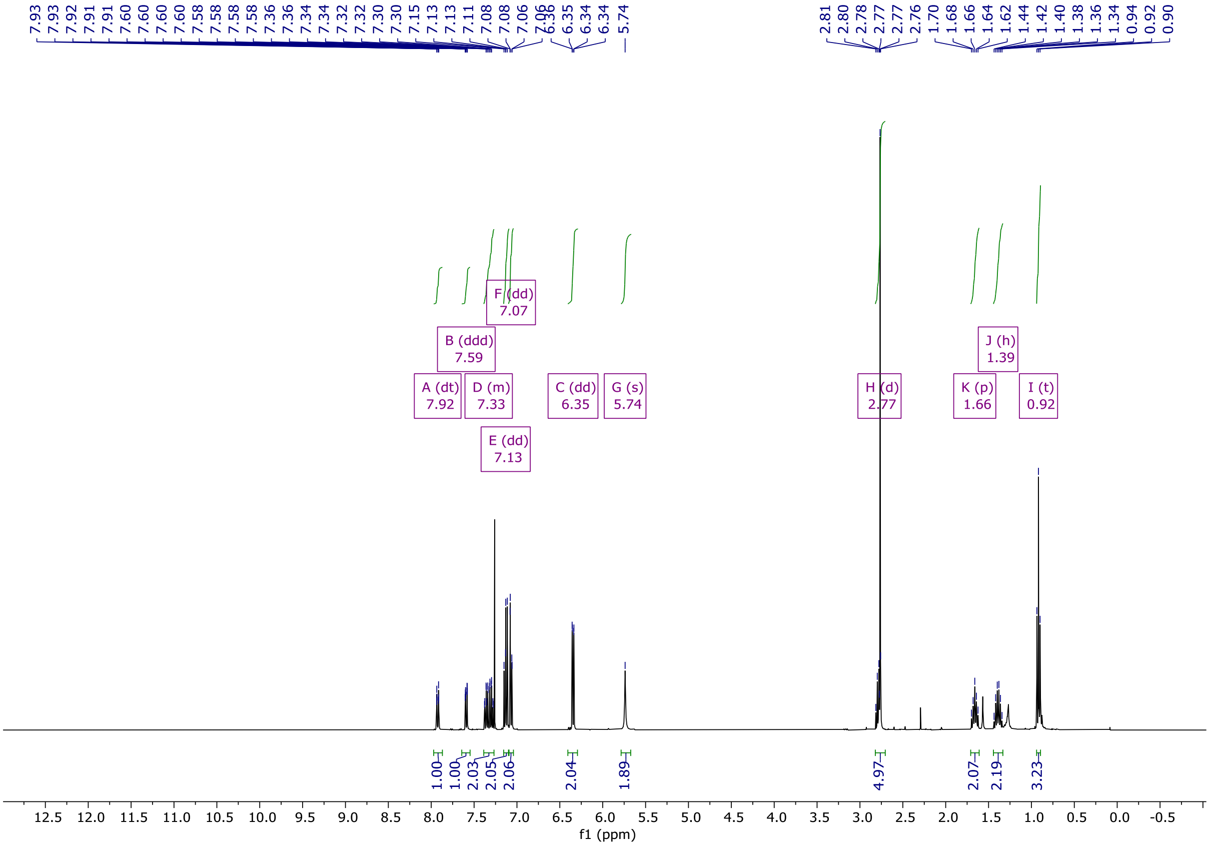

^{13}C{^{1}H} NMR (101 MHz, $CDCl_3$):

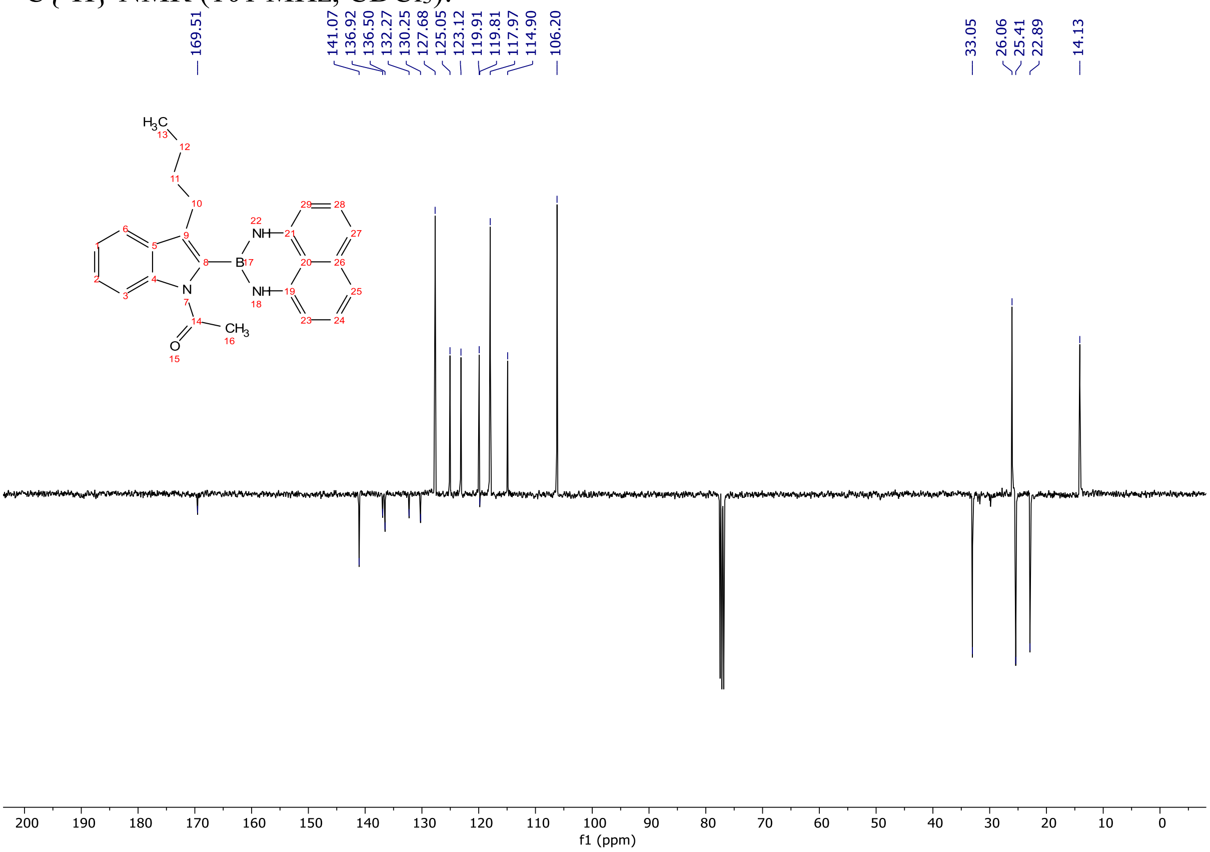

(5b) ^{1}H NMR (400 MHz,$CDCl_3$):

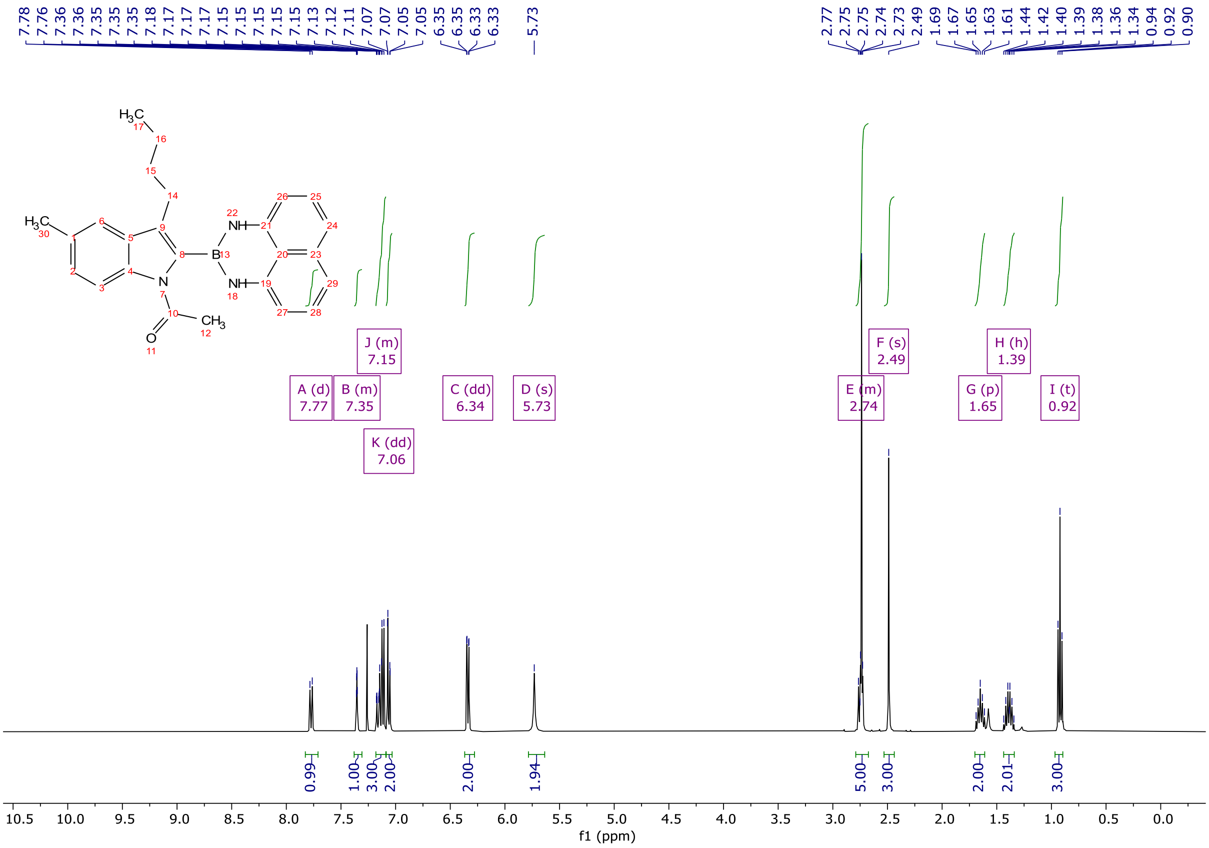

^{13}C{^{1}H} NMR (101 Mhz, $CDCl_3$):

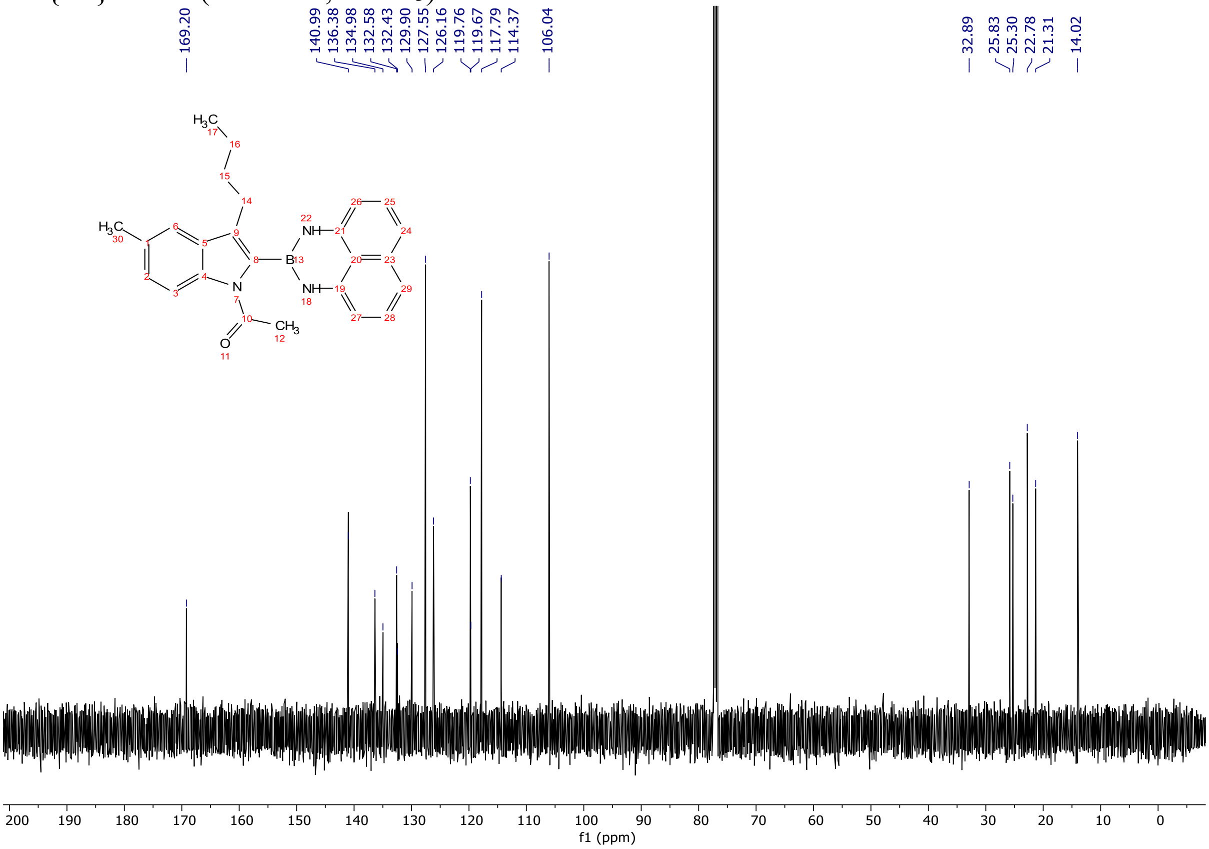

(5c) ^{1}H NMR (400 MHz, $CDCl_3$):

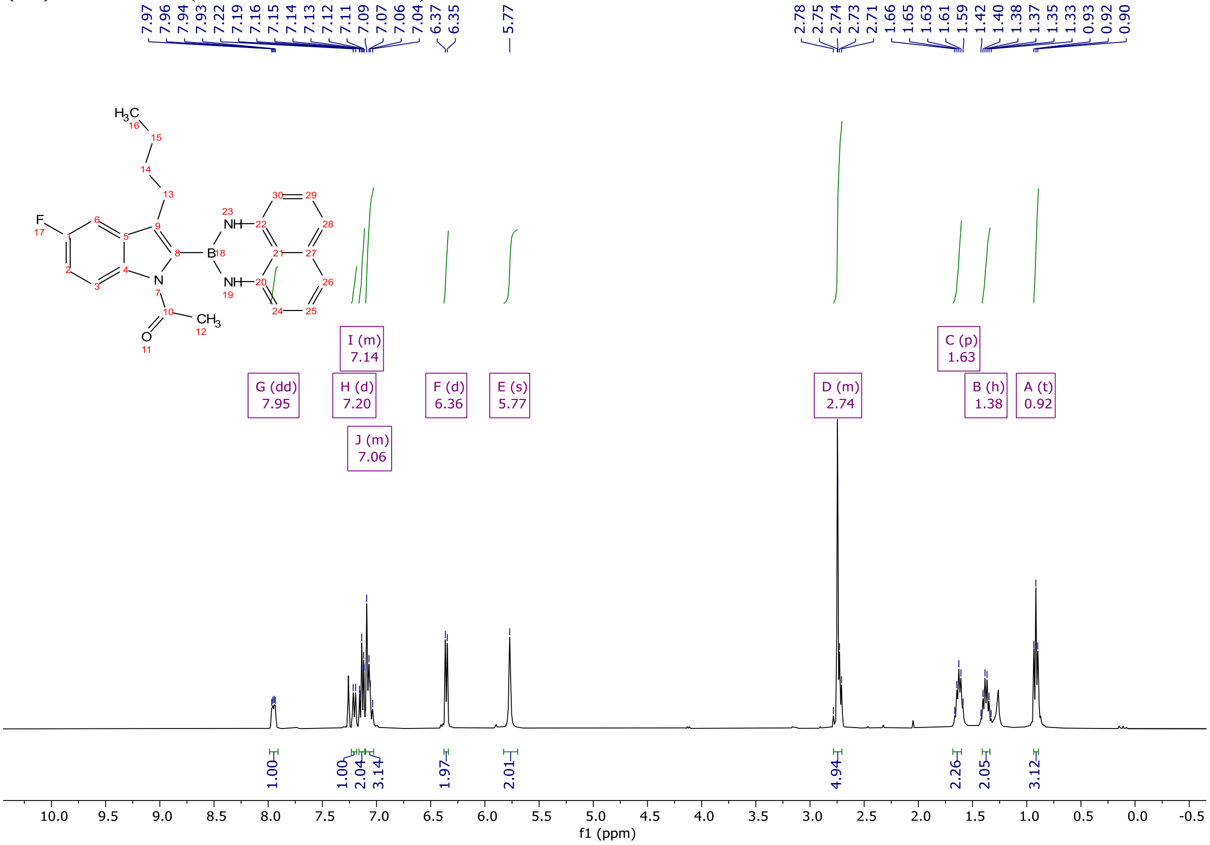

^{13}C DEPTQ (101 MHz, $CDCl_3$):

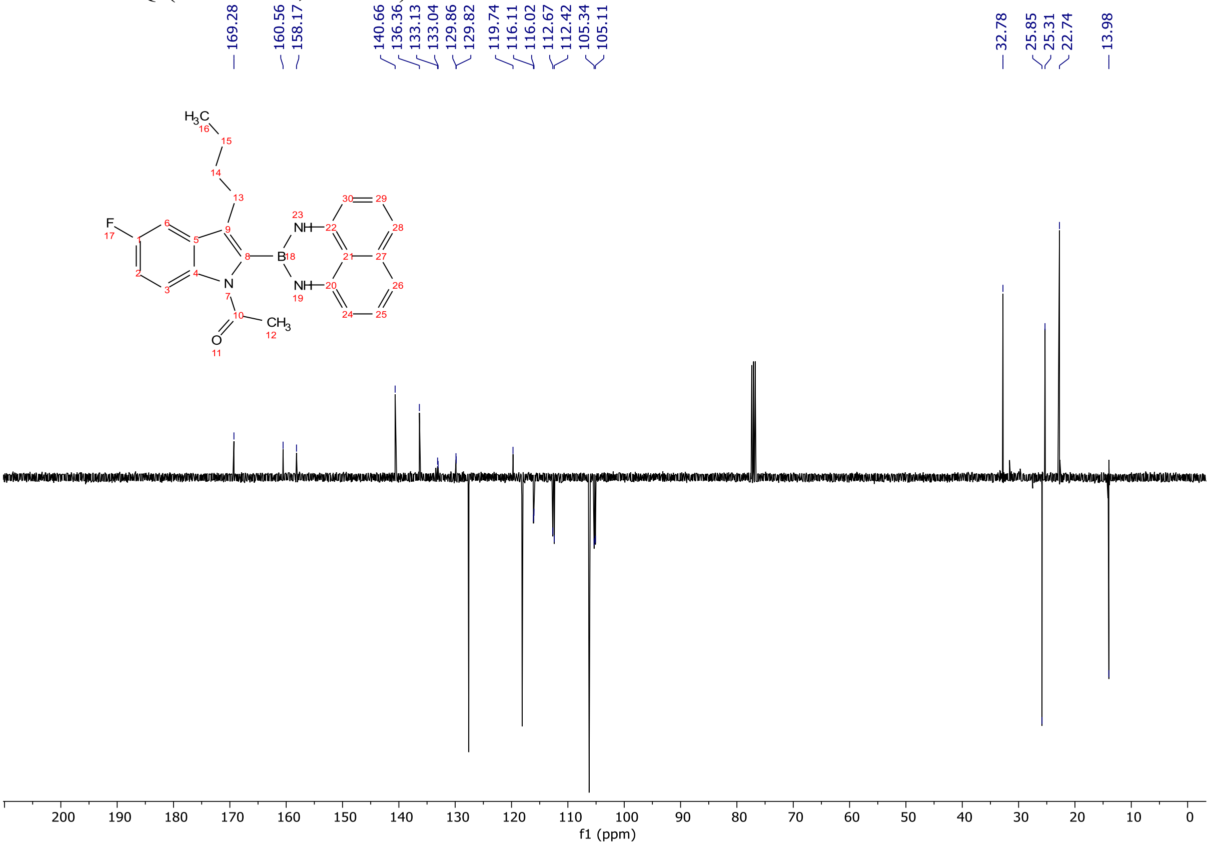

^{19}F NMR (376 MHz, $CDCl_3$):

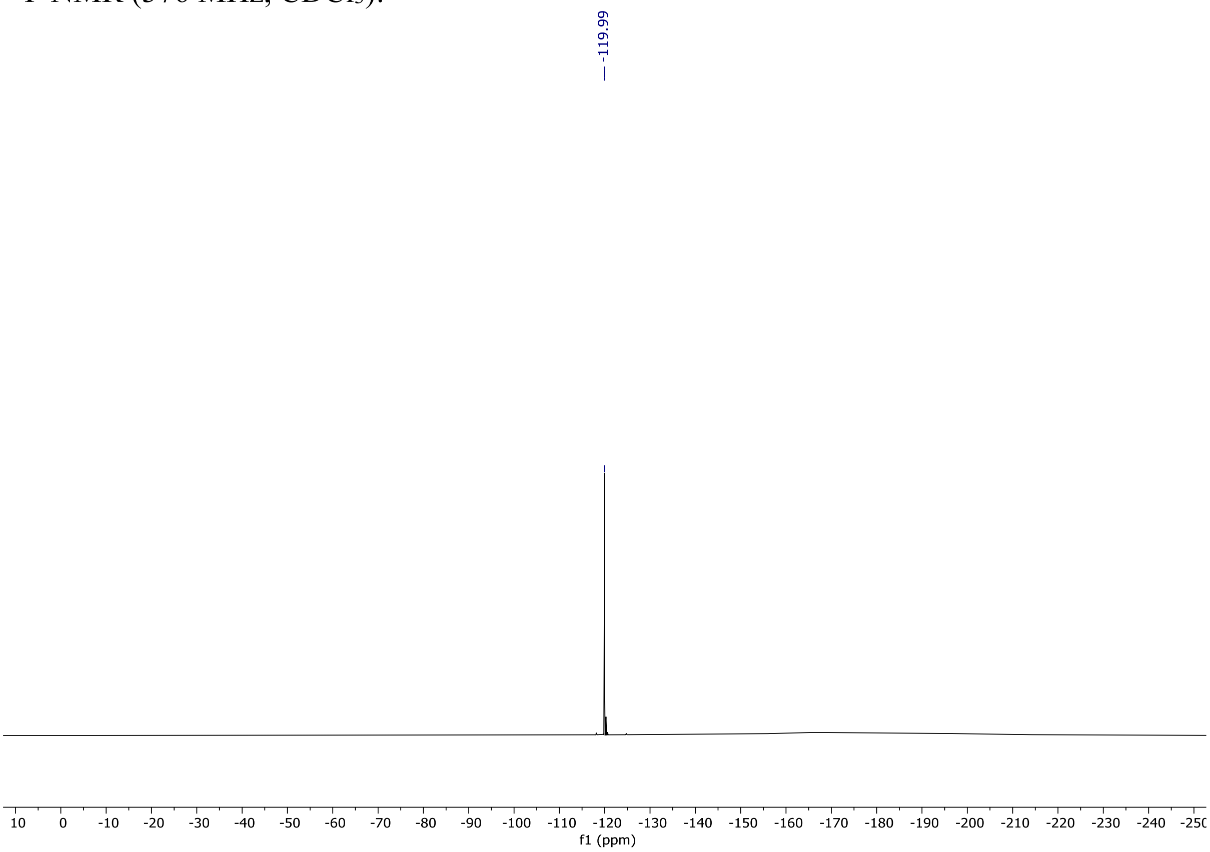

(5d) ^{1}H NMR (500 MHz, $CDCl_3$):

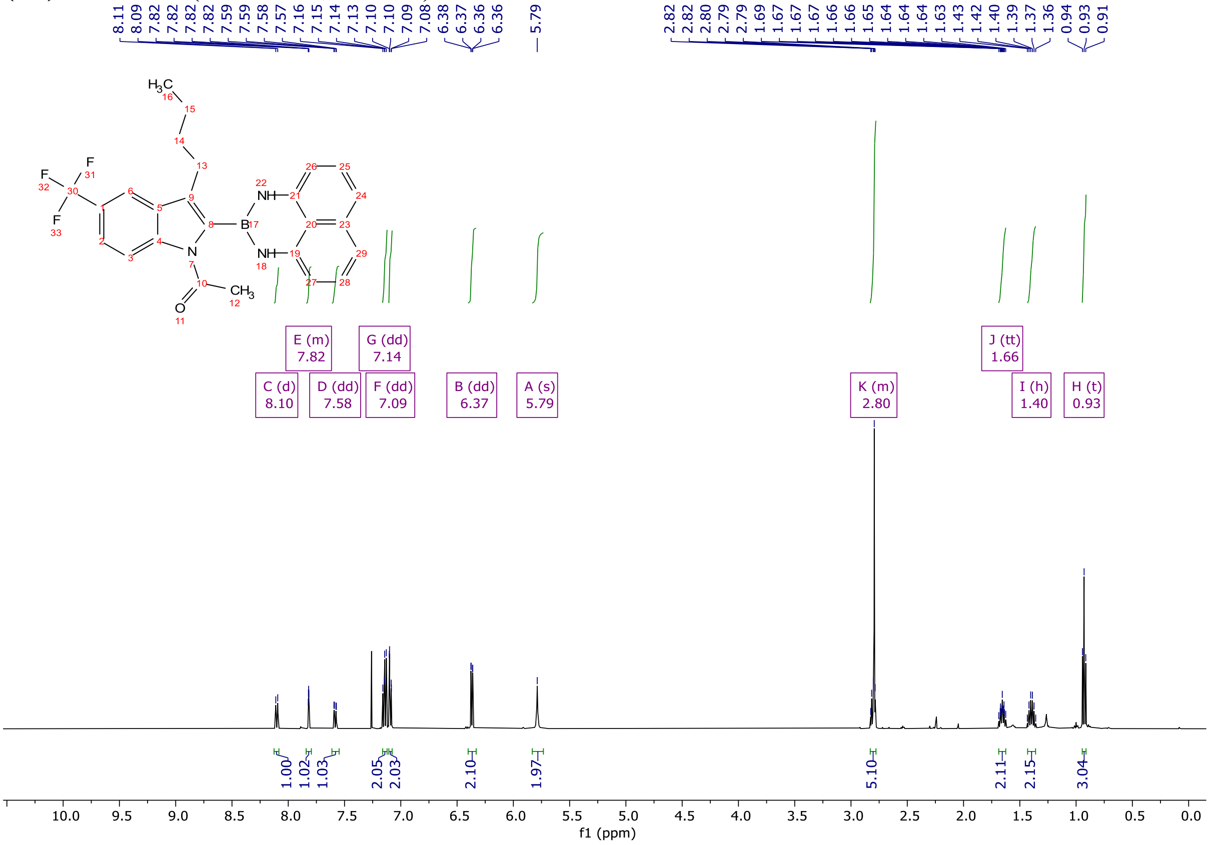

^{13}C{^{1}H} NMR (126 MHz, $CDCl_3$):

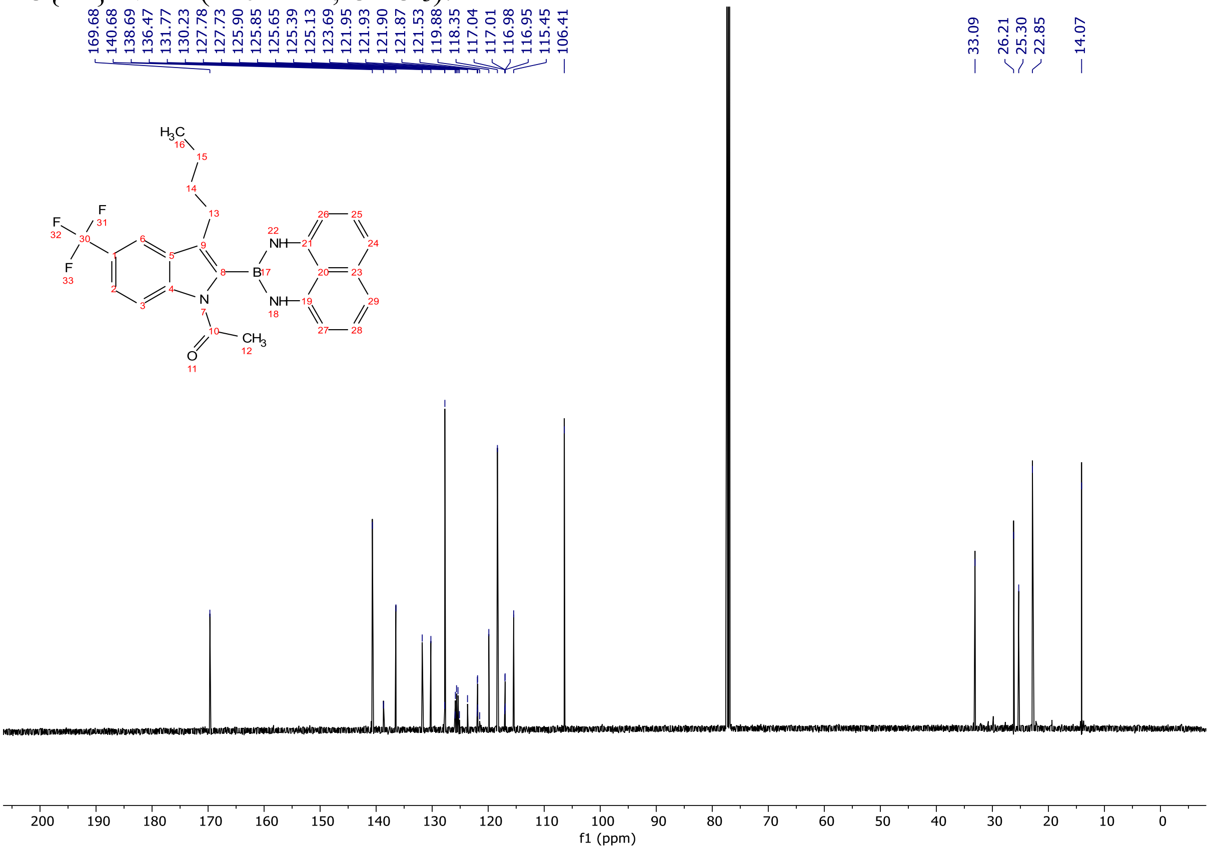

^{19}F NMR (377 MHz, $CDCl_3$):

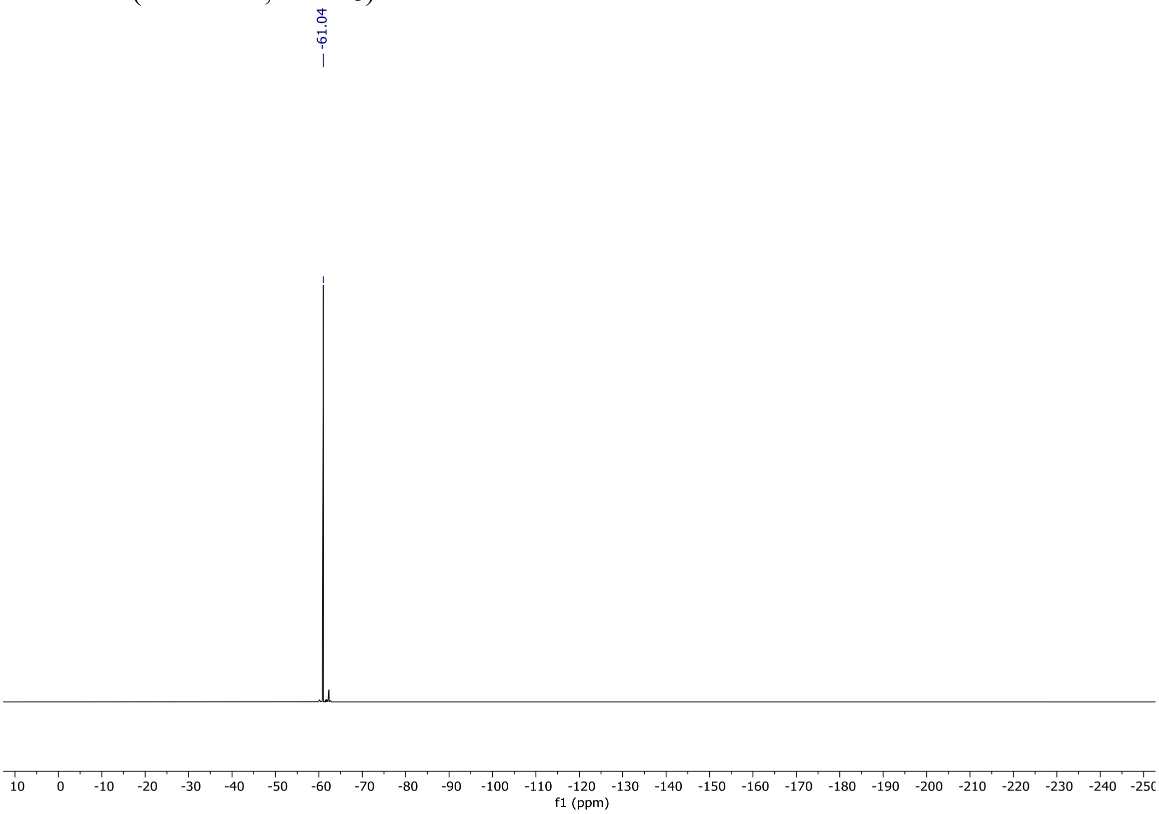

(5e) ^{1}H NMR (400 MHz, $CDCl_3$):

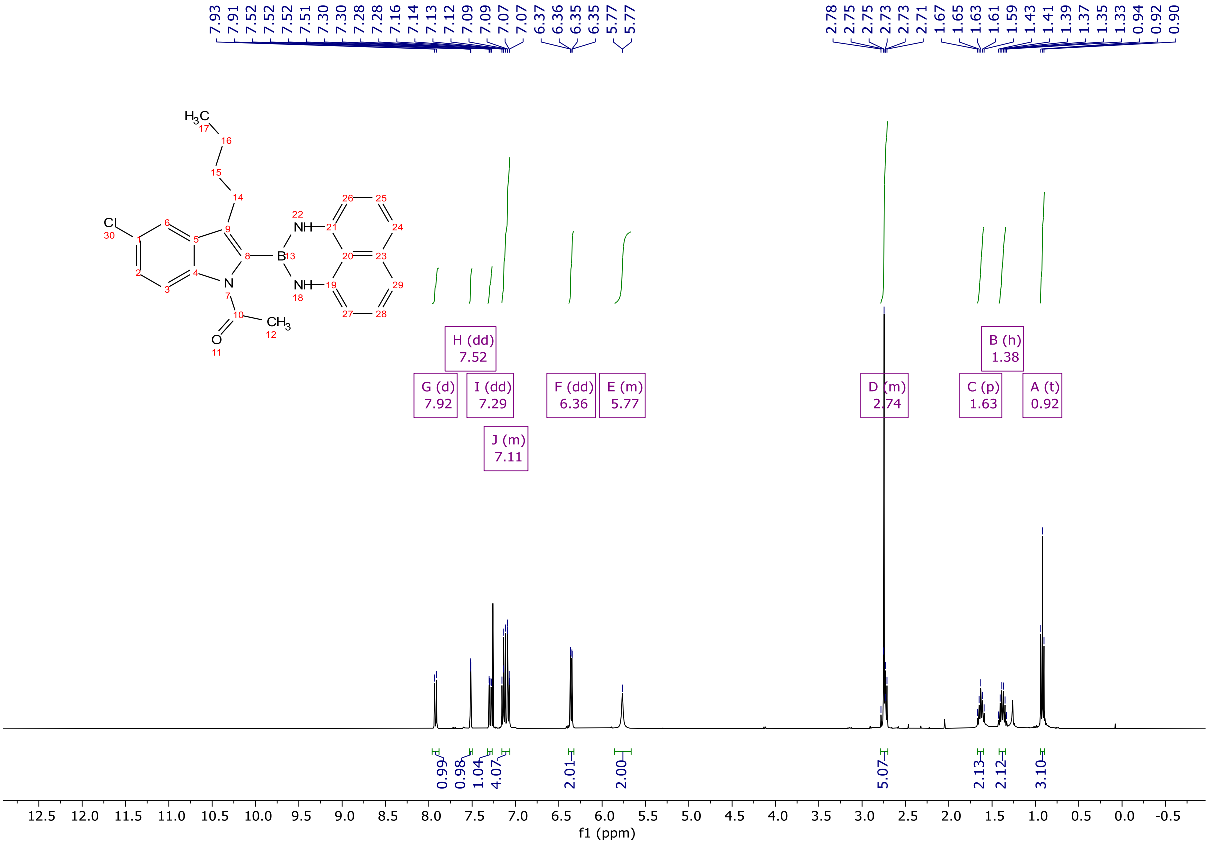

$^{13}C\{^{1}H\}$ NMR (101 MHz, $CDCl_3$):

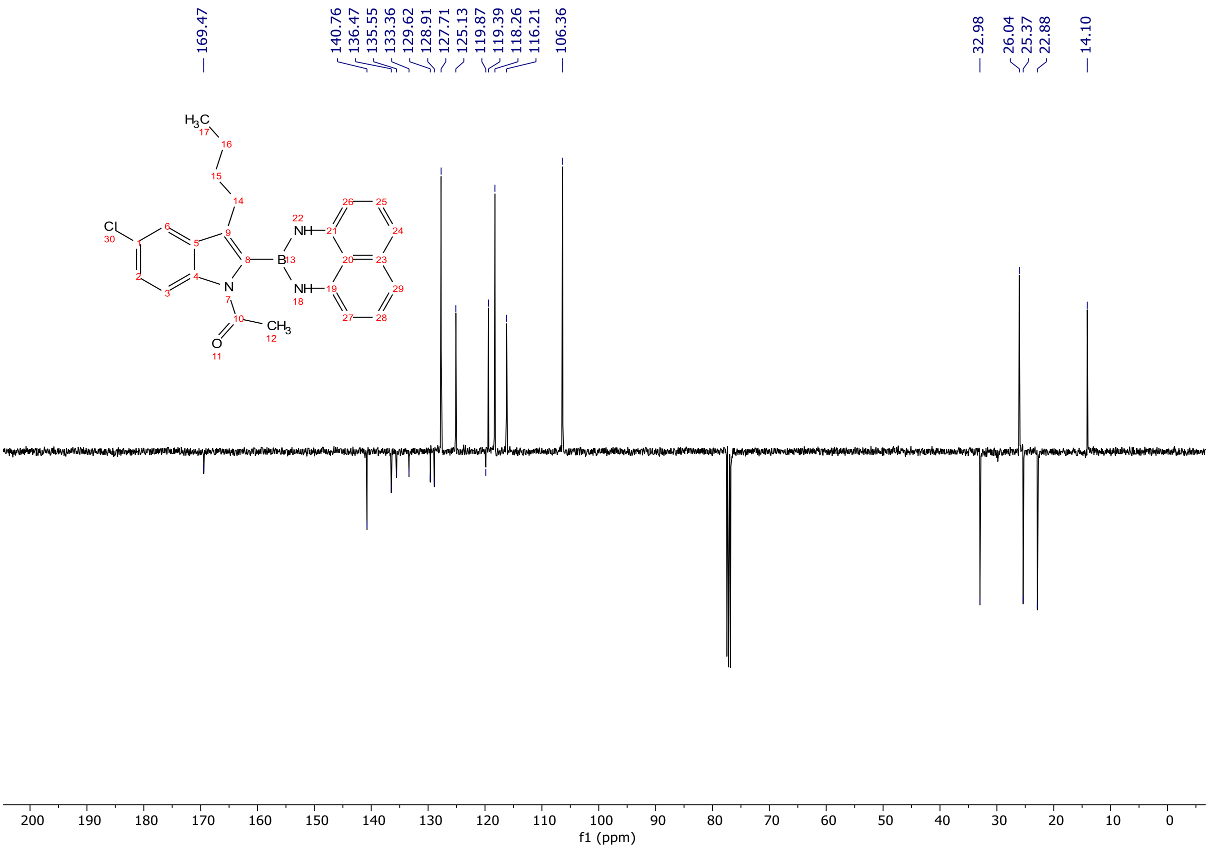

(5f) ^{1}H NMR (400 MHz, CDCl$_3$):

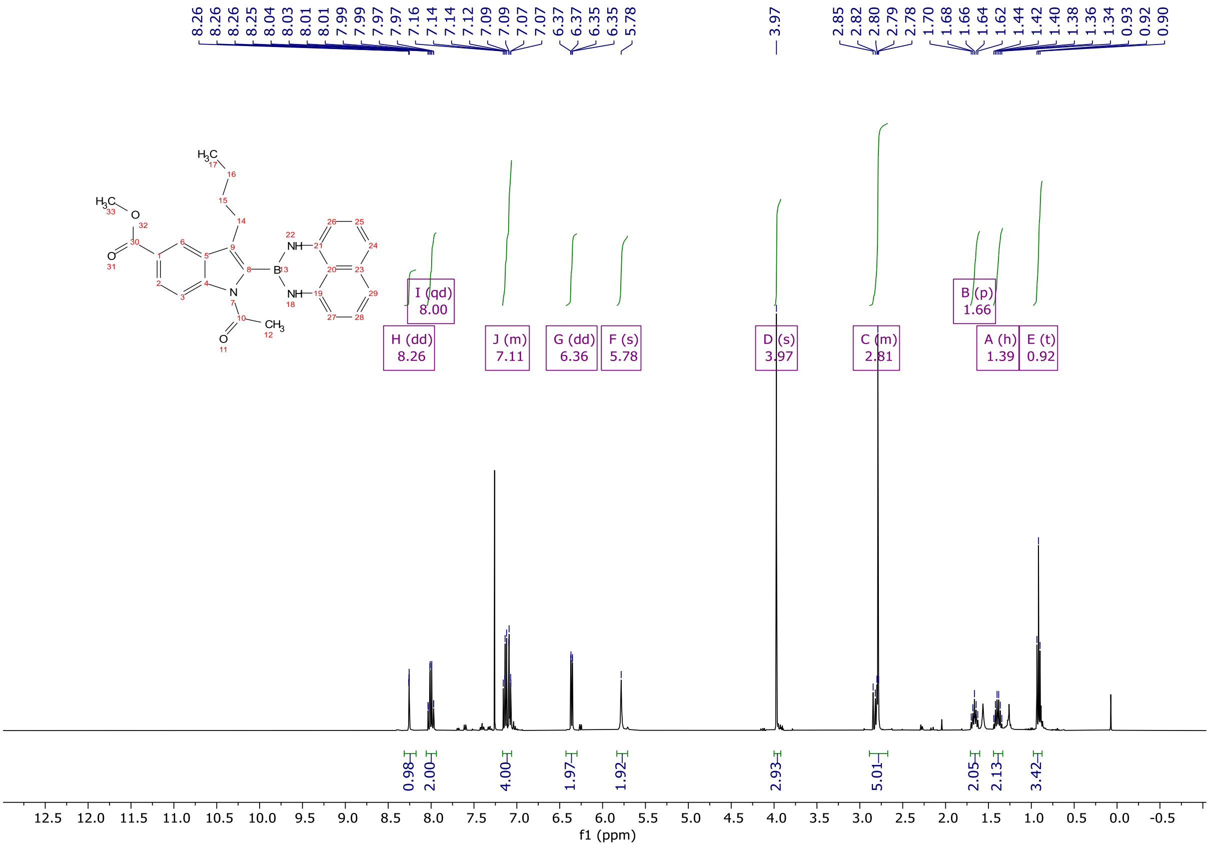

^{13}C DEPTQ (101 MHz, CDCl$_3$):

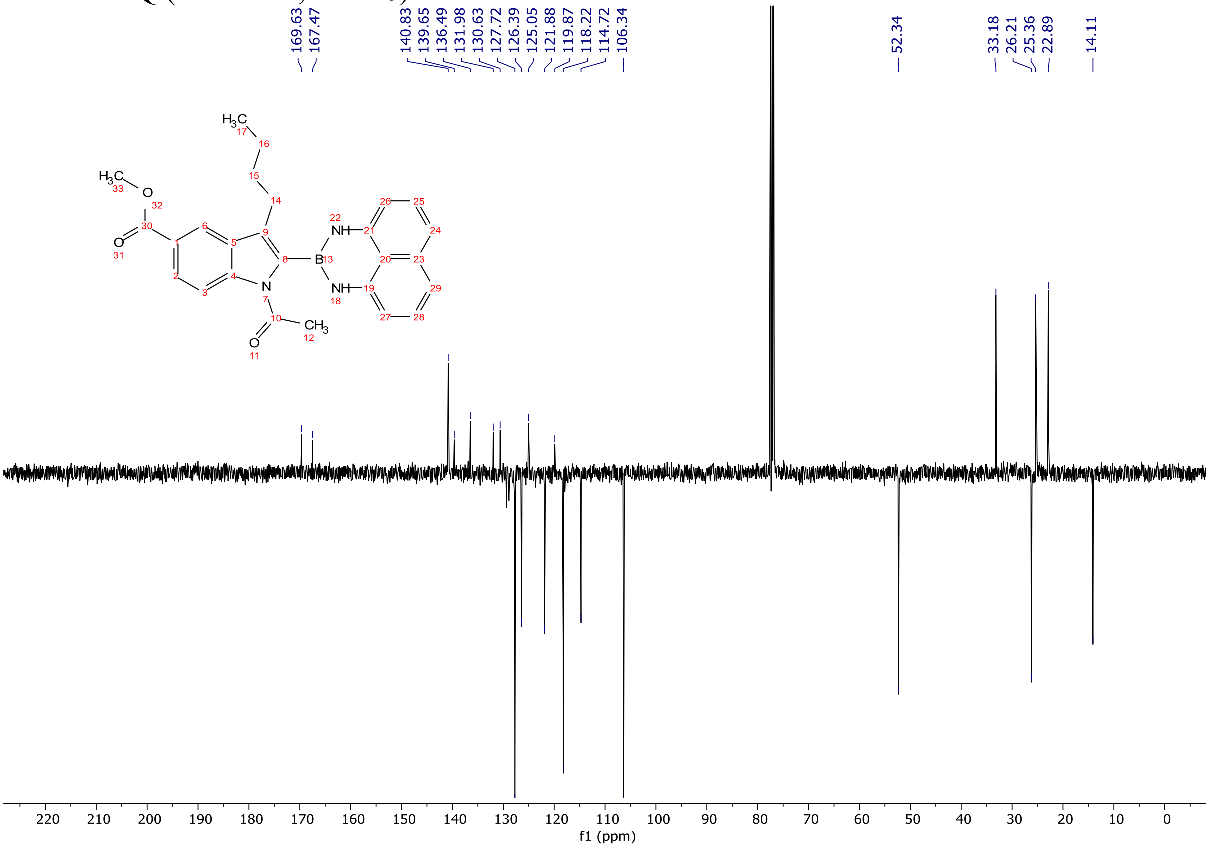

(5g) ^{1}H NMR (500 MHz, CDCl$_3$):

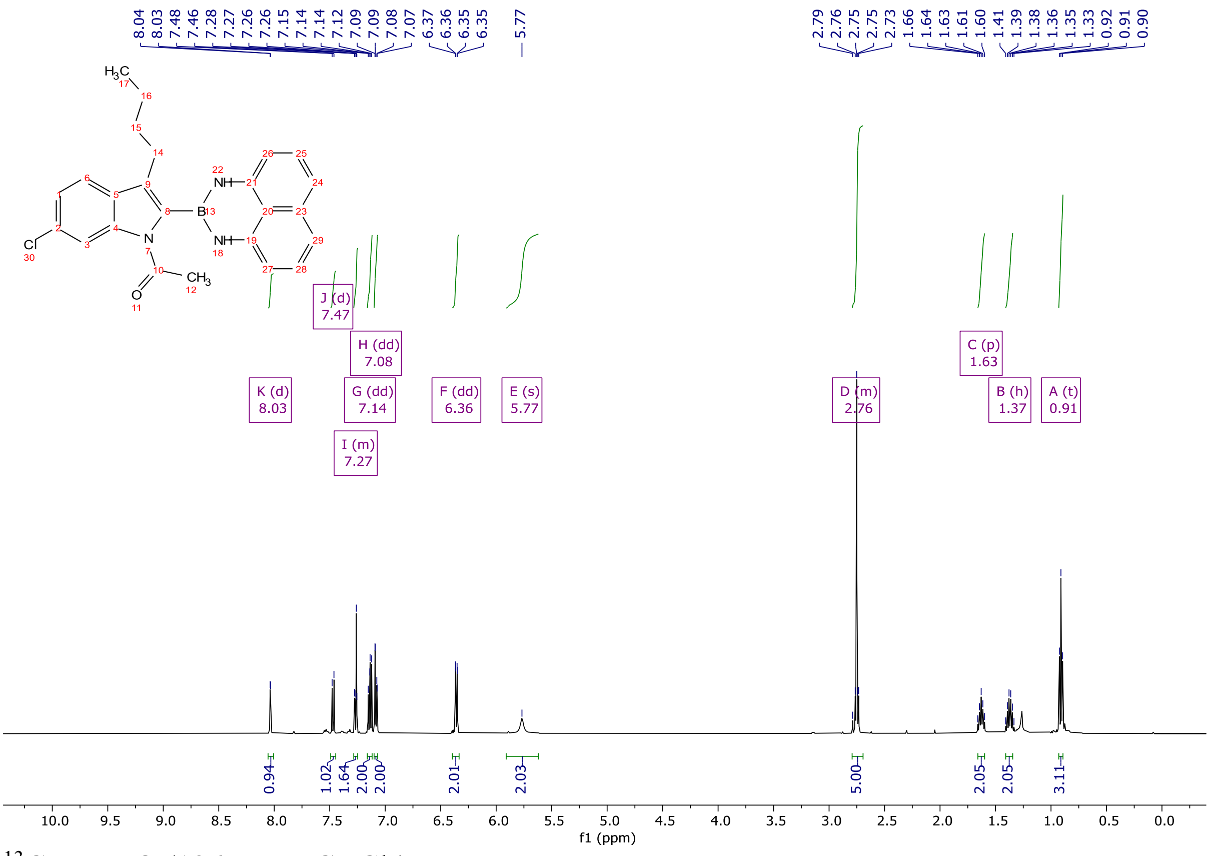

^{13}C DEPTQ (126 MHz, CDCl$_3$):

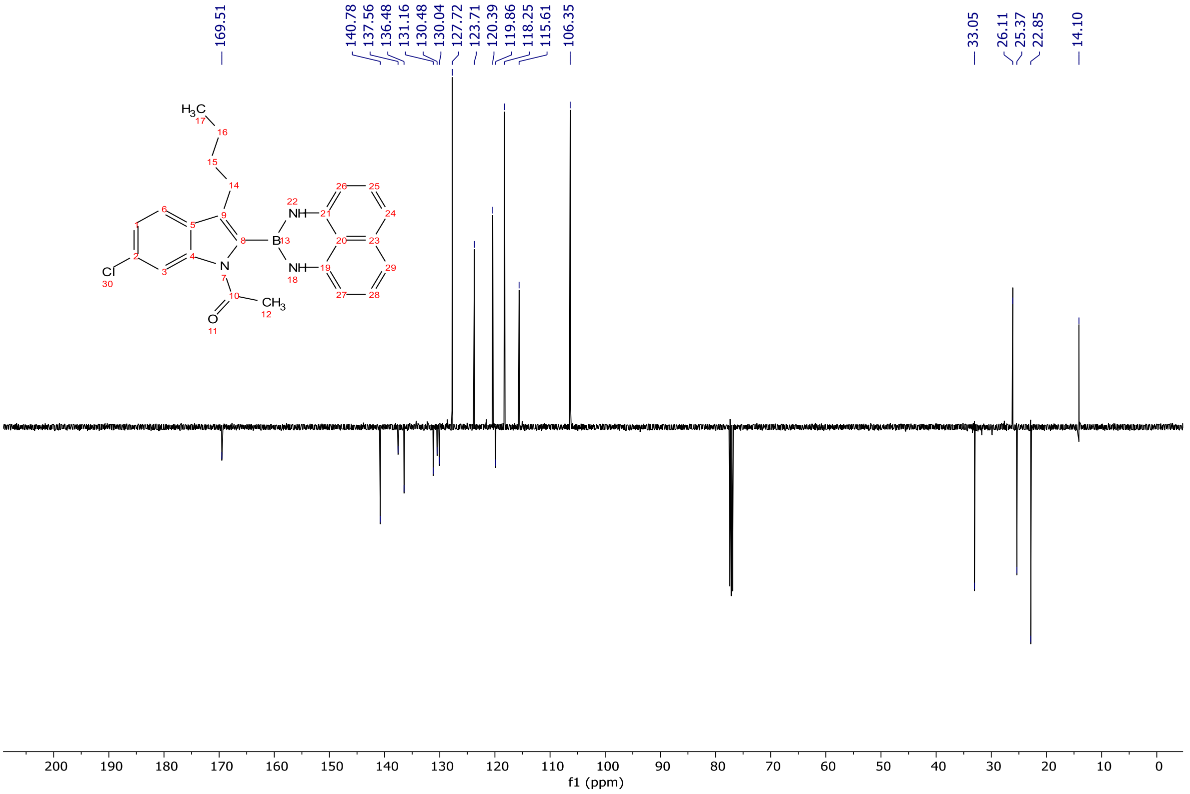

(5h) ^{1}H NMR (400 MHz, $CDCl_3$):

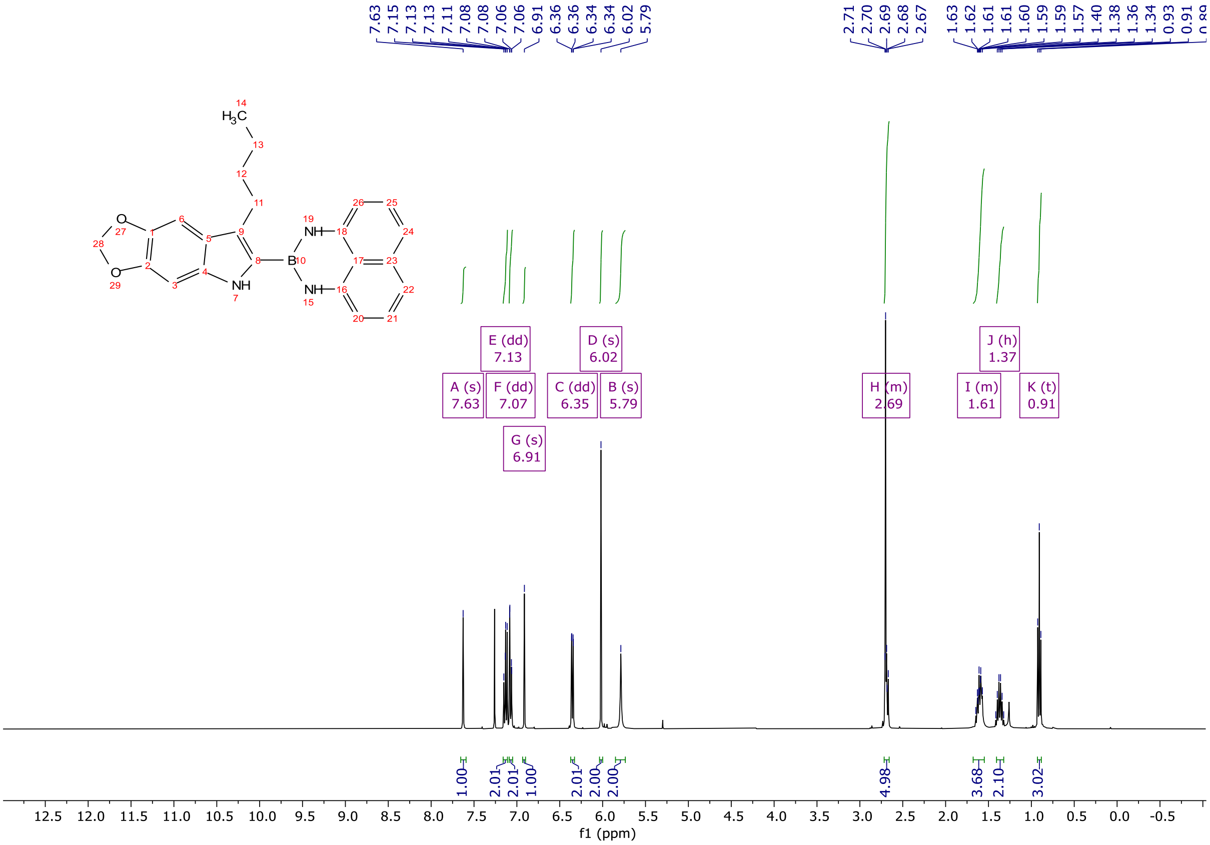

^{13}C DEPTQ (101 MHz, $CDCl_3$):

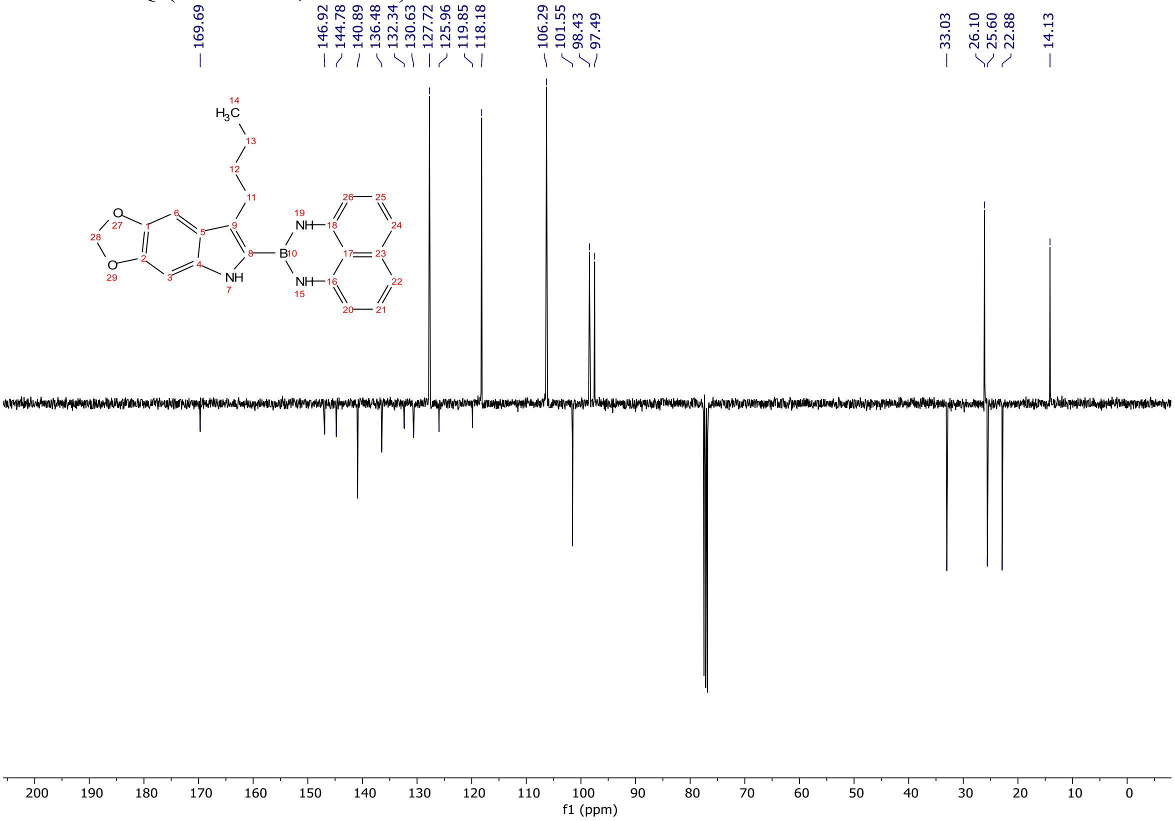

(5i) ^{1}H NMR (400 MHz, $CDCl_3$):

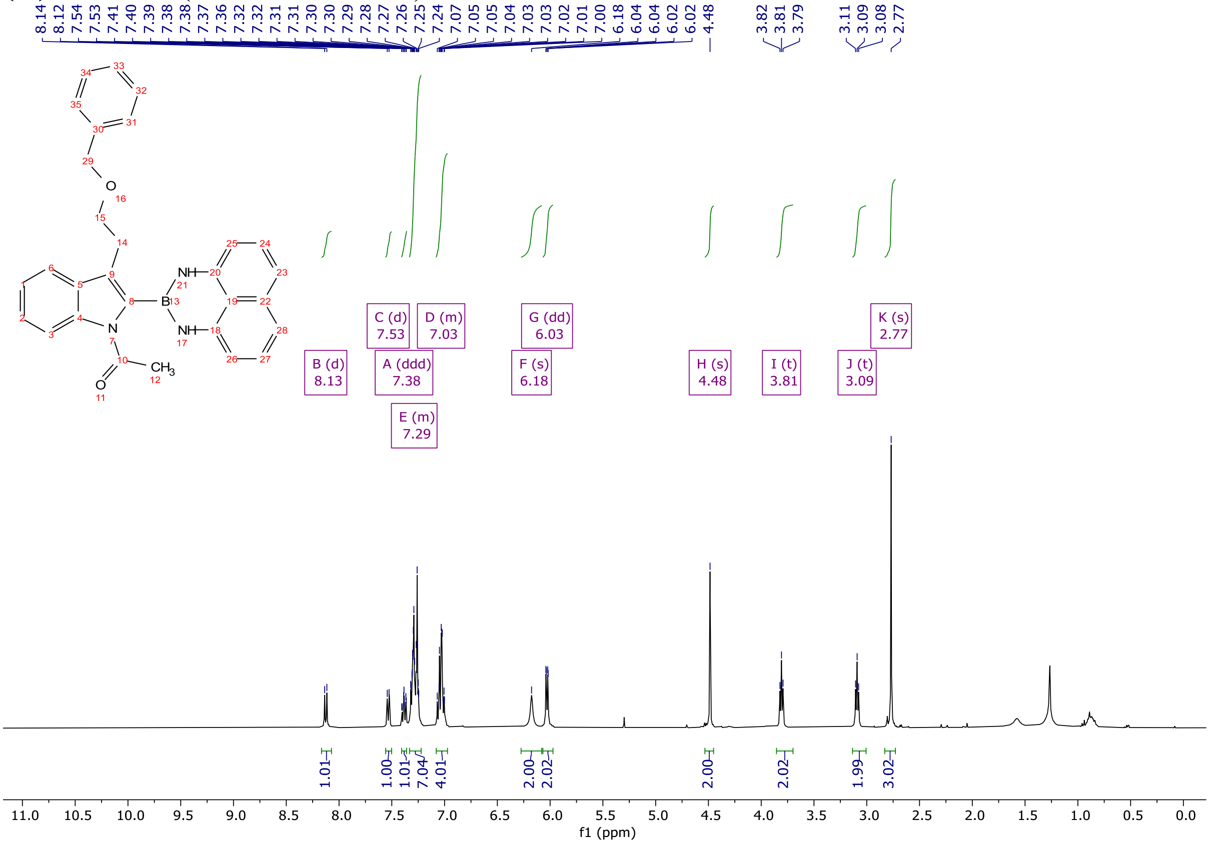

^{13}C{^{1}H} NMR (101 MHz, $CDCl_3$):

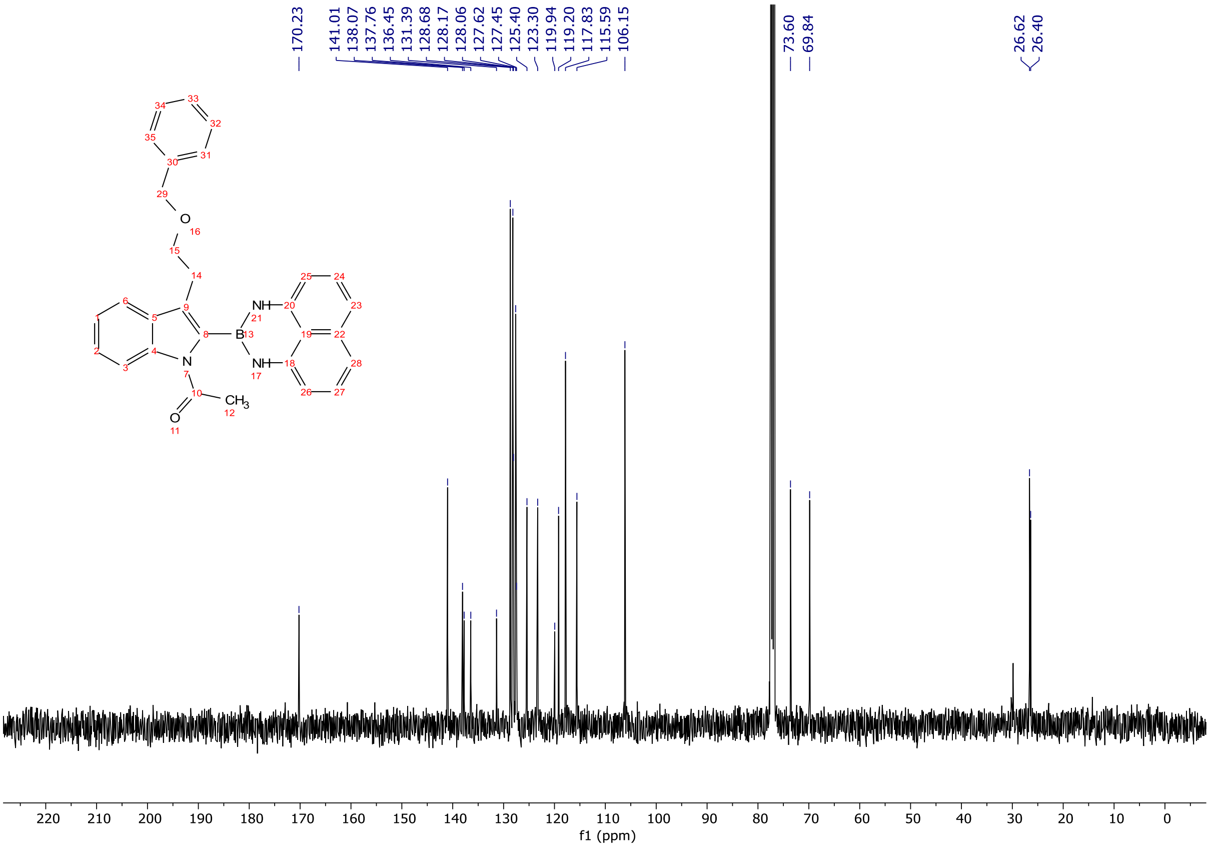

(5j) ^{1}H NMR (400 MHz, $CDCl_3$):

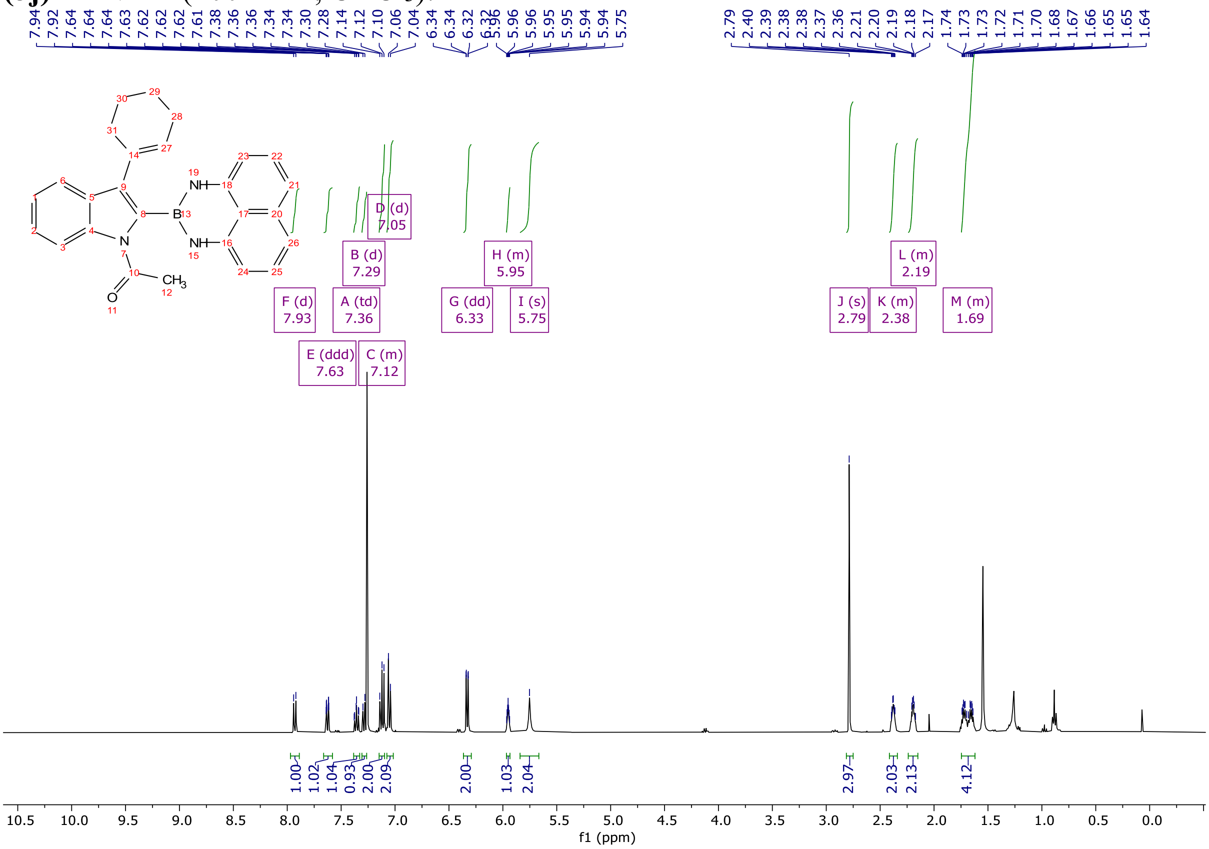

$^{13}C\{^{1}H\}$ NMR (101 MHz, $CDCl_3$):

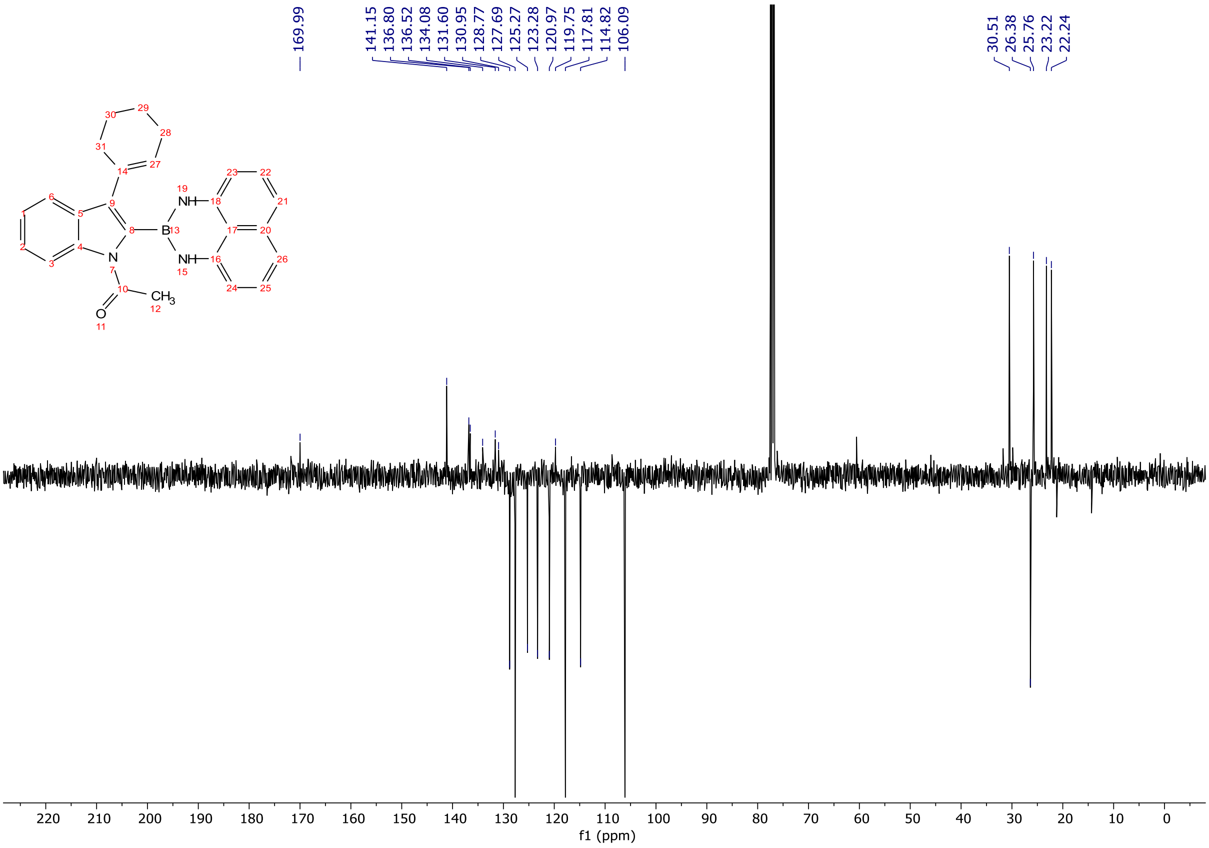

(5k) ^{1}H NMR (400 MHz, $CDCl_3$):

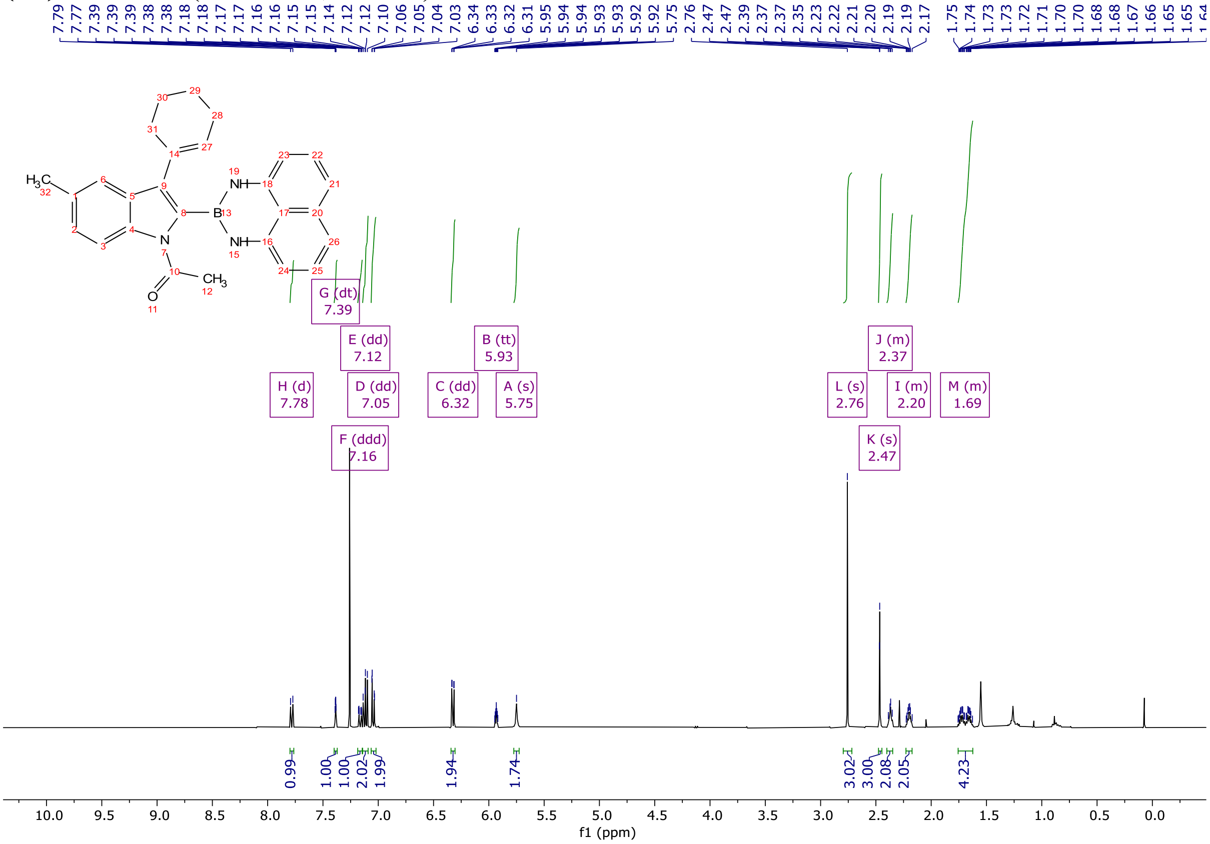

^{13}C DEPTQ (101 MHz, $CDCl_3$):

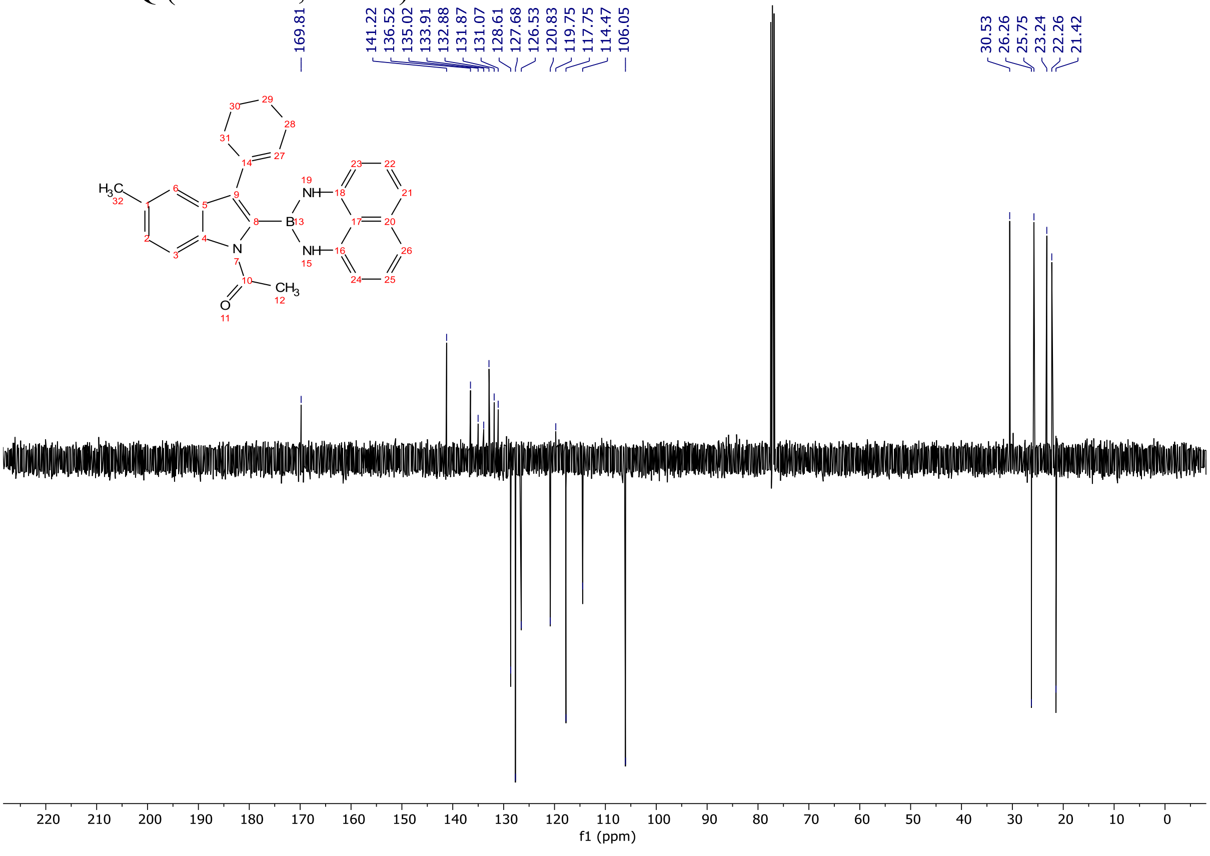

(5l) ^{1}H NMR (400 MHz, $CDCl_3$):

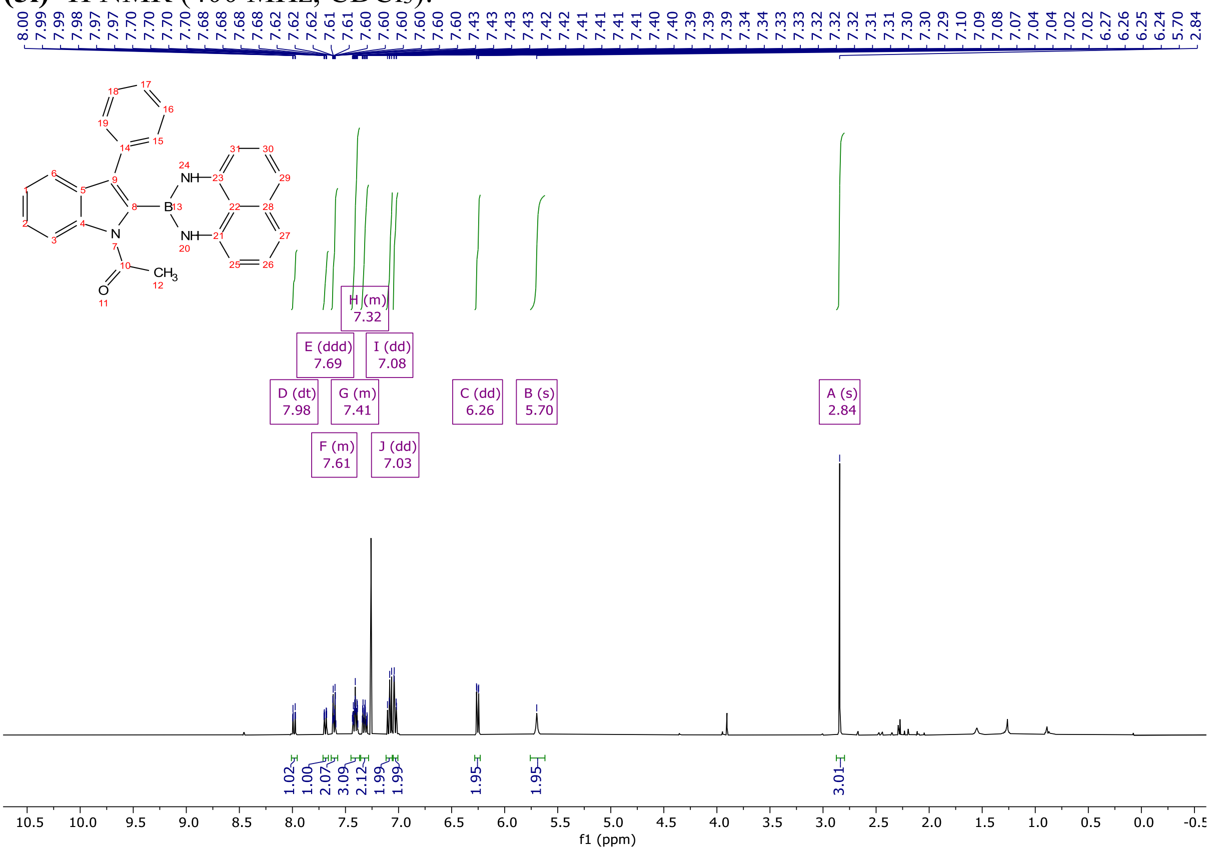

^{13}C DEPTQ (101 MHz, $CDCl_3$):

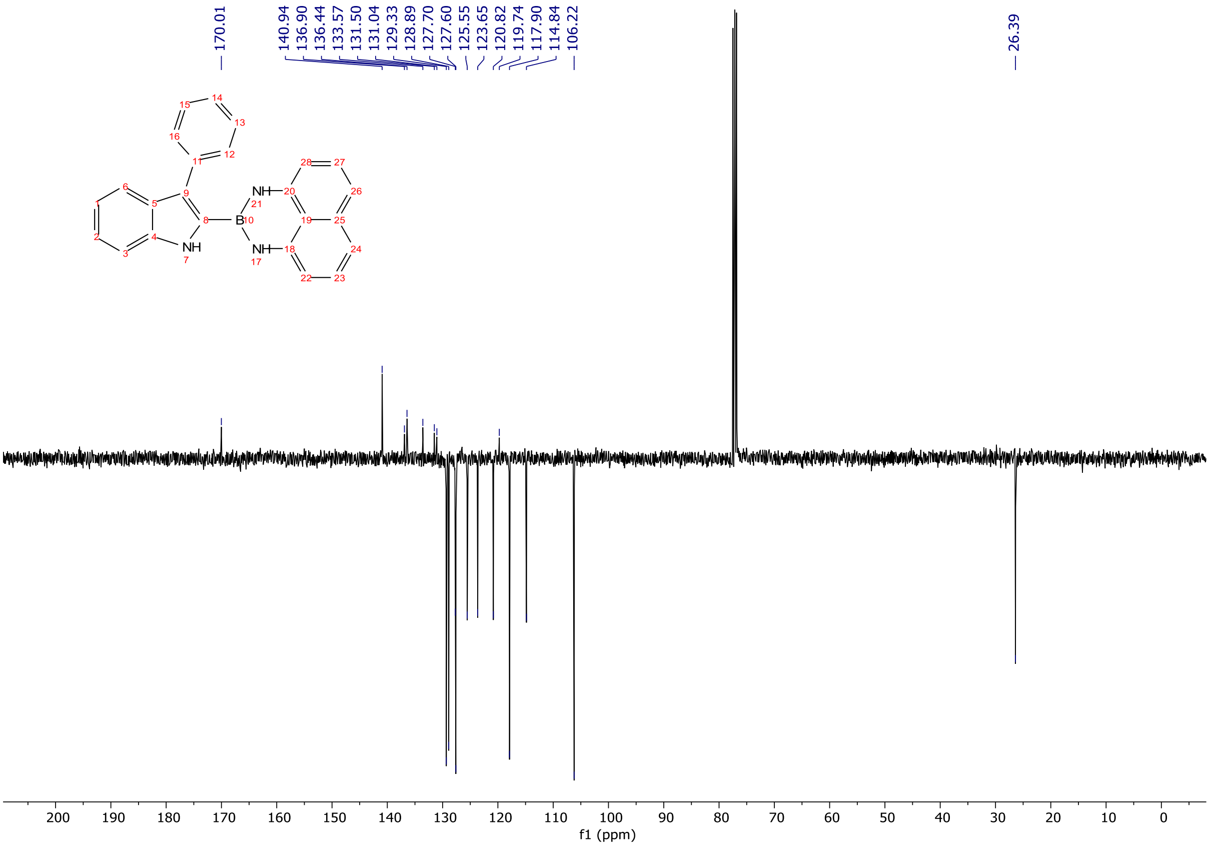

(7) ^{1}H NMR (400 MHz, $CDCl_3$):

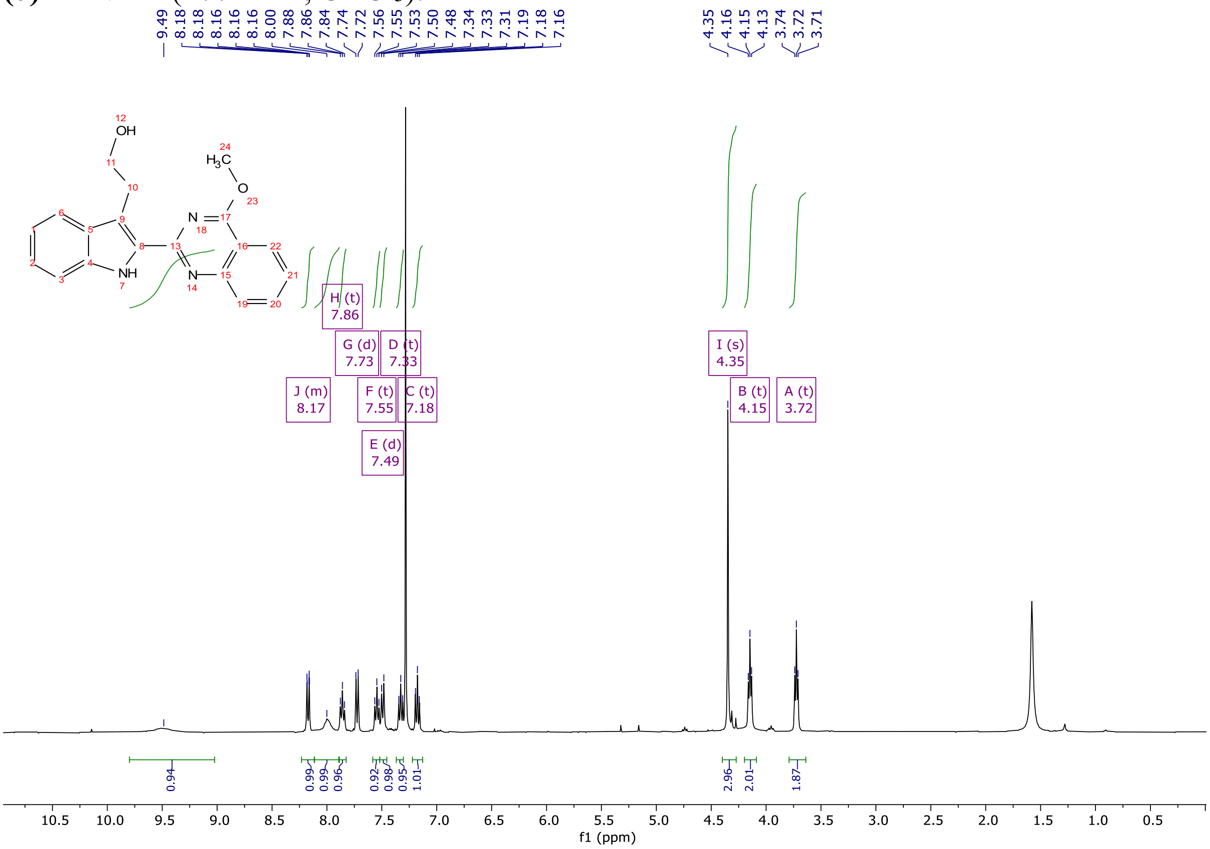

Supplement: Supplementary file 1 [file jo5c01781_si_001.pdf]
